# Molecular Characterization, Expression Analysis of Carotenoid, Xanthophyll, Apocarotenoid Pathway Genes, and Carotenoid and Xanthophyll Accumulation in *Chelidonium majus* L.

**DOI:** 10.3390/plants10081753

**Published:** 2021-08-23

**Authors:** Ramaraj Sathasivam, Hyeon Ji Yeo, Chang Ha Park, Minsol Choi, Haejin Kwon, Ji Eun Sim, Sang Un Park, Jae Kwang Kim

**Affiliations:** 1 Department of Crop Science, Chungnam National University, 99 Daehak-ro, Yuseong-gu, Daejeon 34134, Korea; ramarajbiotech@gmail.com (R.S.); guswl7627@gmail.com (H.J.Y.); parkch804@gmail.com (C.H.P.); 201802785@o.cnu.ac.kr (M.C.); kwonhaejin42@o.cnu.ac.kr (H.K.); 2 Division of Life Sciences, College of Life Sciences and Bioengineering, Incheon National University, Yeonsu-gu, Incheon 22012, Korea; ajaj7433@inu.ac.kr; 3 Department of Smart Agriculture Systems, Chungnam National University, 99 Daehak-ro, Yuseong-gu, Daejeon 34134, Korea

**Keywords:** *Chelidonium majus*, carotenoid pathway genes, xanthophyll pathway genes, apocarotenoid pathway genes, gene expression, carotenoid, xanthophyll

## Abstract

*Chelidonium majus* L. is a perennial herbaceous plant that has various medicinal properties. However, the genomic information about its carotenoid biosynthesis pathway (CBP), xanthophyll biosynthesis pathway (XBP), and apocarotenoid biosynthesis pathway (ABP) genes were limited. Thus, the CBP, XBP, and ABP genes of *C. majus* were identified and analyzed. Among the 15 carotenoid pathway genes identified, 11 full and 4 partial open reading frames were determined. Phylogenetic analysis of these gene sequences showed higher similarity with higher plants. Through 3D structural analysis and multiple alignments, several distinct conserved motifs were identified, including dinucleotide binding motif, carotene binding motif, and aspartate or glutamate residues. Quantitative RT-PCR showed that CBP, XBP, and ABP genes were expressed in a tissue-specific manner; the highest expression levels were achieved in flowers, followed by those in leaves, roots, and stems. The HPLC analysis of the different organs showed the presence of eight different carotenoids. The highest total carotenoid content was found in leaves, followed by that in flowers, stems, and roots. This study provides information on the molecular mechanisms involved in CBP, XBP, and ABP genes, which might help optimize the carotenoid production in *C. majus*. The results could also be a basis of further studies on the molecular genetics and functional analysis of CBP, XBP, and ABP genes.

## 1. Introduction

*Chelidonium majus* belongs to the Papaveraceae family, and it is widely found worldwide, including Asia, Europe, North America, and Northern Africa [1]. The aerial parts of this plant consist of isoquinoline alkaloids (berberine, chelerythrine, chelidonine, coptisine, sangui-narine, and stylopine), whereas herbs of *C. majus* consist of carotenoids, flavonoids, organic acids, and proteins [2,3,4,5,6,7]. The extract of *C. majus* has various antioxidant properties (elimination of reactive oxygen species (ROS), and protection from oxidative stress), with a wide range of medicinal properties such as analgesic, anti-inflammatory, antimicrobial, anti-spasmodic, antitumor, antiviral, choleretic, and hepatoprotective properties [1,2,5,8,9,10,11,12]. It has also been used for the treatment of asthma, cancer, chronic bronchitis, eczema, gastrointestinal diseases, liver disorder, oral infection, pain and nervous disorders, ringworm, and ulcer [5,9,13].

To date, many natural carotenoids were identified from medicinal plants. It is important to screen, identify, and analyze various types of carotenoids because of their pharmacological benefits [3]. Most studies have only focused on β-carotene; however, recently, other carotenoids also play a vital role in human diets, which increases their attention [14]. Carotenoids are the most important natural pigments that play a significant role in scavenging reactive oxygen species (ROS) and in protecting plant cells from photo-oxidative damage [15]. It has a wide range of health benefits such as immunomodulatory, anticancer, antibacterial, antiaging, antidiabetic, anti-inflammatory, and neuroprotective effects [16,17,18,19,20,21]. In addition, carotenoids might also help to enhance the immune system [22].

**Figure 1:**
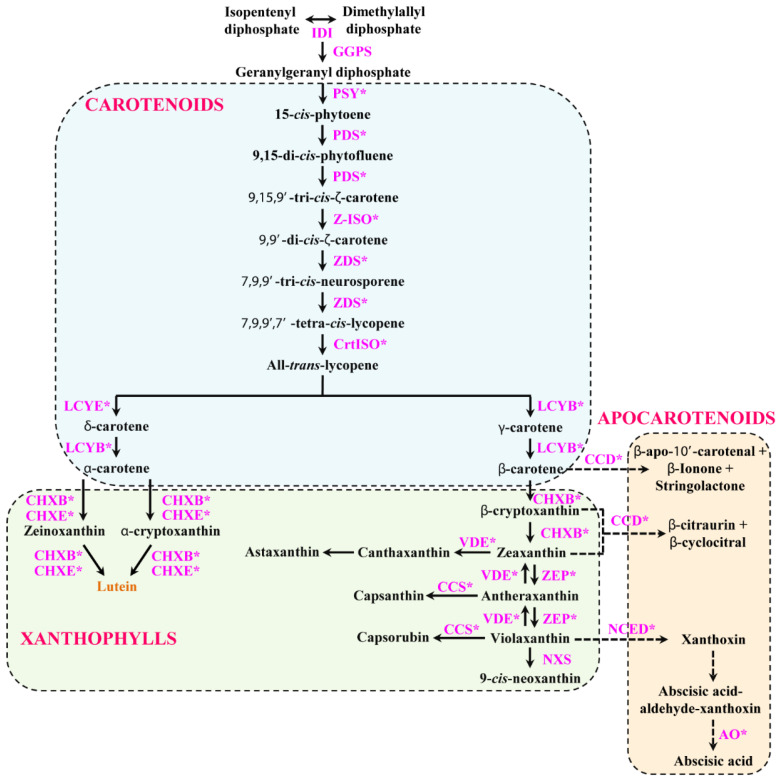
Proposed CBP, XBP, and ABP in *Chelidonium majus*. Enzymes are shown in pink. Asterisk denotes the gene used for identification, characterization, and gene expression studies. Solid black arrows represent biosynthesis and dotted black arrows represent the degradation of carotenoids.

Recently, several reviews have been published regarding the gene responsible for the regulation of the carotenoid biosynthesis pathway (CBP), xanthophylls biosynthesis pathway (XBP), and apocarotenoid biosynthesis pathway (ABP) genes in plants [15,23] (Figure 1). Among these, the foremost is CBP, the genes involved in this pathway have been identified and characterized, such as *Phytoene synthase* (*PSY*) in bread wheat, citrus (*Citrus unshiu* Marc., *Citrus sinensis* Osbeck, *Citrus limon* Burm.f.), Hong Kong kumquat, *Ixeris dentate*, melon, *Scutellaria baicalensis*, summer squash, and sunflower [24,25,26,27,28,29,30,31,32]; *Phytoene desaturase* (*PDS*) in common Andrographis herb, citrus (*Citrus unshiu* Marc., *Citrus sinensis* Osbeck, *Citrus limon* Burm.f.), *I. dentate*, *S. baicalensis*, and wolfberry [26,29,31,33,34]; *ζ-carotene isomerase* (*Z-ISO*) in *Arabidopsis* and *Osmanthus fragrans* [35,36]; *ζ-carotene desaturase* (*ZDS*) in carrot, Chinese cabbage, citrus (*Citrus unshiu* Marc., *Citrus sinensis* Osbeck, *Citrus limon* Burm.f.), *Ficus carica*, *S. baicalensis*, and wolfberry [26,31,33,37,38,39,40]; carotenoid isomerase (*CrtISO*) in cabbage, citrus (*Citrus sinensis* L Osbeck; cv. “Pera”), and wolfberry [33,41,42]; *lycopene β-cyclase* (*LCYB*) in *Arabidopsis*, citrus (*Citrus unshiu* Marc., *Citrus sinensis* Osbeck, *Citrus limon* Burm.f.), *I. dentate*, maize, papaya, persimmon, strawberry, sweet range, and wheat [26,29,43,44,45,46,47,48,49,50]; *lycopene ε-cyclase* (*LCYE*) in *Arabidopsis*, citrus (*Citrus unshiu* Marc., *Citrus sinensis* Osbeck, *Citrus limon* Burm.f.), and strawberry [26,43,50]. The second is the XBP, these pathway genes have been identified and characterized in plants such as *β-ring carotene hydroxylase* (*CHXB*) in citrus (*Citrus unshiu* Marc., *Citrus sinensis* Osbeck, *Citrus limon* Burm.f.), *I. dentate*, *S. baicalensis*, and strawberry [26,29,31,50]; *ε-ring carotene hydroxylase* (*CHXE*) in *I. dentate* [29]; *zeaxanthin epoxidase* (*ZEP*) in citrus (*Citrus unshiu* Marc., *Citrus sinensis* Osbeck, *Citrus limon* Burm.f.), *I. dentate*, and *S. baicalensis* [26,29,31]; *violaxanthin de-epoxidase* (*VDE*) in *Cucumis sativus*, *Phyllostachys edulis* [51,52]; *capsanthin-capsorubin synthase* (*CCS*) in *Lilium lancifolium* [53]. In another branch, the apocarotenoids were synthesized and the enzyme responsible for ABP genes have been identified and characterized in plants such as *CCD* in apple, chrysanthemum, citrus (*Citrus sinensis L. Osbeck*, *Citrus clementina*, *C. clementina* × *Citrus reticulata*), *Crocus ancyrensis*, osmanthus, rose, and *S. baicalensis* [54,55,56,57,58]; *9-cis-epoxycarotenoid dioxygenase* (*NCED*) in *Arabidopsis*, and *S. baicalensis* [31,59]; *aldehyde oxidase* (*AO*) in *Zea mays* [60].

Recently, a transcriptomic analysis of *C. majus* elaiosomes and seeds was carried out, together with the expression analysis of genes related to the fatty acid biosynthetic pathway [61]. In another study, a transcriptomic analysis of *C. majus* roots and leaves was conducted to analyze the expression profile of genes involved in benzylisoquinoline alkaloid biosynthetic pathway [62]. Khodabande et al. [9] analyzed the antioxidant activity, total protein, and soluble sugar content of *C. majus* leaf extracts during different growth stages. However, there are no published reports regarding the characterization and gene expression of CBP, XBP, and ABP genes (*CmPSY*, *CmPDS*, *CmZDS*, *CmCrtISO*, *CmLCYB*, *CmLCYE*, *CmCHXB*, *CmCHXE*, *CmZEP*, *CmCCD*, and *CmNCED*) and carotenoid accumulation in different organs (flowers, stems, roots, and leaves) of *C. majus*. Therefore, we analyzed the gene expression responses of CBP, XBP, and ABP genes in the different organs of *C. majus* using quantitative reverse-transcription polymerase chain reaction (qRT-PCR). Eight carotenoid compounds in the different organs of *C. majus* were also quantified using HPLC. The results can increase our understanding of CBP, XBP, and ABP genes and allow us to explore the strategies that could improve the anticarcinogenic properties of *C. majus*.

## 2. Results and Discussion

### 2.1. Identification, Protein Nomenclature, and Sequence Analysis of CBP, XBP, and ABP Genes

From the transcriptomic data, the CBP, XBP, and ABP genes of *C. majus* were identified. The identified genes were used as queries to search using the BLASTN program from the NCBI database. The BLASTN results showed that all identified genes had a high similarity with their corresponding sequences in higher plants. Then, the identified sequences were subjected to NCBI’s open reading frame (ORF) finder program to recognize whether the CBP gene has full ORFs. The genes with maximum nucleotide lengths were taken and subjected to structural and functional analysis. In addition, the genes that do not have full ORFs were also taken for structural and functional studies. A total of 15 CBP, XBP, and ABP genes were identified; among these 11 full ORFs (*CmPSY*, *CmPDS*, *CmZDS*, *CmCrtISO*, *CmLCYB*, *CmLCYE*, *CmCHXB*, *CmZEP*, *CmVDE*, *CmCCD*, and *CmAO*) were determined, whereas *CmZ-ISO*, *CmCHXE*, *CmCCS*, and *CmNCED* possess partial ORFs (Table 1 and Figure S1). The predicted MWs and the estimated isoelectric points of the CBP, XBP, and ABP genes are shown in Table 1. The predicted MW was reported in other plant species, such as *Brassica napus* [63], *I. dentate* [29], *Lycium chinenses* [33], *Nasturtium officinale* (unpublished data), and *S. baicalensis* [31,57]. Among the identified CBP, XBP, and ABP genes, none of these genes have a transmembrane region. Similar results were obtained in higher plants, such as *Musa acuminata* [64], *Brassica napus* [65], *N. officinale* (unpublished data), *Triticum aestivum* [24], and green algae *Tetraselmis suecica* [21], which do not possess any transmembrane regions in their CBP genes.

Signal IP analyses showed that *CmCrtISO* had the maximum original shearing site (C score) values, followed by *CmPSY*, *CmZDS*, *CmLCYE*, *CmVDE*, *CmCHXE*, *CmLCYB*, *CmZ-ISO*, *CmCHXB*, *CmCCS*, *CmZEP*, *CmPDS*, *CmAO*, *CmNCED*, and *CmCCD*, whereas *CmZDS* had the highest maximum synthesized shearing site (Y score) values, followed by *CmCrtISO*, *CmPSY*, *CmCHXE*, *CmCCS*, *CmLCYE*, *CmCHXB*, *CmCCD*, *CmLCYB*, *CmPDS*, *CmVDE*, *CmZ-ISO*, *CmZEP*, *CmAO*, and *CmNCED*. *CmZDS* also had the maximum signal peptide (S score) values, followed by *CmCCS*, *CmCHXE*, *CmCrtISO*, *CmPSY*, *CmLCYE*, *CmZ-ISO*, *CmCHXB*, *CmCCD*, *CmPDS*, *CmLCYB*, *CmVDE*, *CmZEP*, *CmNCED*, and *CmAO* (Table 2).

The homology analysis using CDD showed that the CBP, XBP, and ABP sequences had high similarity with other higher plant species, including the amino acids position 21–278 for *CmPSY*, 38–583 for *CmPDS*, 157–244 for *CmZ-ISO*, 1–581 for *CmZDS*, 41–580 for *CmCrtISO*, 61–503 for *CmLCYB*, 4–534 for *CmLCYE*, 1–314 for *CmCHXB*, 61–293 for *CmCHXE*, 1–647 for *CmZEP*, 83–484 for *CmVDE*, 85–129 for *CmCCS*, 46–609 for *CmCCD*, 66–183 for *CmNCED*, and 12–1371 for *CmAO*. The homology analysis of the CBP, XBP, and ABP genes of *Dunaliella salina* by CCD search showed a high homology with other chlorophyta and higher plants [64,66,67]. These results showed that the CBP, XBP, and ABP genes of *C. majus* are highly conserved compared with the genes of higher plants and green algae.

### 2.2. Phylogenetic and Homology Analysis

The phylogenetic tree between *C. majus* and other CBP, XBP, and ABP genes was constructed using a neighbor-joining method. The results showed that the CBP, XBP, and ABP protein sequences of *C. majus* were grouped with other higher plants, whereas chlorophyte, bacteria, dinoflagellates, and heterokonts were grouped in a separate cluster (Figure S2). Several studies have reported that the phylogenetic analysis of CBP, XBP, and ABP genes in plants showed a close relationship with other higher plants, whereas other species formed separate clades [35,40,52,57]. Similarly, the identity matrix results showed that all CBP, XBP, and ABP genes shared sequence identities with the amino acid sequences of other higher plants, such as *Adonis aestivalis*, *Camellia sinensis*, *Macleaya cordata*, and *Papaver somniferum*. In addition, other species showed less sequence identity when compared with *C. majus*’ CBP, XBP, and ABP amino acid sequences (Table 2). Similar results were obtained in *S. baicalensis* and *I. dentate*, where their CBP, XBP, and ABP amino acid sequences showed high similarity with higher plant species [29,57,59]. These results clearly showed that the CBP, XBP, and ABP genes of higher plants are highly conserved, which may share higher sequence identities with higher plants.

### 2.3. Multiple Alignments and Tertiary Structure Analysis of CBP, XBP, and ABP Genes

**Figure 2:**
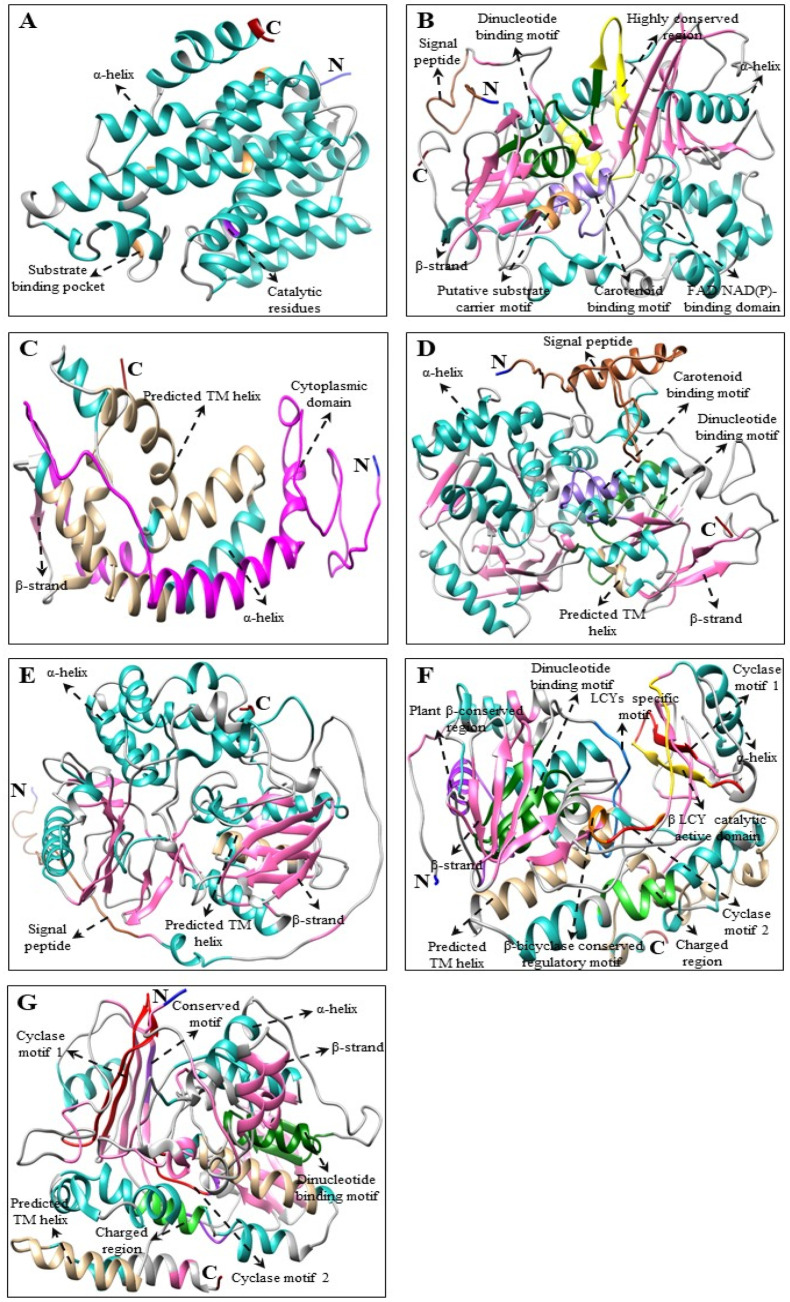
Three-dimensional structure of CBP genes of *C. majus*. (**A**) *CmPSY*, (**B**) *CmPDS*, (**C**) *CmZ-ISO* (partial ORF), (**D**) *CmZDS*, (**E**) *CmCrtISO*, (**F**) *CmLCYB*, and (**G**) *CmLCYE* structures were produced by using Chimera 1.14 software [76]. The amino (NH_2_) and carboxyl (COOH) termini are shown in blue and dark red, respectively. In these 3D structures, α-helices are shown in light sea green whereas β-strands are shown in hot pink. Multiple alignments of each gene are shown in Figure S3.

**Figure 3:**
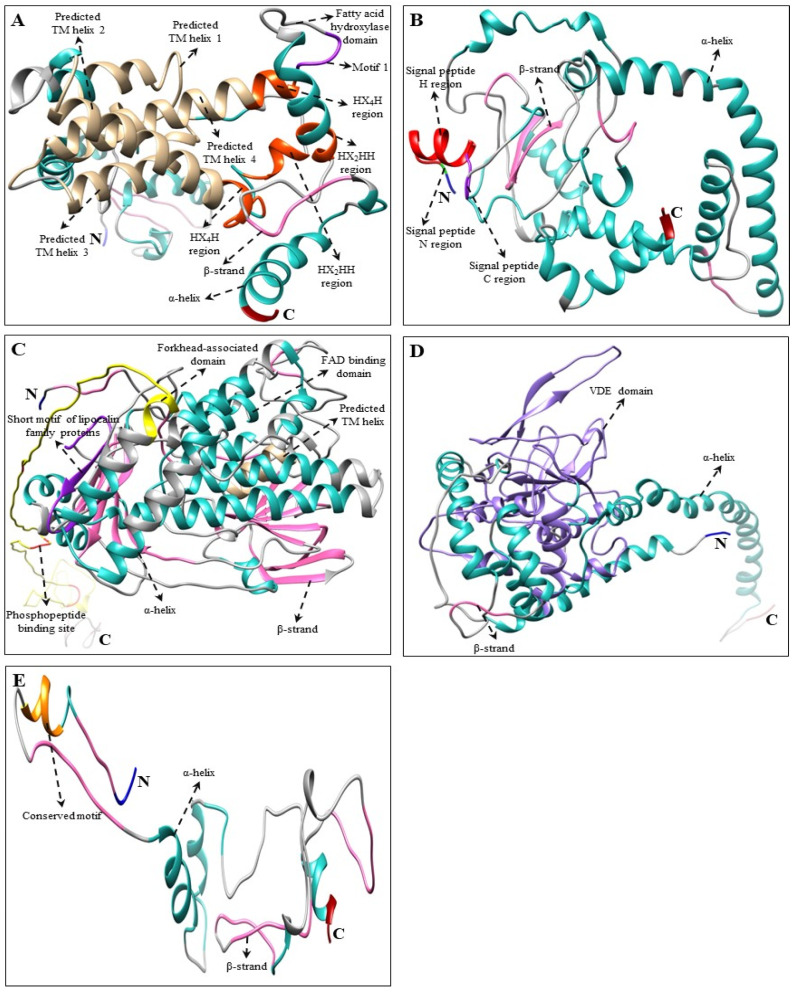
Three-dimensional structure of XBP genes of *C. majus*. (**A**) *CmCHXB*, (**B**) *CmCHXE* (partial ORF), (**C**) *CmZEP*, (**D**) *CmVDE*, and (**E**) *CmCCS* (partial ORF) structures were produced by using Chimera 1.14 software [76]. The amino (NH_2_) and carboxyl (COOH) termini are shown in blue and dark red, respectively. In these 3D structures, α-helices are shown in light sea green whereas β-strands are sown in hot pink. Multiple alignments of each gene are shown in Figure S3.

**Figure 4:**
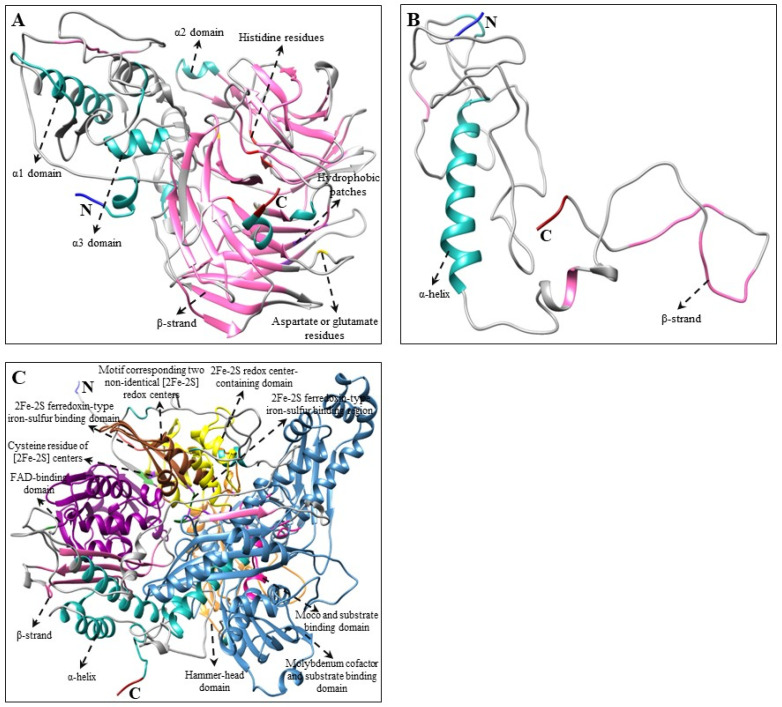
Three-dimensional structure of ABP genes of *C. majus*. (**A**) *CmCCD*, (**B**) *CmNCED* (partial ORF), and (**C**) *CmAO* structures were produced by using Chimera 1.14 software [76]. The amino (NH_2_) and carboxyl (COOH) termini are shown in blue and dark red, respectively. In these 3D structures, α-helices are shown in light sea green whereas β-strands are shown in hot pink. Multiple alignments of each gene are shown in Figure S3.

Multiple alignments and predicted tertiary structures of *C. majus* CBP, XBP, and ABP proteins showed highly conserved domains as that of the higher plants [29,50,51,52,54] and microalgae [66,68,69,70] (Figure S3 and Figure 2, Figure 3 and Figure 4). Although there were few base changes in amino acid sequences, the protein function mainly depends on its tertiary structure and stability [71]. The tertiary structures of *C. majus*’ CBP, XBP, and ABP protein sequences showed similar conformations of α and β secondary structural elements and substrate-binding pockets as that of *A. thaliana*, *N. officinale*, *C. reinhardtii*, and *D. salina* (data are not shown). However, there were little structural differences observed in the variable loop regions of the CBP, XBP, and ABP protein models, which might be due to their relatively low sequence identities [72]. These results agreed with the results of percent identities and multiple alignments in this study (Table 2 and Figure S3).

The predicted tertiary structure of *C. majus*’ CBP, XBP, and ABP genes consist of a central hydrophobic substrate-binding pocket, which was created by the folding of α-helices and β-sheet strand; the binding pocket was almost buried within the core of α-helices. In addition, other domains including the carotene-binding domain (CBD), aspartate-rich domain (ARD), and dinucleotide-binding domain (DBD), were located near the cavity, which might be important for enzyme activity [66]. In detail, the key CBP pathway enzyme *CmPSY* consists of a conserved trans-isoprenyl diphosphate synthase domain and ARD in its amino acid sequence (Figure 2 and Figure S3). Similar conserved domains were present in the amino acid sequences of higher plants, such as *S. baicalensis* and *I. dentate* [29,31]. The second most important gene in this pathway is *CmPDS*, which consists of both DBD and CBD in its sequence, whereas *CmZDS* consists of similar identical features as that of *CmPDS*, which possesses a CBD and DBD at the C-terminal region and N-terminal region, respectively. This result was similar to a previous study which showed that the PDS and ZDS amino acid sequence of higher plants (*I. dentate*, *Carica papaya*, and *S. baicalensis*) and marine green algae (*D. salina*) have these domains [29,31,70,73]. Both *CmLCYB* and *CmLCYE* possess a DBD in their structure (Figure 2 and Figure S3). This domain is present in all lycopene cyclases, which help in binding flavin adenine dinucleotides (FAD). In addition, a plant β-conserved region was also present in plant-type cyclases (CrtL) but not in bacterial CrtYm, and this β-conserved region might play a specific role in an interaction between the cyclases and components related to membrane-bound enzymes [74]. Moreover, another three conserved regions (cyclase motif 1, 2, and charged regions), were also found in lycopene cyclases, which are involved in substrate binding and catalysis [43]. These conserved domains were also present in higher plants (*Arabidopsis* and *Capsicum annuum*) and green algae (*Haematococcus pluvialis*) [43,74,75].

The common genes responsible for both CBP and XBP are *CmCHXB* and *CmCHXE*. *CmCHXB* consists of four histidine domains that might be helpful for the attachment of Fe^2+^ ion during hydroxylation [29,77]. In cucumber, the VDE gene consists of Cys-rich, lipocalin, and Glu-rich domains in their structure [52]. The lipocalin domain is the binding site for the hydrophobic V [78]. The C- and N- terminal regions consist of a high number of Glu residues and proteins targeted to chloroplasts, respectively [78,79]. A similar protein structure was found in *CmVDE* protein sequences, which consist of these conserved domains (Figure 3 and Figure S3). *CmZEP* consists of a forkhead-associated (FHA) binding domain, two short motifs of lipocalin family proteins, and a FAD-binding domain in its structure. In addition, it has various phosphopeptide binding sites in its amino acid sequence. Similar domains and phosphopeptide binding sites were present in the amino acid sequences of higher plants, such as *I. dentate* and *S. baicalensis* [29,31]. In tiger lily (*Lilium lancifolim*), the FLEET motif was identified, which is essential for the β- and κ-cyclase activities of *LlCCS* [53,80,81]. A similar FLEET motif was found in the amino acid sequence of *CmCCS* (Figure 3 and Figure S3).

In XBP genes, *CmCCD* and *CmNCED* have four highly conserved histidine residues (Figure 4 and Figure S3), similar to the structures of *CCD4a*, *b1*, and *c* genes in *Citrus* plants [56]. Several studies have reported that these four histidine residues are helpful in coordinating the Fe^2+^ cofactor essential for activity and glutamate or aspartate moieties that help to fix positions of the histidine [55,82,83]. In *Pisum sativum*, the *PsAO* gene possesses a consensus sequence for two iron–sulfur centers, molybdenum cofactor (Moco) binding domain, and FAD-binding domain [84]. Similar conserved domains were displayed in the amino acid sequence of *CmAO* (Figure 4 and Figure S3). The alignment and structural analysis showed that most of the *C. majus*’ CBP, XBP, and ABP genes are highly conserved; and that the genes are most closely related to those of higher plants and algae. However, further comprehensive studies are required to recognize the functions of *C. majus*’ CBP, XBP, and ABP proteins identified in this study.

### 2.4. Subcellular Location Prediction of CBP, XBP, and ABP Genes by In Silico Analysis

*C. majus*’ CBP, XBP, and ABP sequences were analyzed using CELLO2GO, WoLF PSORT, TargetP 1.1, ChloroP 1.1, and Plant-PLoc free online programs to determine the subcellular location of these proteins. Most CBP, XBP, and ABP proteins, except CmPSY, were targeted to the chloroplast, whereas some proteins might also be targeted to various organelles, such as cytoplasm, endoplasmic reticulum, mitochondrion, nucleus, plasma membrane, and thylakoid membrane (Table 3). Similar results were obtained in several plants, such as *A. thaliana*, transgenic *Ipomoea batatas*, where most CBP, XBP, and ABP genes were localized within the chloroplast [67,85,86]. Therefore, we confirmed that all CBP, XBP, and ABP proteins in *C. majus* share highly conserved sequences with those in higher plants, so their subcellular location prediction also showed similar results.

### 2.5. CBP, XBP, and ABP Gene Expression Levels in Different Parts of C. majus

**Figure 5:**
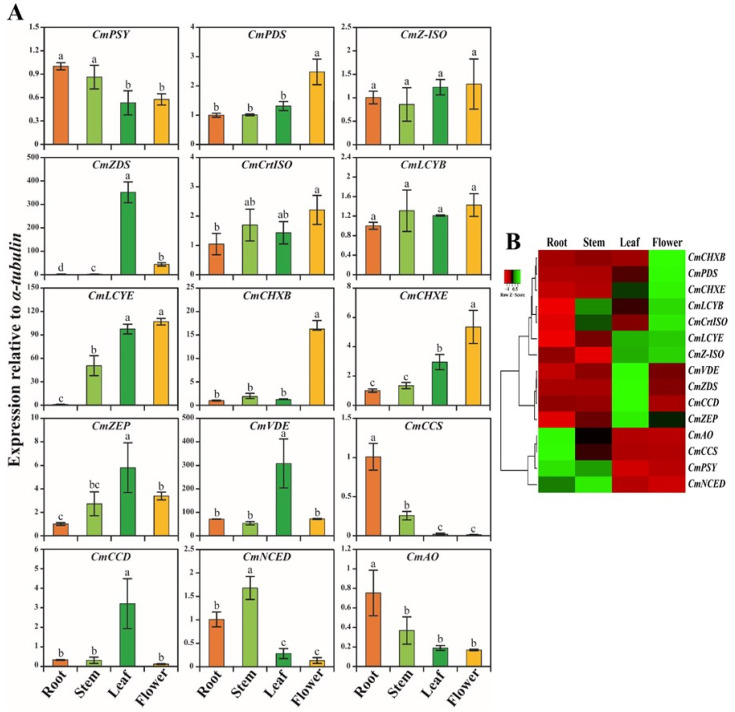
Relative gene expression profiles of eleven CBP, XBP, and ABP genes of *Chelidonium majus*. (**A**) Expression levels of CBP, XBP, and ABP genes were examined in different organs such as leaf, stem, root, and flower of *Chelidonium majus* using qRT-PCR. Letters a–e denotes significant differences (*p* < 0.05). (**B**) Heat map showing the expression profiles of CBP, XBP, and ABP genes in five different organs of *Chelidonium majus*. The heat map was constructed using fold-change values gained from qRT-PCR. The tree view of hierarchical clustering was used to display the organ-specific expression of CBP, XBP, and ABP genes. A gradient color bar at the top is used to show whether the CBP, XBP, and ABP genes are up-regulated (red) or down-regulated (green).

The qRT-PCR results showed that the CBP, XBP, and ABP genes were integrally expressed in *C. majus*. Among these, the highest expression level was observed in *CmZDS*, whereas the lowest expression level was found in *CmAO* (Figure 5). In the CBP genes, most genes (*CmPDS*, *CmZ-ISO*, *CmCrtISO*, *CmLCYB*, and *CmLCYE*) were highly expressed in flowers. *CmZDS* was significantly higher in leaves, which was 7.97-, 145.11-, and 216.08-times higher than those in flowers, stems, and roots, respectively. In contrast, *CmPSY* had the highest expression in roots, followed by that in stems, flowers, and leaves. In addition, most XBP genes (*CmCHXB* and *CmCHXE*) were strongly upregulated in flowers, whereas *CmZEP* and *CmVDE* were significantly upregulated in leaves. The expression of *CmVDE* was the highest in leaves, which was 4.30-, 5.78-, and 288.80- times higher than that in flowers, stems, and roots, respectively. However, *CmCCS* had a higher expression level in roots when compared with the other parts. In ABP genes, *CmCCD* was significantly expressed in leaves, which was 9.74-, 10.26-, and 26.69- times higher than that in roots, stems, and flowers, respectively, whereas *CmNCED* and *CmAO* had an increased expression in stems, and roots, respectively (Figure 5).

A similar result was obtained in most studies, where most CBP genes were highly expressed in the flowers and leaves of plants, such as *Brassica rapa* [87] and *N. officinale* (unpublished data), when compared with the other plant parts. In this study, the expression of CBP genes showed that most genes had the same role as their orthologs in other species. For example, [88] stated that the genes responsible for CBP (*AtPSY*, *AtPDS*, *AtZDS*, and *AtZEP*) play a vital role. Overall, the results of this study showed that most CBP, XBP, and ABP genes had the highest expression in flowers (*CmPDS*, *CmZ-ISO*, *CmCrtISO*, *CmLCYE*, *CmLCYB*, *CmCHXB*, and *CmCHXE*), followed by that in leaves (*CmZDS*, *CmZEP*, *CmVDE*, and *CmCCD*), root (*CmPSY*, *CmAO*, and *CmCCS*) and stems (*CmNCED*). This expression analysis of CBP genes will contribute to future genetic studies on in *C. majus* to enhance their carotenoid content through metabolic engineering.

### 2.6. Carotenoid and Xanthophyll Level in Different Parts of C. majus

**Figure 6:**
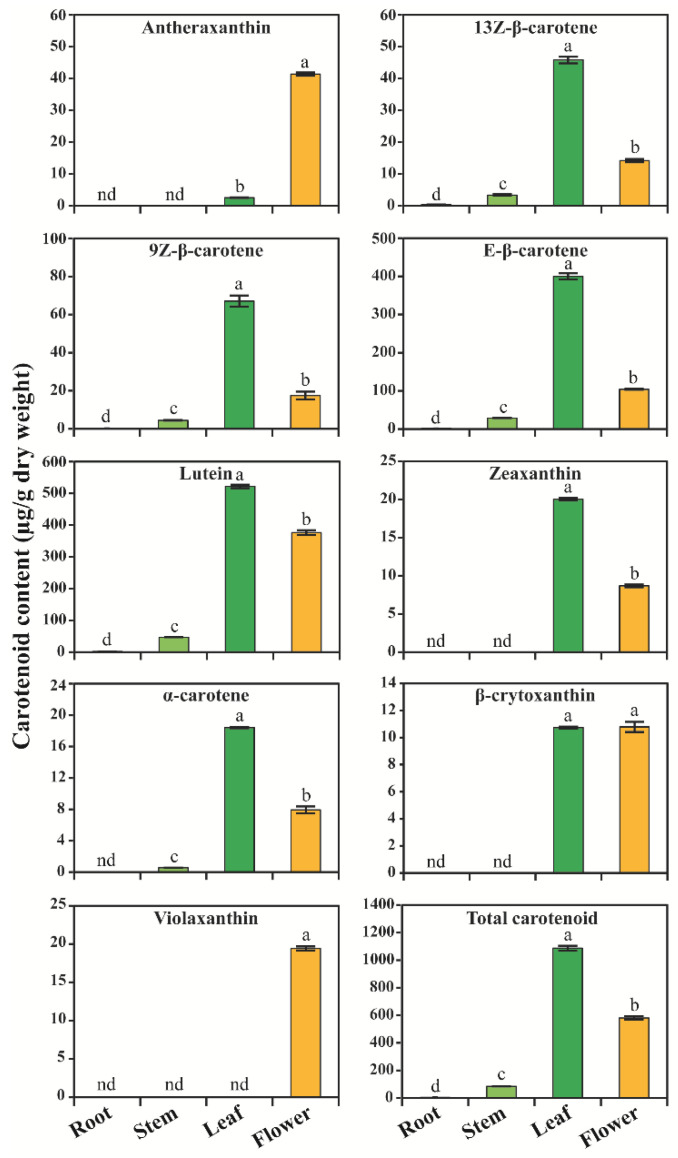
Carotenoid content in the different organs of *Chelidonium majus*. For HPLC analysis, samples were harvested from 3-month-old plants. Results are given as the means of triplicates ± standard deviation. Letters a–e denotes significant differences (*p* < 0.05). nd: not detected.

Carotenoid levels were analyzed using HPLC. Eight different types of carotenoids were identified in different parts of *C. majus* (Figure 6). The total carotenoid content ranged from 4.667–1086.43 μg/g of dry weight (DW) in different parts of *C. majus*. The leaves showed the highest total carotenoid content (1086.43 μg/g DW), which was 1.87-, 12.87-, and 232.79- times higher than that in flowers, stems, and roots, respectively. Among the nine carotenoids, six carotenoids namely lutein, zeaxanthin, 13Z-β-carotene, α-carotene, E-β-carotene, and 9Z-β-carotene, had the highest accumulation in leaves (Figure 6). Specifically, lutein, zeaxanthin, 13Z-β-carotene, α-carotene, E-β-carotene, and 9Z-β-carotene were significantly higher in leaves than in other plant parts.

Among the carotenoids, lutein, 13Z-β-carotene, E-β-carotene, and 9Z-β-carotene were detected in all plant organs. Among these, the lutein content was higher in leaves, which was 1.39-, 11.03-, and 236.23- times higher than that in flowers, stems, and roots, respectively, whereas the E-β-carotene level in leaves was 3.84-, 13.90-, and 220.14- times higher than that in flowers, stems, and roots, respectively. Similarly, the 9Z-β-carotene level was the highest in leaves, and it was 3.84-, 15.05-, and 240.51- times higher than that in flowers, stems, and roots, respectively. Zeaxanthin and β-crytoxanthin were detected only in flowers and leaves. Violaxanthin was detected only in flowers, whereas the neoxanthin content was not detected in any of the plant organs. The α-carotene content (μg/g DW) was the highest in leaves (18.41), followed by that in flowers (7.93) and stems (0.56), and it was not present in roots. Interestingly, the antheraxanthin content was significantly higher in flowers than in leaves, and it was not detected in roots and stems. Among the individual carotenoids, antheraxanthin, β-crytoxanthin, α-carotene, and violaxanthin had the lowest content in the different parts of *C. majus* (Figure 6). These findings were similar to those in previous studies on *M. charantia* [89,90,91], *B. rapa* [92], *C. majus* [9], and *Allium sativum* [90]. Similar results were obtained in *N. officinale*, as its leaves also had the highest accumulation of carotenoids when compared with other plant organs (unpublished data). Thus, the leaves of *C. majus* had the highest carotenoid content when compared with other plant organs.

This study showed that the highest transcription of CBP, XBP, and ABP genes was found in flowers (Figure 5), whereas an increased carotenoid and xanthophyll content was achieved in the leaves of *C. majus* (Figure 6). This showed that the CBP, XBP, and ABP gene expression and the pattern of carotenoid and xanthophyll accumulation were not correlated, indicating that the enhanced transcriptional expression of genes does not always lead to the highest accumulation of carotenoids [57,66]. This might be due to the regulation of CBP, XBP, and ABP at multiple levels; the pathways are not only controlled at the transcriptional level but also at the translational level [93,94]. Furthermore, the CBP, XBP, and ABP gene expressions are regulated by a group of *cis*-regulatory elements present in the upstream promoter region and untranslated regions [95,96]. In addition, protein modification might be another one of the reasons for the inequitable accumulation pattern of carotenoids and xanthophylls and CBP, XBP, and ABP gene expressions [97].

Due to the significant importance of the CBP, XBP, and ABP metabolism and function in the plant’s development, physiology, ecology, and evolution, we had thoroughly studied all those genes in these pathways. In recent years, much research has been focused on carotenoid accumulation at multiple regulatory levels such as transcriptional, post-transcriptional and translation modification, storage, degradation of carotenoids, and feedback regulation of the end products [23]. For this reason, identification of CBP, XBP, and ABP genes from the transcriptomic data, characterization of those genes by sophisticated bioinformatics approach and analytical tools, and also understanding the expression level of those genes will help to uncover the relationship between metabolomics and transcriptomics profiles [23,98,99,100,101]. Identifying the complete pathway and 3D structure of CBP, XBP, and ABP genes will be helpful to manipulate the gene, engineer the potential genes, and transform the multiple CBP, XBP, and ABP genes instantaneously into the host plants for the improvement of carotenoid biosynthesis and enhance the novel or desired carotenoid products in stable crops [102,103]. In addition, subcellular localization analysis of the whole CBP, XBP, and ABP will help to achieve a deep understanding of the assembly of each gene in different organelles of the plants [103].

## 3. Materials and Methods

### 3.1. Plant Materials

*Chelidonium majus* seeds were acquired from an experimental farm of Chungnam National University, Daejeon, Republic of Korea. The seeds were placed in a pot filled with commercial perlite and allowed to grow for three months in the greenhouse of Chungnam National University (Daejeon, Korea). The plants were sprayed with water every two days. The different plant organs (leaves, stems, roots, and flowers) were harvested, flash-frozen in liquid nitrogen, and stored at −80 °C until further analysis. Each sample was collected in triplicates.

### 3.2. Identification and Sequence Analysis of CBP, XBP, and ABP Genes

CBP, XBP, and ABP gene sequences were identified from the *C. majus* transcriptomic data (62 Mb raw reads, average length of 76 nucleotides per reads) obtained in our laboratory. An Illumina NextSeq500 platform was used to analyze the cDNA using the commercial service of LAS company (Gimpo, Korea). Then, the retrieved CBP, XBP, and ABP sequences were subjected to an online Basic Local Alignment Search Tool (BLAST) on an NCBI database. The sequences were also analyzed using PFAM [104] and Conserved Domain Database (CCD) [105] on the NCBI databases to predict the putative signature motifs of the protein sequences. Secondary structure and signal peptide analyses were conducted using a SOPMA program [106] and SignalP 4.0 server [107], respectively. The predicted subcellular locations of the CBP proteins were identified using CELLO [108], ChloroP 1.1 [109], TargetP 1.1 [110], and WoLF PSORT [111] tools. Then, the theoretical pI (isoelectric point)/molecular weight (MW) was calculated by using the Compute pI/MW tool on an ExPASy platform [112].

### 3.3. Structural Analysis of CBP, XBP, and ABP Genes

Multiple sequence alignment was carried out using a BioEdit 7.2.5 program [113]. The CBP, XBP, and ABP protein sequences were submitted to Phyre2 for homology modeling and three-dimensional (3D) structural analysis [114]. Then, 3D structures were predicted using a Chimera 1.14 software [76]. The conserved signature motifs among the CBP, XBP, and ABP genes were found using a MEME tool [115].

### 3.4. Phylogenetic Analysis and Percent Identity Matrix

A phylogenetic tree was constructed using MEGA 7.0 [116]. Neighbor-joining (NJ) phylogenetic trees [117] were constructed using a Poisson model. The robustness of the trees was estimated by performing 1000 bootstrap replicates [118]. The percent identity matrix between the CBP, XBP, and ABP amino acid sequences was calculated using clustal omega [119], and identities were calculated from the pairwise multiple sequence alignment [120].

### 3.5. RNA Extraction and cDNA Synthesis

Total RNA extraction was conducted on the leaves, stems, roots, and flowers of the plants. Each sample was ground into a fine powder using a mortar and pestle with the help of liquid nitrogen. Then, 0.1 g of each sample was transferred to a new 1.5-mL microcentrifuge tube. A Plant Total RNA Mini Kit (Geneaid, Taiwan) was used to extract the total RNA, following the manufacturer’s protocols. The RNA quality and concentration were determined using 1% agarose gel electrophoresis and NanoVue Plus spectrophotometer (GE Health Care Life Sciences, Chicago, IL, USA), respectively. The extracted total RNAs were reverse transcribed into cDNA using a ReverTra Ace-α-kit (Toyobo Co. Ltd., Osaka, Japan), following the manufacturer’s protocols. The synthesized cDNA templates were diluted 20-fold with RNase-free water for further experiments.

### 3.6. CBP, XBP, and ABP Genes Expression

For qRT-PCR, *α-tubulin* gene was used as an internal control. Specific primers for the CBP, XBP, ABP, and *α-tubulin* genes were designed using a Gene Runner version 5 software (www.generunner.net, accessed on 10 July 2021). The primers used in this study are shown in Table S1. The relative gene expression level was calculated using *α-tubulin*. The qRT-PCR conditions were similar to the protocol described by Tuan et al. [31]. The gene expression level was calculated using a ΔCt method [121,122]. The visualization and expression analysis of CBP, XBP, and ABP genes in the heatmap and hierarchical clustering was conducted using an online heat mapper software [123]. All PCR reactions were carried out in triplicates.

### 3.7. Carotenoid Extractions and HPLC Analysis

Carotenoids were extracted and analyzed by following the protocol described by Park et al. [124]. For HPLC analysis, 3 mL of ethanol containing 0.1% ascorbic acid (*w*/*v*) was added to 0.3 g of finely powdered samples. This was mixed well and incubated at 85 °C for 10 min in a water bath. For saponification, 120 μL of potassium hydroxide (80% *w*/*v*) was added. To stop the reaction, the samples were flash-frozen on ice for 5 min. Then, 1.5 mL of ice-cold water and 0.05 mL of β-apo-8′-carotenal internal standard (1.25 µg) were added. Carotenoids were re-extracted thrice using 1.5 mL of hexane and were centrifuged each time at 12,000 rpm for 5 min at 4 °C. The extracts were dried under nitrogen stream and were re-dissolved in 0.25 mL of dichloromethane/methanol (50:50 *v*/*v*). These mixtures were filtered through a polytetrafluoroethylene (PTFE) membrane filter (0.50 µm, Advantec, Tokyo, Japan) into amber screw cap vials (Thermo Fisher Scientific, Waltham, MA, USA). The HPLC conditions and gradient programs were similar to a previous protocol [124]. The individual carotenoid concentrations were quantified using their retention time and co-elution with β-apo-8′-carotenal; these were quantitated based on standard calibration curves. All standards were obtained from CaroteNature (Lupsingen, Switzerland).

### 3.8. Statistical Analysis

In this study, all results are expressed as the mean ± standard deviation (SD) of three independent biological replicates. All data were analyzed by analysis of variance (ANOVA) with Duncan’s multiple range tests (DMRT) to compare the means, with a significance level of *p* < 0.05 using the Statistical Analysis System version 9.2 (SAS Institute Inc., Cary, NC, USA, 2009).

## 4. Conclusions

This is the first report to identify and characterize the CBP genes in *C. majus* using a molecular approach. Using in silico analysis, we identified and characterized seven CBP, five XBP, three ABP genes in *C. majus*, and among these, eleven genes possess a full ORF, whereas four genes had a partial ORF. By using bioinformatics tools including multiple alignments and 3D structure prediction, we showed that *C. majus* CBP gene sequences shared high similarity with other higher plants and microalgae. In addition, subcellular localization prediction showed that most of the *C. majus* CBP genes were localized in the chloroplast. Differential expression of CBP genes showed an organ-specific variation at the transcriptional level with most CBP genes highly up-regulated in the flowers. Furthermore, the highest accumulation of carotenoids was observed in *C. majus* leaves, whereas other organs showed less accumulation. This indicates that CBP, XBP, and ABP is complex and does not just change based on alterations in mRNA expression. Differences between the gene expression levels and carotenoid accumulation may depend on signals that dictate whether CBP genes can be activated in various parts of plants at different stages. This study will therefore improve our understanding of the molecular mechanisms regulating carotenoid accumulation in *C. majus*, and this can subsequently serve as a valuable resource for genetic manipulation to increase the nutritional content of *C. majus*. In future, further studies are necessary to achieve genome-wide identification of the CBP gene in *C. majus* genome which will be beneficial to identify more homologues, gene family, and alleles.

## Figures and Tables

**Table 1:** Molecular characterization of CBP genes in *C. majus.*

Gene Names	NCBI Accession No.	ORF (bp)	Length (aa)	ORF Type	MW (Kda)	pI	Signal Peptide
*CmPSY*	MW307341	912	303	Full	36.36	9.56	No
*CmPDS*	MW307330	1752	583	Full	69.96	6.81	No
*CmZ-ISO*	MW307331	734	244	Partial	29.28	6.71	No
*CmZDS*	MW307344	1797	598	Full	71.76	7.89	No
*CmCrtISO*	MW307336	1812	603	Full	72.36	8.79	No
*CmLCYB*	MW307338	1518	505	Full	60.60	7.64	No
*CmLCYE*	MW307339	1605	534	Full	64.08	6.49	No
*CmCHXB*	MW307333	948	317	Full	38.04	8.75	No
*CmCHXE*	MW307335	881	293	Partial	35.16	9.12	No
*CmZEP*	MW307343	1998	665	Full	79.80	6.59	No
*CmVDE*	MW307342	1464	487	Full	58.44	5.13	No
*CmCCS*	MW307334	389	129	Partial	15.48	10.22	No
*CmCCD*	MW307337	1836	611	Full	73.32	6.23	No
*CmNCED*	MW307340	549	183	Partial	21.96	10.40	No
*CmAO*	MW307332	4125	1374	Full	164.88	6.34	No

**Table 2:** Percentage identity (%) analysis of amino acid sequences, comparing between *C. majus* CBP, XBP, and ABP genes and other CBP, XBP, and ABP amino acid sequences. Accession numbers of the sequences are provided in Figure S2 and Table S3.

Species Name	*PSY*	*PDS*	*Z-ISO*	*ZDS*	*CrtISO*	*LCYB*	*LCYE*	*CHXB*	*CHXE*	*ZEP*	*VDE*	*CCS*	*CCD*	*NCED*	*AO*
Higher plants															
*C. majus*	100	100	100	100	100	100	100	100	100	100	100	100	100	100	100
*A. aestivalis*								72.46							
*C. sinensis*		82.30				85.32	77.50								71.55
*M. cordata*	89.77														
*P. somniferum*			70.26	78.00	81.40				81.60	82.56	76.39	70.65	79.53	68.05	
Chlorophyta															
*C. primus*			55.63			51.23	41.49		48.95		40.41				
*C. reinhardtii*		63.60		56.36	55.93			44.96		49.08			30.13		30.72
*M. pusilla*														27.78%	
*O. tauri*	38.70														
Bacteria															
*Calothrix* sp.													36.36		
*D. bacterium*						35.66		44.03							32.86
*Geitlerinema* sp.		66.03					35.82								
*O. cyanobacterium*				61.73	59.92										
*R. bacterium*	35.00														
*Spirulina* sp.														46.00	
Dinoflagellates															
*S. microadriaticum*		40.50			25.27					33.20	35.62		25.44		27.39
Heterokonts															
*E. siliculosus*				49.65	28.23	35.78				32.15	40.44		23.63		
*F. cylindrus*	27.24		29.06												31.00
*H. fermentalgiana*								33.16							
*S. lomentaria*		59.75													

**Table 3:** The subcellular-localization predictions of *C. majus* CBP, XBP, and ABP genes.

Gene Names	CELLO2GO	WoLF PSORT	TargetP	ChloroP 1.1	Plant-PLoc	Consensus Prediction
*CmPSY*	MC	MC	Other	Other	NUC	MC/NUS/other
*CmPDS*	CP	CP	CP	CP	CP	CP
*CmZ-ISO*	PM	CP	CP	CP	CP	CP/PM
*CmZDS*	CP	CP	CP	CP	CP	CP
*CmCrtISO*	CP	CP	CP	CP	CP	CP
*CmLCYB*	MC	CP	Other	Other	CP	CP/MC/other
*CmLCYE*	PM	CP	CP	CP	CP	CP/PM
*CmCHXB*	CP	CP	CP	CP	CP	CP
*CmCHXE*	CP	CP	CP	CP	CP	CP
*CmZEP*	CP	Cytoplasmic	CP	Other	CP	CP/cytoplasmic/other
*CmVDE*	Cytoplasmic	ER	Thylakoid	Other	CP	CP/ER/thylakoid/other
*CmCCS*	MC	CP/MC	CP	CP	CP	CP/MC
*CmCCD*	CP	Cytoplasmic	Other	CP	CP	CP/cytoplasmic/other
*CmNCED*	NUC	CP	Other	CP	CP	CP/NUC/other
*CmAO*	CP	Cytoplasmic	Other	Other	CP	CP/cytoplasmic/other

Note: CP—chloroplast; ER—endoplasmic reticulum; MC—mitochondria; NUC—nucleus; PM—plasma membrane.

## Data Availability

Data reported are available in the Supplementary Materials.

## Supplementary Materials

The following are available online at https://www.mdpi.com/article/10.3390/plants10081753/s1, Figure S1: (A–O): The nucleotide sequence and deduced amino acid sequences of CBP, XBP, and ABP genes. Figure S2: (A–O): Phylogeny and conserved motif analysis of deduced CBP, XBP, and ABP amino acid sequences along with other CBP sequences. Figure S3: (A–O): Amino acid alignment of CBP, XBP, and ABP genes with other CBP genes. Table S1: List of primers used in qRT-PCR analysis to determine mRNA expression levels of *C. majus* CBP, XBP, and ABP genes. Table S2: Analysis of CBP, XBP, and ABP gene sequences using the SignalP program.
