# Supplementary material for: Molecular Characterization, Expression Analysis of Carotenoid, Xanthophyll, Apocarotenoid Pathway Genes, and Carotenoid and Xanthophyll Accumulation in Chelidonium majus L
Source: Plants (Basel). 2021 Aug 23;10(8):1753. doi: 10.3390/plants10081753 (PMC8398043; doi:10.3390/plants10081753)
Supplement: Supplementary file 1 [file plants-10-01753-s001.zip › plants-1341285-supplementary.pdf]

```
-102 CTCTAGATCTGTTGAAATTGAAGCAAGGATCGATCAGAATTCAGAAAGTAGTGCAACAA
     GCTCGAGCTCGACTCCTAAGATCTCCCGTAGTATACTACGAAAGAACCACATAGAATTTG
   1 ATGAATGGCGCTTCAACATCTAGTACCATCCGAGCAGCCTTCTCCCACTGTGTACAACAA
      M  N  G  A  S  T  S  S  T  I  R  A  A  F  S  H  C  V  Q  Q
  61 GTACGAAGCTATGATTACCACCATTACCTTTGCCTCCTCCAGCTGTCCCCTAACATGCGG
      V  R  S  Y  D  Y  H  H  Y  L  C  L  L  Q  L  S  P  N  M  R
 121 AAAGCTGCATTTGCTCTGCGTGCTTTCAATGTAGAAACTGCCAGAGCCATGGATGTTGCT
      K  A  A  F  A  L  R  A  F  N  V  E  T  A  R  A  M  D  V  A
 181 TCTGATCCAAGAATTGGTCTTATGCGCCTCCTTTGGTGGCAAGAAGCCATAGACAAAATC
      S  D  P  R  I  G  L  M  R  L  L  W  W  Q  E  A  I  D  K  I
 241 TTCGCCCACAAGAAAATCGAACATCCCACTGCTCAAGCTCTCTCTTCGATAATCTCTGAA
      F  A  H  K  K  I  E  H  P  T  A  Q  A  L  S  S  I  I  S  E
 301 CACAAAATCAGCAAAAGTTGGCTAAAGAGGTCTGTTGAAGCTCGGATTATCGATGCAAGA
      H  K  I  S  K  S  W  L  K  R  S  V  E  A  R  I  I  D  A  R
 361 AGGGAGGAAAATGAAATCCCACAGTCGATCGAAGAATTGGAAAAATATGCCGAATCCACT
      R  E  E  N  E  I  P  Q  S  I  E  E  L  E  K  Y  A  E  S  T
 421 GTTTCCACCATATTATATATGACACTTCAAGCTGGAGGAATAAGGTCCACGGCAGCGGAT
      V  S  T  I  L  Y  M  T  L  Q  A  G  G  I  R  S  T  A  A  D
 481 CATGCAGCTTCACACATTGGGAAGGCAAGCGGTCTCCTTTTGCTCCTTAGATCATTACCT
      H  A  A  S  H  I  G  K  A  S  G  L  L  L  L  L  R  S  L  P
 541 TACCACCATAGTCGTAATTCCCAACTTCGCTATATACCATTTGAAGTGGCATCCAAGCAC
      Y  H  H  S  R  N  S  Q  L  R  Y  I  P  F  E  V  A  S  K  H
 601 GGATTGTTAAGTCACGAGGGAGGTCAAAATCAAGTTCAGCTGGGTTACCGTGAGGGTTTA
      G  L  L  S  H  E  G  G  Q  N  Q  V  Q  L  G  Y  R  E  G  L
 661 CCAAATGCAGTATTTGAGATAGCTTCCGTTGCTAGTGTTCATTTGCAGAAGGCTCGTGAG
      P  N  A  V  F  E  I  A  S  V  A  S  V  H  L  Q  K  A  R  E
 721 TTGGCAAAGACTGTGCCTGCGGAGGCTCTTCCCGTGTTGCTTCCAGCTGTGCCTGCACAG
      L  A  K  T  V  P  A  E  A  L  P  V  L  L  P  A  V  P  A  Q
 781 GTTATATTGGACTCTCTACGAAGAGTGCATTTTGACGTGTTTGATCCAAGGTTGATGAGA
      V  I  L  D  S  L  R  R  V  H  F  D  V  F  D  P  R  L  M  R
 841 GGGATTCTGGGTATTTCACCCTTATGGTTTCAATTAAAGCTGAAGTGGAATGCATGGAAA
      G  I  L  G  I  S  P  L  W  F  Q  L  K  L  K  W  N  A  W  K
 901 AGTACATATTGA +1
      S  T  Y  *
```

**Figure S1A.** The nucleotide sequence and deduced amino acid sequences of CmPSY. An asterisk (*) represents a stop codon.

```
-180 CGGTGGGGAACAGATTTCACTACGCTAAAAATTTCCATTCTCTAGCTCTGTTGTGGGTCA
     TCATAGTGGGTTTTAGTAATTCCCATTGTAAAACTAGGTTGGTGACTGAAACTGGTGGAG
   1 ATGACTTTAACTAGTTCTGTTTCCGTGGTAAATTTGAGTGGACAAGGGAATATAATAAAC
      M  T  L  T  S  S  V  S  V  V  N  L  S  G  Q  G  N  I  I  N
  61 ATCTGGAACTCAAATTCCAGCAGAAGATGTTGTTTCTCTATAAATTCTGGAGATAACAAT
      I  W  N  S  N  S  S  R  R  C  C  F  S  I  N  S  G  D  N  N
 121 CTATTAGCATTTGGAGGGAGTGATTCTATGGGTCTCTCCTTGAAATCTCGAAAGGCACAT
      L  L  A  F  G  G  S  D  S  M  G  L  S  L  K  S  R  K  A  H
 181 GCTGTTGGAACTCGGCCAAGGAGGAAAGTTGGCCCTTTGCAGGTTGTTTGTGTGGACTAC
      A  V  G  T  R  P  R  R  K  V  G  P  L  Q  V  V  C  V  D  Y
 241 CCAAGACCTGAACTTGACAATACCGTTAATTTCATAGAAGCTGCTCACTTTTCTTCACTC
      P  R  P  E  L  D  N  T  V  N  F  I  E  A  A  H  F  S  S  L
 301 TTCCGTACTTCTGCTCGTCCAGAAAAACCATTGCAAGTTGTAATTGCTGGTGCAGGCTTG
      F  R  T  S  A  R  P  E  K  P  L  Q  V  V  I  A  G  A  G  L
 361 GCTGGTCTATCTACGGCAAAATATCTGGCAGATGCAGGTCACAAACCCATATTGCTGGAA
      A  G  L  S  T  A  K  Y  L  A  D  A  G  H  K  P  I  L  L  E
 421 GCAAGAGATGTTTTGGGTGGAAAGGTGGCTGCGTGGAAGGATGATGATGGAGACTGGTAC
      A  R  D  V  L  G  G  K  V  A  A  W  K  D  D  D  G  D  W  Y
 481 GAGACTGGCCTACACATATTTTTTGGAGCTTACCCGAACGTGCAGAATTTGTTTGGAGAA
      E  T  G  L  H  I  F  F  G  A  Y  P  N  V  Q  N  L  F  G  E
 541 CTTGGTATTAATGATCGATTGCAGTGGAAGGAACACTCAATGATTTTTGCCATGCCAAGC
      L  G  I  N  D  R  L  Q  W  K  E  H  S  M  I  F  A  M  P  S
 601 AAGCCAGGAGAGTTCAGCAGATTTGATTTCCCTGATGCTCTTCCTGCACCGTTAAATGGG
      K  P  G  E  F  S  R  F  D  F  P  D  A  L  P  A  P  L  N  G
 661 ATATTTGCCATCTTAAGAAACAATGAAATGCTTACATGGCCGGAGAAAGTACGGTTTGCC
      I  F  A  I  L  R  N  N  E  M  L  T  W  P  E  K  V  R  F  A
 721 ATTGGACTCTTGCCTGCGATGGTTGGTGGACAAGCTTATGTGGAGGCTCAAGATGGATTT
      I  G  L  L  P  A  M  V  G  G  Q  A  Y  V  E  A  Q  D  G  F
 781 ACCGTTAAAGATTGGATGAGAAAGCAGGGTGTTCCAGATCGAGTAACTGATGAGGTGTTT
      T  V  K  D  W  M  R  K  Q  G  V  P  D  R  V  T  D  E  V  F
 841 ATCGCCATGTCAAAGGCACTAAACTTCATAAATCCAGATGAGCTTTCGATGCAATGCATT
      I  A  M  S  K  A  L  N  F  I  N  P  D  E  L  S  M  Q  C  I
 901 TTGATTGCTTTGAATCGATTTCTTCAGGAAAAACATGGTTCTAAGATGGCCTTTTTAGAT
      L  I  A  L  N  R  F  L  Q  E  K  H  G  S  K  M  A  F  L  D
 961 GGTAACCCTCCCGAGAGACTTTGCATGCCGATCGTTGAACATATCCAATCTCGAGGTGGC
      G  N  P  P  E  R  L  C  M  P  I  V  E  H  I  Q  S  R  G  G
1021 CAAGTCGAACTCAATTCACGAATTCAAAAGATCGAGCTGAAAAGTGATGGAACTGTGAAG
      Q  V  E  L  N  S  R  I  Q  K  I  E  L  K  S  D  G  T  V  K
1081 CGTCTTATACTCACTAATGGCAATGCAATAGAAGCAGATGCTTATGTAATTGCAACTCCA
      R  L  I  L  T  N  G  N  A  I  E  A  D  A  Y  V  I  A  T  P
1141 GTTGATATCTTGAAGCTTCTTTTACCCGAAGACTGGAAAGAGACTCCCTACTTCAAGAAA
      V  D  I  L  K  L  L  L  P  E  D  W  K  E  T  P  Y  F  K  K
1201 TTGGACAAATTAGTCGGAGTTCCTGTGATTAATGTGCATATATGGTTTGACAGGAAACTG
      L  D  K  L  V  G  V  P  V  I  N  V  H  I  W  F  D  R  K  L
1261 AAAAACACATATGATCATCTACTCTTCAGCAGAAGTCCCCTCTTGAGTGTATATGCCGAC
      K  N  T  Y  D  H  L  L  F  S  R  S  P  L  L  S  V  Y  A  D
1321 ATGTCTGTAACATGTAAGGAATATTACGACCCAAACCGCTCCATGCTTGAGTTGGTTTTT
      M  S  V  T  C  K  E  Y  Y  D  P  N  R  S  M  L  E  L  V  F
1381 GCACCTGCTGAAGACTGGATATCATGCAGTGACGCAGAAATTATTGAAGCTACTTTGAAG
      A  P  A  E  D  W  I  S  C  S  D  A  E  I  I  E  A  T  L  K
1441 GAGCTCGCGAAACTCTTTCCTGATGAAATTGCTGCAGATCAGAGCAAAGCAAAAATATTG
      E  L  A  K  L  F  P  D  E  I  A  A  D  Q  S  K  A  K  I  L
1501 AAGTACCACATTGTCAAAACACCACGTTCTGTTTATAAAACCATCCCTGATTGCGAACCA
      K  Y  H  I  V  K  T  P  R  S  V  Y  K  T  I  P  D  C  E  P
1561 TGCCGTCCACTGCAAAGATCTCCTGTAGAGGGATTTTATTTGGCTGGTGACTACACAAAG
      C  R  P  L  Q  R  S  P  V  E  G  F  Y  L  A  G  D  Y  T  K
1621 CAAAAGTATTTGGCTTCAATGGAAGGTGCAGTTTTGTCTGGGAAGCTTTGTGCGCAGGCT
      Q  K  Y  L  A  S  M  E  G  A  V  L  S  G  K  L  C  A  Q  A
1681 ATTGTGCAGGACTATGAGTTGCTTGCTGCGCGGAGAGAAAAAAACAGGACGACAGAGGCA
      I  V  Q  D  Y  E  L  L  A  A  R  R  E  K  N  R  T  T  E  A
1741 AGCATCGCTTAAATAAATTATCAGATTTGTGCATTTCTGTATTTCTAATAGCAGGTTCCA
      S  I  A  *
     CTAGATATATGTAAAATCTTATCTTATCAATGTATTGCAATTTCTCTTTTTGCAAA +16
```

**Figure S1B.** The nucleotide sequence and deduced amino acid sequences of CmPDS. An asterisk (*) represents a stop codon.

```
     ATTTCCTCCTCCTCCTCTTTCTCTCTCTAGCTTTCTCTCTCTAACTTACACTCGTTA
  1  ATGGCGTCTCCTCTTCTCCTCTCTAATTCTACCTTCTCCTTCTCTCACCACCGCTCTCAC   60
      M  A  S  P  L  L  L  S  N  S  T  F  S  F  S  H  H  R  S  H
 61  AATGGCAGCAGCAGACCCATCACTTCAAACCGTAGCTTAACCCTCTCTTCTTTAAACCCA  120
      N  G  S  S  R  P  I  T  S  N  R  S  L  T  L  S  S  L  N  P
121  ATAAAAATCCCTAATTTTGATTCAAAATTGAAGAGAAACCCTAAAACCTCTTTCAGTTTG  180
      I  K  I  P  N  F  D  S  K  L  K  R  N  P  K  T  S  F  S  L
181  CTGCAATTACACCGGAGGAAGGTAATTGTAGGAGCATCGCTTGGAGAAAGGGAAACGGAA  240
      L  Q  L  H  R  R  K  V  I  V  G  A  S  L  G  E  R  E  T  E
241  GAGAAGAATTCTTCTTCGGTTGTAGCTGATGATGAGATTCAAGTTGGTGAGGATTCAGCT  300
      E  K  N  S  S  S  V  V  A  D  D  E  I  Q  V  G  E  D  S  A
301  TTCTTTGATTTGGGTGAGCAGAAGATTATTTCTTGGGTTTATTTTACGGGAATTCTGGGA  360
      F  F  D  L  G  E  Q  K  I  I  S  W  V  Y  F  T  G  I  L  G
361  GTTGTTTTGTTCGCACTCGATGTAATTTGGATTGATTCGTCGACTGGGGTTGGACTCGGG  420
      V  V  L  F  A  L  D  V  I  W  I  D  S  S  T  G  V  G  L  G
421  AAGGCGTTTGTTGATTCTGTTTCAGGGATTTCAGAAAGTCATGAGATTGTAATGCTGATC  480
      K  A  F  V  D  S  V  S  G  I  S  E  S  H  E  I  V  M  L  I
481  CTTATTTTCATTTTCGCTGCTGTCCACAGTGGCCTGGCTAGTTTCCGGGACATCGGAGAG  540
      L  I  F  I  F  A  A  V  H  S  G  L  A  S  F  R  D  I  G  E
541  AAGTTAATTGGAGAACGAGCTTACCGTGTTCTATTTGCAGGGATCTCTCTTCCTTTGGCA  600
      K  L  I  G  E  R  A  Y  R  V  L  F  A  G  I  S  L  P  L  A
601  GTTAGTACTGTTGTGTACTTCATTAACCACCGATACGATGGAATGCAATTGTGGCAGCTC  660
      V  S  T  V  V  Y  F  I  N  H  R  Y  D  G  M  Q  L  W  Q  L
661  CAGAGTGTTCCTGGGCTTCATCAACTACTGTGGATTGCGAATTTCATCTCCTTCTTTTTC  720
      Q  S  V  P  G  L  H  Q  L  L  W  I  A  N  F  I  S  F  F  F
721  TTATATCCTTCGAC  734
      L  Y  P  S
```

**Figure S1C.** The nucleotide sequence and deduced amino acid sequences of CmZ-ISO. An asterisk (*) represents a stop codon.

```
                                                   GCATGTTATTTGATC
    1 ATGGCTTCTTTTTCTTCAACTTCTTTTCTTGCTACTTCATCAGCTTCCATACTAGTAAGC
       M  A  S  F  S  S  T  S  F  L  A  T  S  S  A  S  I  L  V  S
   61 CAAAGCAGCTTCTGCAAAGAGATTAGGGGACGGGGTATTAATGGCTCTTCTTTTGTTTAT
       Q  S  S  F  C  K  E  I  R  G  R  G  I  N  G  S  S  F  V  Y
  121 CGTTGCCGTGGGCATAAGACTCCATTGAAAACTCGGAGCTTCGAAATTAAATCTTCTTCC
       R  C  R  G  H  K  T  P  L  K  T  R  S  F  E  I  K  S  S  S
  181 TTGGATACAAATGTCTCTGACATGAGGATTAACGCACCGAAAGGCTTATTTCCACCGGAA
       L  D  T  N  V  S  D  M  R  I  N  A  P  K  G  L  F  P  P  E
  241 CCTGAGTGTTATAGAGGACCAAAATTGAAAGTGGCAATCATCGGAGCCGGGCTCGCGGGC
       P  E  C  Y  R  G  P  K  L  K  V  A  I  I  G  A  G  L  A  G
  301 ATGTCAACTGCAGTGGAACTTTTGGATCAAGGCCACGAGGTTGATATATACGACTCAAGA
       M  S  T  A  V  E  L  L  D  Q  G  H  E  V  D  I  Y  D  S  R
  361 ACCTTAGTTGGCGGGAAAGTGGGTTCCTTTGTCGATAAACATAGTGGAAATCATATTGAA
       T  L  V  G  G  K  V  G  S  F  V  D  K  H  S  G  N  H  I  E
  421 ATGGGGCTACATGTTTTCTTTGGCTGCTACAATAACCTTTTCCGCTTGATGAGAAAGGTG
       M  G  L  H  V  F  F  G  C  Y  N  N  L  F  R  L  M  R  K  V
  481 GGCGCAGACGAAAATTTACTGGTGAAAGAACATACTCATACTTTTATCAATGAAGGGGGC
       G  A  D  E  N  L  L  V  K  E  H  T  H  T  F  I  N  E  G  G
  541 CGAGTCGGTGAAGTTGATTTTCGATTTCCAATTGGAGCACCATTACATGGAGTTAATGCA
       R  V  G  E  V  D  F  R  F  P  I  G  A  P  L  H  G  V  N  A
  601 TTTTTGAATACAAAACAGCTCAAGGCTTATGATAAAGCTAGGAATGCATTAGCTCTATCC
       F  L  N  T  K  Q  L  K  A  Y  D  K  A  R  N  A  L  A  L  S
  661 CGCAGCCCAATCGTTCGGGCACTTGTTGATCCAGATGGTGCAATGAGGGATATTCGAAAC
       R  S  P  I  V  R  A  L  V  D  P  D  G  A  M  R  D  I  R  N
  721 TTAGATAATATAAGCTTCTCCGATTGGATCTTGTCAAAAGGTGGCACGCGCATGTGTATC
       L  D  N  I  S  F  S  D  W  I  L  S  K  G  G  T  R  M  C  I
  781 GACCGATTGTGGGATCCTGTTTCGTACGCTCTTGGATTTATTAACAGTGACAATATGAGT
       D  R  L  W  D  P  V  S  Y  A  L  G  F  I  N  S  D  N  M  S
  841 GCTCGTTGCATGCTCACAATTCTCACTTTGATTGCCACTAAGACAGAGGCTTCTCTATTG
       A  R  C  M  L  T  I  L  T  L  I  A  T  K  T  E  A  S  L  L
  901 CACATGCTTAAAGGCTCTCCTGATGTTTACTTAACTGGTCCTATCAAAAAGTATATTACA
       H  M  L  K  G  S  P  D  V  Y  L  T  G  P  I  K  K  Y  I  T
  961 GACAAAGGTGGAAGGTTCCATCTCAGGTGGGGTTGCAGAGAAATACTTTACGATAAATCT
       D  K  G  G  R  F  H  L  R  W  G  C  R  E  I  L  Y  D  K  S
 1021 GTTAATGGAGACACGTATGTTTCAGGAATTAAAGTGTCTAAGGCGACAAATAGGAAAATA
       V  N  G  D  T  Y  V  S  G  I  K  V  S  K  A  T  N  R  K  I
 1081 GTGAAAGCTGACGTTTATGTGGCAGCATGTGATGTACCAGGAATCAAAAGACTCATTCCA
       V  K  A  D  V  Y  V  A  A  C  D  V  P  G  I  K  R  L  I  P
 1141 CCAGACTGGAGGGAATTGGAGTTTTTCGACAACATCTATAAACTAGTTGGAGTACCAATT
       P  D  W  R  E  L  E  F  F  D  N  I  Y  K  L  V  G  V  P  I
 1201 GTGACTGTACAGTTGAGATACAATGGCTGGGTTACAGAATTACAAGACCTAGAAAAGTCG
       V  T  V  Q  L  R  Y  N  G  W  V  T  E  L  Q  D  L  E  K  S
 1261 AGGCAGTTGAGTGGAGCTGCAGGTTTGGATAACCTCCTTTATAGTCCAGATGCAGATTTT
       R  Q  L  S  G  A  A  G  L  D  N  L  L  Y  S  P  D  A  D  F
 1321 TCGTGCTTCGCCGACCTTGCACTCACATCTCCAGAAGATTACTACATTGAGGGTCAAGGT
       S  C  F  A  D  L  A  L  T  S  P  E  D  Y  Y  I  E  G  Q  G
 1381 TCATTGCTCCAATGTTTGCTTACACCAGGGAGCCCTTACATGCCATTACCAAAAGATAAA
       S  L  L  Q  C  L  L  T  P  G  S  P  Y  M  P  L  P  K  D  K
 1441 ATCGTAGAAAAAGTTGCGAATCAGGTTTCAGCTTTATTCCCATCTTCTCAAGGCTTGGAA
       I  V  E  K  V  A  N  Q  V  S  A  L  F  P  S  S  Q  G  L  E
 1501 GTTATTTGGTCTAATGTTTTAAAACTTGGGCAATCGTTATATCGCGAAGGTCCTGGAAAT
       V  I  W  S  N  V  L  K  L  G  Q  S  L  Y  R  E  G  P  G  N
 1561 GATCCTTTCAGACCTGATCAAAAGACACCTGTGAAAAATTTCTTCCTTGCTGGCTCCTAC
       D  P  F  R  P  D  Q  K  T  P  V  K  N  F  F  L  A  G  S  Y
 1621 ACTAAACAGGATTACATTGATAGCATGGAAGGAGCAACTCTATCAGGAAGACAAGCATCA
       T  K  Q  D  Y  I  D  S  M  E  G  A  T  L  S  G  R  Q  A  S
 1681 GCCTATGTTTGTGAAAGCGGGGAAGAATTGGCAGCTCTTAGGAAGATGCTTTCTGCCATT
       A  Y  V  C  E  S  G  E  E  L  A  A  L  R  K  M  L  S  A  I
 1741 GAAGCCCAAGAAGTCACAAAATCTGAAAACATTATTGACGAGCTAAGTGTTGTCTGATGT
       E  A  Q  E  V  T  K  S  E  N  I  I  D  E  L  S  V  V  *
      ATAGCTATCATTTGTTGAGTTCCACTAATTCAGTCATCTCAGGCTGCCCGAAAGC +183
```

**Figure S1D.** The nucleotide sequence and deduced amino acid sequences of CmZDS. An asterisk (*) represents a stop codon.

```
-41  TCCCCAACCTCATCATCAAATCATGTACAACAACATCCACTCTCACAACCACCACCATCC
1    ATGGCCGAATTAGGGTTCCGCTCTCCATCTCTTACTTACAGTCTCCCTTCTCTATCTCAT
      M  A  E  L  G  F  R  S  P  S  L  T  Y  S  L  P  S  L  S  H
61   CCTTTCAAAACACTACCATTCAATACTCTCAATCTTTGTTGTTTTTGGAAACCCATTGAG
      P  F  K  T  L  P  F  N  T  L  N  L  C  C  F  W  K  P  I  E
121  GGTTTTGAGCTGAGGAAAAGTGGGAAATTGGGATTTCTTTTGAATAAGAGGAGAATTGGG
      G  F  E  L  R  K  S  G  K  L  G  F  L  L  N  K  R  R  I  G
181  AGTTTGACGAAATCAGTGTTGAGTGTGGAGAAAGAAGTGAGTAGAAGTGGTAGTGAAAGT
      S  L  T  K  S  V  L  S  V  E  K  E  V  S  R  S  G  S  E  S
241  GGAGAGAGAGATAATTTTGATGCGATTGTTATTGGTTCTGGAATTGGTGGATTGGTTGCT
      G  E  R  D  N  F  D  A  I  V  I  G  S  G  I  G  G  L  V  A
301  GGGACGCAGTTGGCAGTGAAAGGAGCTAGGGTTTTGGTGTTAGAGAAGTATGTGATTCCT
      G  T  Q  L  A  V  K  G  A  R  V  L  V  L  E  K  Y  V  I  P
361  GGTGGAAGTTCTGGGTTTTACCAGAAGGATGGGTATACGTTTGATGTTGGATCATCTGTG
      G  G  S  S  G  F  Y  Q  K  D  G  Y  T  F  D  V  G  S  S  V
421  ATGTTTGGGTGCGGCGATAAGGGCAATCTGAATTTGATTACTCGAGCATTAGCTGCAGTT
      M  F  G  C  G  D  K  G  N  L  N  L  I  T  R  A  L  A  A  V
481  GGGTGTAAGTTGGATATGTTACCGGACCCGTCAACTGTGCATTACCATCTACCTGACAAT
      G  C  K  L  D  M  L  P  D  P  S  T  V  H  Y  H  L  P  D  N
541  CTATCTGTTCGAGTACACAGAGAGTATAGTGACTTCGTCATGGAACTCATCAATAGATTT
      L  S  V  R  V  H  R  E  Y  S  D  F  V  M  E  L  I  N  R  F
601  CCTCATGAAAAGGAAGGAATCCATAAATTTTACGGTGAATGTTGGAAGATATTTAATGCC
      P  H  E  K  E  G  I  H  K  F  Y  G  E  C  W  K  I  F  N  A
661  TTGAACTCGCTGGAATTGAAGTCACTCGAGGAGCCACTTTACCTTTTTGGACAATTCTTT
      L  N  S  L  E  L  K  S  L  E  E  P  L  Y  L  F  G  Q  F  F
721  CGAAAACCGCTTGAATGCTTAACGCTTGCTTATAATTTGCCTCAAAATGCTGGAGCCATT
      R  K  P  L  E  C  L  T  L  A  Y  N  L  P  Q  N  A  G  A  I
781  GCTCGGAAGTTCATACGAGATCCCCAGTTGTTATCTTTTATAGATGCCGAGTGTTTCATT
      A  R  K  F  I  R  D  P  Q  L  L  S  F  I  D  A  E  C  F  I
841  GTAAGCACCACTAATGCTTTGCGGACACCGATGATCAATGCGAGCATGGTTATGTGTGAG
      V  S  T  T  N  A  L  R  T  P  M  I  N  A  S  M  V  M  C  E
901  AGGCATTTTGGTGGAATCAACTACCCCGTTGGTGGTGTTGGGAGAATTGCAAAATCCTTA
      R  H  F  G  G  I  N  Y  P  V  G  G  V  G  R  I  A  K  S  L
961  GCAAAAGGCCTGCTTGATAAGGGCAGTAACATAATTTATAAGGCAAATGTGACTAGCATT
      A  K  G  L  L  D  K  G  S  N  I  I  Y  K  A  N  V  T  S  I
1021 ATACTTAAGGATGGAAAAGCTGTAGGAGTTAAGCTTTCAGACGGAAGAGAGTTCTTTTCA
      I  L  K  D  G  K  A  V  G  V  K  L  S  D  G  R  E  F  F  S
1081 AAAACCATAATATCAAATGCAACTAGATGGGATACCTTTGGAAAGCTTTTAAAAGGAAAA
      K  T  I  I  S  N  A  T  R  W  D  T  F  G  K  L  L  K  G  K
1141 GAACTTCCGAAGGAAGAGGAAAACTTTCAGAAACTTTATGTCAAGTCCCCATCCTTTCTT
      E  L  P  K  E  E  E  N  F  Q  K  L  Y  V  K  S  P  S  F  L
1201 TCCATCCACATGGCGGTTGAAGCAAAAGTTTTGCCTCCAGACACAGATTGCCATCATTTC
      S  I  H  M  A  V  E  A  K  V  L  P  P  D  T  D  C  H  H  F
1261 ATTCTTGAGAATGACTGGACCAAATTAGAGGAGCCATATGGAAGCATCTTTTTGAGCATT
      I  L  E  N  D  W  T  K  L  E  E  P  Y  G  S  I  F  L  S  I
1321 CCAACTGTTCTTGATTCCTCCTTGGCTCCAGAAGGACGTCACATCCTTCACATATTCACG
      P  T  V  L  D  S  S  L  A  P  E  G  R  H  I  L  H  I  F  T
1381 ACATCTTCCATTGAGGATTGGAAGGGCCTCCCTCAGAAAGATTATGATGCGAAAAAGGAG
      T  S  S  I  E  D  W  K  G  L  P  Q  K  D  Y  D  A  K  K  E
1441 CACGTGGCAGATGAAATCATCAGTCGACTGGAAAAGAAACTTTTTCCAGGGCTTAAATCG
      H  V  A  D  E  I  I  S  R  L  E  K  K  L  F  P  G  L  K  S
1501 TCCATTGTTTTCAAAGAGGTAGGGACGCCCAAGACACATAGACGTTTTCTTGCTCGAGAT
      S  I  V  F  K  E  V  G  T  P  K  T  H  R  R  F  L  A  R  D
1561 AGTGGTACATATGGACCCATGCCACGTGGTACCCCAAAAGGCTTATTAGGGATGCCGTTC
      S  G  T  Y  G  P  M  P  R  G  T  P  K  G  L  L  G  M  P  F
1621 AATACAACAGCTATAGATGGGCTCTACTGTGTCGGAGATAGCTGCTTTCCAGGACAAGGT
      N  T  T  A  I  D  G  L  Y  C  V  G  D  S  C  F  P  G  Q  G
1681 GTTATAGCTGTAGCCTTTTCAGGGATAATGTGTGCTCATCGAGTGGCCGCTGATATCGGG
      V  I  A  V  A  F  S  G  I  M  C  A  H  R  V  A  A  D  I  G
1741 CTTGAGGAAAGATCCCCAATATTAGATGGTGCCTTACTTAGACTTCTTGGCTGGTTAAGG
      L  E  E  R  S  P  I  L  D  G  A  L  L  R  L  L  G  W  L  R
1801 ACAATTGCATGAAGATCATAGGCTTCATTTATTGAGTGGATGGATGAAACTTGACCTATT
      T  I  A  *
     GTATGAATGGGGGTTCTGCTATACTTCTGCTTTGGAGATCTTGAAGGAACCCATC +321
```

**Figure S1E.** The nucleotide sequence and deduced amino acid sequences of CmCrtISO. An asterisk (*) represents a stop codon.

```
-242 AGACTTTCTTGCAAAACCCACATCTTAGTTTTCTGAGATTTTTATCTGAGATTTTTTA
   1 ATGGGTACTCTACTCAGAACCCATAATAAGCTTGAGTTGCTTCACCCACTACATGGGTTT
      M  G  T  L  L  R  T  H  N  K  L  E  L  L  H  P  L  H  G  F
  61 GCAGAAAAACACAACCTTTTAACCTCTTCCAAGCTTCAAAACCATGAATATAGGTTTAGA
      A  E  K  H  N  L  L  T  S  S  K  L  Q  N  H  E  Y  R  F  R
 121 ACAGCGAAGAAAAGTCATCGAAATTGGTGTGTTAATGGTTCTGTTAAGGCCAGTAGTAGT
      T  A  K  K  S  H  R  N  W  C  V  N  G  S  V  K  A  S  S  S
 181 GCTCTTCTGGACCTTGTTCCAGAAACCAAGAAGGAGAATCTTGAATTTGAGCTTCCCATG
      A  L  L  D  L  V  P  E  T  K  K  E  N  L  E  F  E  L  P  M
 241 TATGACCCATCAAAGGGTCTTACTGTGGACCTCGCAGTTGTTGGAGGCGGTCCGGCCGGC
      Y  D  P  S  K  G  L  T  V  D  L  A  V  V  G  G  G  P  A  G
 301 CTTGCTGTGGCTCAACAGGTCTCTGAAGCTGGCCTCTCAGTTTGTTCGATAGACCCTTTT
      L  A  V  A  Q  Q  V  S  E  A  G  L  S  V  C  S  I  D  P  F
 361 CCCAAATTGATCTGGCCTAACAATTACGGTGTGTGGGTTGATGAATTTGAAGCCATGGAT
      P  K  L  I  W  P  N  N  Y  G  V  W  V  D  E  F  E  A  M  D
 421 CTTCTTGATTGCCTTGACACCACCTGGTCCGGTGCCGTTGTGTTCGTCGATGATAAATCA
      L  L  D  C  L  D  T  T  W  S  G  A  V  V  F  V  D  D  K  S
 481 AAAAAGTATCTCGACAGACCTTATGGAAGGGTTAATCGCAAACAGTTGAAATCTAAGATG
      K  K  Y  L  D  R  P  Y  G  R  V  N  R  K  Q  L  K  S  K  M
 541 ATGAAAAAATGCATAGAAAATGGTGTCAAGTTTCATCAAGCTAAGGTCATTAAGGTTATT
      M  K  K  C  I  E  N  G  V  K  F  H  Q  A  K  V  I  K  V  I
 601 CATGAGGAATCTAAGTCCCTTTTAATCTGCAATGATGGTGTAACGATTCAAGCAACTTTG
      H  E  E  S  K  S  L  L  I  C  N  D  G  V  T  I  Q  A  T  L
 661 GTTCTTGATGCCACAGGGTTTTCAAGGTGCCTGGTTCAGTATGATAAACCTTATAACCCT
      V  L  D  A  T  G  F  S  R  C  L  V  Q  Y  D  K  P  Y  N  P
 721 GGGTACCAAGTTGCTTATGGTATTTTAGCAGAAGTAGAAGAACATCCATTTGAAGTAGAT
      G  Y  Q  V  A  Y  G  I  L  A  E  V  E  E  H  P  F  E  V  D
 781 AAGATGCTTTTCATGGATTGGAGAGATTCTCATCTTAATCTGAACCATGAACAGAAAGAA
      K  M  L  F  M  D  W  R  D  S  H  L  N  L  N  H  E  Q  K  E
 841 AAGAATTCCAAAATTCCCACTTTTCTTTATGCAATGCCGTTTTCATCAAATAGAATTTTT
      K  N  S  K  I  P  T  F  L  Y  A  M  P  F  S  S  N  R  I  F
 901 CTAGAAGAAACATCTCTGGTTGCTCGACCGGGAGTTTCCATGGAAGATATTCAGGAAAGG
      L  E  E  T  S  L  V  A  R  P  G  V  S  M  E  D  I  Q  E  R
 961 ATGGCTCTTAGATTGAAGCACTTGGGTATAAAGGTTAAAAGCATTGAAGAGGATGAACGG
      M  A  L  R  L  K  H  L  G  I  K  V  K  S  I  E  E  D  E  R
1021 TGTGTTATTCCAATGGGTGGGCCACTTCCTGTAATCCCTCAAAGGGTTGTTGGAATAGGT
      C  V  I  P  M  G  G  P  L  P  V  I  P  Q  R  V  V  G  I  G
1081 GGCACTGCCGGCATGGTTCATCCTTCGACAGGATACATGGTAGCGAGAACTCTTGCGGCA
      G  T  A  G  M  V  H  P  S  T  G  Y  M  V  A  R  T  L  A  A
1141 GCCCCAATAGTTGCAAAGTCGATAGTTCAGTATCTTGGATCTGAACAAGGATTTTCTGGT
      A  P  I  V  A  K  S  I  V  Q  Y  L  G  S  E  Q  G  F  S  G
1201 AATGAATTATCTGCAGGAATTTGGAAAAATTTGTGGCCTATAGAGAGAAGGAGGCAACGG
      N  E  L  S  A  G  I  W  K  N  L  W  P  I  E  R  R  R  Q  R
1261 GAGTTCTTCTGTTTTGGTATGGATATTCTGCTTAAACTTGATTTACAAGGAACAAGGAGC
      E  F  F  C  F  G  M  D  I  L  L  K  L  D  L  Q  G  T  R  S
1321 TTCTTTGATGCATTTTTTGACCTTGAACCTCATTATTGGCATGGATTCTTGTCCTCTCGA
      F  F  D  A  F  F  D  L  E  P  H  Y  W  H  G  F  L  S  S  R
1381 TTGTTTTTTCCGGAACTTATATATTTCGGGCTTTCTCTGTTCTCACATGCCTCTAATGCT
      L  F  F  P  E  L  I  Y  F  G  L  S  L  F  S  H  A  S  N  A
1441 TCAAGGATTGAGATTATGGCCAAGGGCACTGTTCCTTTAGGTAAGATGATCAACAACTTA
      S  R  I  E  I  M  A  K  G  T  V  P  L  G  K  M  I  N  N  L
1501 ATACGGGATGGAGAATAGGATTGAAGGAATTTAAGTCTGTTGAATGTAAGATGAAATGAA
      I  R  D  G  E  *
     GCATCTACATCTAAATCTTTACTTTTTGTTTGCTGTCAAATGTCAATGTAACCACT +41
```

**Figure S1F.** The nucleotide sequence and deduced amino acid sequences of CmLCYB. An asterisk (*) represents a stop codon.

```
 -75 CGGTCTGACACTAACACTTTCTCTGCCTAGCTTTTTAAAGCAGAGAGTAATGTGTCGGTC
     TCTTGCTTCTTAAAAGCAGAGTAATGTAACAGAGGAGCTTCATTAACATTACTAGTAACA
   1 ATGGAGTACTGTATTGGAGCTCGTAGTAACATTACAGCAATGGCGATTTCTCCTTCTTGC
      M  E  Y  C  I  G  A  R  S  N  I  T  A  M  A  I  S  P  S  C
  61 CCTGTTTTGAGGTCAAGAACAAGGAGACTTCTCAGTACAACAAGTATCTCTTCTTCTTCT
      P  V  L  R  S  R  T  R  R  L  L  S  T  T  S  I  S  S  S  S
 121 TCTTCTCACCTAATTCATGGATATTCCTTTTCTTCTCTCAATGTGAAGGCTTGTCATGCC
      S  S  H  L  I  H  G  Y  S  F  S  S  L  N  V  K  A  C  H  A
 181 GATTCTACAACCCAAAGTTGTAGTGTTTCTGTTAAAGAAGGTGGGTTTGTTGATGAAGAA
      D  S  T  T  Q  S  C  S  V  S  V  K  E  G  G  F  V  D  E  E
 241 GATTATATCAAAGCTGGAGGTTCTGAGATTCTGTTTGTTCAAATGCAACAGACAAAGCCA
      D  Y  I  K  A  G  G  S  E  I  L  F  V  Q  M  Q  Q  T  K  P
 301 ATGGAAGAGCTAACCAAGATTTCCGACAAGTTGCCACCGATATCCATTGGAGACTCTATA
      M  E  E  L  T  K  I  S  D  K  L  P  P  I  S  I  G  D  S  I
 361 CTAGATCTGGTAATAATTGGTTGTGGCCCGGCCGGTCTTTCCCTGGCTGCAGAAGCAGCT
      L  D  L  V  I  I  G  C  G  P  A  G  L  S  L  A  A  E  A  A
 421 AAATTAGGCTTGCAAGTTGGGCTAATTGGACCTGATCTACCTTTTACAAATAATTATGGC
      K  L  G  L  Q  V  G  L  I  G  P  D  L  P  F  T  N  N  Y  G
 481 GTGTGGGCGGATGAATTTAAAGATCTTGGGCTAGAAAGGTGTATTGAGCATGTTTGGAAA
      V  W  A  D  E  F  K  D  L  G  L  E  R  C  I  E  H  V  W  K
 541 GATACAATTGTATATCTTGATAGTGATGAACCGATTATGATTGGCCGTGCTTATGGAAGA
      D  T  I  V  Y  L  D  S  D  E  P  I  M  I  G  R  A  Y  G  R
 601 GTTAGTCGACAGTTGCTATATGAAGAGCTGTTGAAAAGGTGTGTAGAGTCGGGTGTTTCG
      V  S  R  Q  L  L  Y  E  E  L  L  K  R  C  V  E  S  G  V  S
 661 TACCTTAACTCTAAAGTGGAAAGGATAATTGAAGCTACAGAGGGACATAGTCTTGTAGCG
      Y  L  N  S  K  V  E  R  I  I  E  A  T  E  G  H  S  L  V  A
 721 TGTGAAAGCGGGATTGTTATCCCTTGCAGGCTTGCTACTGTGGCATCTGGAGCAGCTTCA
      C  E  S  G  I  V  I  P  C  R  L  A  T  V  A  S  G  A  A  S
 781 GGGAAACTATTAAAGTATGAAGTAGGAGGTCCTCGAGTTTCTGTACAGACAGCTTATGGT
      G  K  L  L  K  Y  E  V  G  G  P  R  V  S  V  Q  T  A  Y  G
 841 TTGGAGGTTGAGGTGGAAAACTGTCCATATGATCCCAGCCTAATGGTTTTCATGGATTAT
      L  E  V  E  V  E  N  C  P  Y  D  P  S  L  M  V  F  M  D  Y
 901 AGAGACTACATTAAACCAAAAGTCCAATGCTTGGAAGAAGAATACCCTTCATTTCTGTAT
      R  D  Y  I  K  P  K  V  Q  C  L  E  E  E  Y  P  S  F  L  Y
 961 GCCATGCCCATGTCACCAACAAGAGTTTTCTTTGAGGAAACTTGTCTGGCTTCCAGAAAT
      A  M  P  M  S  P  T  R  V  F  F  E  E  T  C  L  A  S  R  N
1021 GCCATTCCTTTTGATCTATTGAAGAAGAAACTGATGTCAAGATTGAAGACGATGGGAGTT
      A  I  P  F  D  L  L  K  K  K  L  M  S  R  L  K  T  M  G  V
1081 CGAATTACAAAAGTTTATGAAGAGGAGTGGTCTTATATTCCTGTTGGTGGTTCCTTACCA
      R  I  T  K  V  Y  E  E  E  W  S  Y  I  P  V  G  G  S  L  P
1141 AACACCGAGCAAAAGAACCTTGCATTTGGTGCTGCAGCCAGCATGGTGCATCCAGCCACC
      N  T  E  Q  K  N  L  A  F  G  A  A  A  S  M  V  H  P  A  T
1201 GGTTATTCAGTTGTTAGATCTTTGTCTGAAGCTCCAAGATATGCTTCTGTAATTGCAAAG
      G  Y  S  V  V  R  S  L  S  E  A  P  R  Y  A  S  V  I  A  K
1261 ATTTTGAAGCAATCTCATGAAAGGAGCACCAAGAATATCTCAGTACAAGCATGGAACACT
      I  L  K  Q  S  H  E  R  S  T  K  N  I  S  V  Q  A  W  N  T
1321 CTCTGGCCACAAGAGAGAAAACGACAAAGAGCATTCTTTCTCTTTGGATTAGAGCTGATT
      L  W  P  Q  E  R  K  R  Q  R  A  F  F  L  F  G  L  E  L  I
1381 TTGCAACTCGATATTGAAGGCATAAGAACATTCTTCCAAACTTTTTTCAGCTTGCCAAAT
      L  Q  L  D  I  E  G  I  R  T  F  F  Q  T  F  F  S  L  P  N
1441 TGGATGTGGAAGGGCTTCCTCGGATCTTCACTGTCATCCACTGATCTTCTACTATTTGCC
      W  M  W  K  G  F  L  G  S  S  L  S  S  T  D  L  L  L  F  A
1501 CTCTATATGTTTATATTAGCACCAAACAGTTTGCGAATGGGCCTTGTCAGACATCTACTA
      L  Y  M  F  I  L  A  P  N  S  L  R  M  G  L  V  R  H  L  L
1561 TCTGATCCTACTGGAGCAACTATGGTAAAAGCATATCTCACTTAGTAGCTCAAGCCCTTA
      S  D  P  T  G  A  T  M  V  K  A  Y  L  T  *
     ATATATTTCATAAGCCTGTTTCTTTATTTAGACTATAATCTCACACACCCACTGTACTGA
     ACGTCTCCAAAGTCCGTCTGTCTGTGTATATAAAACTTCTTTATGGGGGGGGATC +298
```

**Figure S1G.** The nucleotide sequence and deduced amino acid sequences of CmLCYE. An asterisk (*) represents a stop codon.

```
-72 ATTCCTTTTATACTCCTCCAGATTTCTATCTCCCTCTCTAAGACTCTAACCACTCTCTCTCTCTCTC
    TCTGTGAGTACTGACCTTTCAAAGTTCAACAAACTGAATAAGCTCCAATCTCAAACGTCA
  1 ATGGCGGCCGGATTCTCTGTCGCCATAAGCGCCAGAACGTTCCGGTTTGGTCAGAATCCT
     M  A  A  G  F  S  V  A  I  S  A  R  T  F  R  F  G  Q  N  P
 61 TTCATAGGTTCAAAACCCAATTCTCTCATAAACCCATCTTCATTTCTCTCTCCATCAATT
     F  I  G  S  K  P  N  S  L  I  N  P  S  S  F  L  S  P  S  I
121 CGCCGACCTAATAAATTCTTCACGGCGAAGAAGAAGAGCACCTTTACTGTCTATTTTGTA
     R  R  P  N  K  F  F  T  A  K  K  K  S  T  F  T  V  Y  F  V
181 TTGGAAGAACAAAAACAGGGGATTAGTAGATTTATTGAAGAAGAAGAAGATAAAGCAGAA
     L  E  E  Q  K  Q  G  I  S  R  F  I  E  E  E  E  D  K  A  E
241 GCTGATGTTAGTTCATCATCAGAGGAATTGAATAGGGTCTCAGTACGAGTTGCAGAGAAA
     A  D  V  S  S  S  S  E  E  L  N  R  V  S  V  R  V  A  E  K
301 TTAGCCAGAAAGAAATCAGAACGACACACTTATCTTGTCGCAGCAATTATGTCTACTCTT
     L  A  R  K  K  S  E  R  H  T  Y  L  V  A  A  I  M  S  T  L
361 GGAATAACTTCTATGGCTGTTGCTGCTGTTTATTACAGATTTTCATGGCAAATGGAGGGT
     G  I  T  S  M  A  V  A  A  V  Y  Y  R  F  S  W  Q  M  E  G
421 GGAGAGGTTCCTGTTTCTGAGATGTTTGGTACATTTGCTCTAGCTGTTGGTGCTGCTGTT
     G  E  V  P  V  S  E  M  F  G  T  F  A  L  A  V  G  A  A  V
481 GGGATGGAATTTTGGGCAAGATGGGCTCACAAAGCACTCTGGCACGCTTCTTTATGGCAC
     G  M  E  F  W  A  R  W  A  H  K  A  L  W  H  A  S  L  W  H
541 ATGCACGAGTCTCATCATCAACCAAGAGAAGGTCCTTTTGAACTAAATGATGTTTTCGCC
     M  H  E  S  H  H  Q  P  R  E  G  P  F  E  L  N  D  V  F  A
601 ATTATCAACGCTGTTCCAGCTATAGCTCTTCTCAATTATGGTTTCTTTCACAAAGGCCTC
     I  I  N  A  V  P  A  I  A  L  L  N  Y  G  F  F  H  K  G  L
661 TTTCCTGGTCTCTGTTTTGGTGCTGGACTTGGAATTACTGTATTTGGTATGGCCTACATG
     F  P  G  L  C  F  G  A  G  L  G  I  T  V  F  G  M  A  Y  M
721 TTTGTTCATGATGGTCTTGTTCACCGTAGATTTCCAGTGGGACCCATTGCTAATGTTCCT
     F  V  H  D  G  L  V  H  R  R  F  P  V  G  P  I  A  N  V  P
781 TATTTTCGGAAAGTTGCTGCTGCACACAAAATTCATCACATGGACAAATTTGAGGGAGTT
     Y  F  R  K  V  A  A  A  H  K  I  H  H  M  D  K  F  E  G  V
841 CCATATGGGTTGTTTTTAGGACCTATGGAATTAGAAGAGGTAGGAGGTAATGAAGAATTA
     P  Y  G  L  F  L  G  P  M  E  L  E  E  V  G  G  N  E  E  L
901 GAGAAGGAAATTAGCCGTAGGATCAAGCAATCCAACGATTCGAGATGAAGTTCGCGGTGG
     E  K  E  I  S  R  R  I  K  Q  S  N  D  S  R  *
    GGTCCATTTTAATGTTTATGGTGTGTGATTAACGGTTTTGGTTACAATGTAAAAA +6
```

**Figure S1H.** The nucleotide sequence and deduced amino acid sequences of CmCHXB. An asterisk (*) represents a stop codon.

```
-31  AAATGAGAAGCAATGTGAACAGAATTGTTTAATCTTTTCATTTCTGTCTCACAATCCGCC
  1  ATGACATCTTCTTCATCTTGTACTTCGCTAACCCTCTCTTCTCTCTCTTCTCTTTCTAAC
      M  T  S  S  S  S  C  T  S  L  T  L  S  S  L  S  S  L  S  N
 61  CCTAACCACCAAATCCTCCAAAACAAACCCATCTTCTTCTTCCCAAGAAACCCATCTCAA
      P  N  H  Q  I  L  Q  N  K  P  I  F  F  F  P  R  N  P  S  Q
121  ATTCTCTCCATTAAATCCTCCATTAAAAAAGAGTCTTCTTATTCTAGCAGTAATGGGTCA
      I  L  S  I  K  S  S  I  K  K  E  S  S  Y  S  S  S  N  G  S
181  TGGGTTAGTCCAGATTGGCTCACTTCTTTAACCAAATCACTCACCATCAGTACCAGTCAA
      W  V  S  P  D  W  L  T  S  L  T  K  S  L  T  I  S  T  S  Q
241  GATGATTCAGGGATACCCATTGCCAGTGCTAAGCTTGATGATGTGTCTGATCTCTTGGGT
      D  D  S  G  I  P  I  A  S  A  K  L  D  D  V  S  D  L  L  G
301  GGTGCTCTTTTCCTTCCATTGTTCAAATGGATGAATGAATATGGACCCATTTATCGTCTT
      G  A  L  F  L  P  L  F  K  W  M  N  E  Y  G  P  I  Y  R  L
361  GCAGCTGGACCCCGTAATTTTGTGGTTGTTAGTGACCCTGCCATTGCCAAGCATGTTCTG
      A  A  G  P  R  N  F  V  V  V  S  D  P  A  I  A  K  H  V  L
421  AGGAACTATGGGAAGTATGGTAAGGGACTTGTAGCTGAAGTCTCTGAGTTCTTATTCGGC
      R  N  Y  G  K  Y  G  K  G  L  V  A  E  V  S  E  F  L  F  G
481  TCTGGGTTCGCCATTGCTGAAGGCCCTCTTTGGACGGCGAGGCGGAGAGCTGTGGTTCCA
      S  G  F  A  I  A  E  G  P  L  W  T  A  R  R  R  A  V  V  P
541  TCTCTTCACAAGAAGTACTTGTCCATAATGGTTGATAGGGTATTTTCCAAGTGTGCCCAG
      S  L  H  K  K  Y  L  S  I  M  V  D  R  V  F  S  K  C  A  Q
601  AGGTTAGTGGAGCAGCTTAAAACAGATGCAGACAATGGTTCTCCCGTGAATATGGAGGCG
      R  L  V  E  Q  L  K  T  D  A  D  N  G  S  P  V  N  M  E  A
661  AAGTTTTCTCAATTAACTTTAGATGTTATAGGTTTGTCGGTGTTTAACTACAATTTTGAT
      K  F  S  Q  L  T  L  D  V  I  G  L  S  V  F  N  Y  N  F  D
721  GCACTTACTGCGGATAGTCCTGTTATCGATGCTGTCTACACTGCATTGAAAGAGGCAGAG
      A  L  T  A  D  S  P  V  I  D  A  V  Y  T  A  L  K  E  A  E
781  GCTCGTTCGACGGATCTTTTACCCTATTGGAAGATTGACGCATTATGCAAAATAATTCCA
      A  R  S  T  D  L  L  P  Y  W  K  I  D  A  L  C  K  I  I  P
841  AGACAAATAAAAGCTCAAAATGCAGTTAATGTTATTAGAAC
      R  Q  I  K  A  Q  N  A  V  N  V  I  R
```

**Figure S1I.** The nucleotide sequence and deduced amino acid sequences of CmCHXE. An asterisk (*) represents a stop codon.

```
-189  CTCTTCTTCTTCATTCATCAGCTCATCACAACAAGAAGAACTGAAGAACTTGAACTATTA
   1  ATGGCTTTAACAGCTTTATACAGTTCACTCAACCCCTCATCAACAATATTCTCAAAAACC
       M  A  L  T  A  L  Y  S  S  L  N  P  S  S  T  I  F  S  K  T
  61  CACCTTCCAATACCAACAGCCAAAGACTTCTCTGAAGAAATTTTGCCTTCTCTTCATTAC
       H  L  P  I  P  T  A  K  D  F  S  E  E  I  L  P  S  L  H  Y
 121  AACTATTTATTTAGAAGTAAACCCTTTGATCAGAAAAAGAGAAAACCAGTGAAAATTTCA
       N  Y  L  F  R  S  K  P  F  D  Q  K  K  R  K  P  V  K  I  S
 181  GCAGTAGTAGCTCCAGAAGCTGTTACTTCCACTCAAAATGGAATTAATGGTGGTGGGTTG
       A  V  V  A  P  E  A  V  T  S  T  Q  N  G  I  N  G  G  G  L
 241  TCGAAGAAACTGCGGATCCTAATTGCTGGTGGTGGGATTGGTGGGTTGGTTTTTGCTTTG
       S  K  K  L  R  I  L  I  A  G  G  G  I  G  G  L  V  F  A  L
 301  GCTGCTAAGAGAAAAGGGTTTGATGTGTTGGTATTTGAGAGGGATGTTAGTGCTATAAGA
       A  A  K  R  K  G  F  D  V  L  V  F  E  R  D  V  S  A  I  R
 361  GGAGAGGGCCAATATAGGGGCCCAATTCAGATACAAAGTAATGCCTTGGCTGCATTAGAA
       G  E  G  Q  Y  R  G  P  I  Q  I  Q  S  N  A  L  A  A  L  E
 421  GCTATTGATATGGAGGTGGCTGAGGAAGTTTTGAAAGCTGGCTGTATTACTGGTGATAGG
       A  I  D  M  E  V  A  E  E  V  L  K  A  G  C  I  T  G  D  R
 481  ATTAATGGATTGGTTGATGGGATCTCAGGTGCTTGGTATATCAAGTTTGATACATTCACA
       I  N  G  L  V  D  G  I  S  G  A  W  Y  I  K  F  D  T  F  T
 541  CCTGCGGTGGAACGGGGGCTCCCTGTCACAAGGGTTATTAGCCGAATGACTTTGCAAGAA
       P  A  V  E  R  G  L  P  V  T  R  V  I  S  R  M  T  L  Q  E
 601  ATTTTTGCCAAGGCAGTTGGGCAGGATTCCATTATTAATGACAGTAATGTTGTTGATTTT
       I  F  A  K  A  V  G  Q  D  S  I  I  N  D  S  N  V  V  D  F
 661  GAAGATGATGGAAACAAGGTTACTGTAGTACTTGAGAACGGACAGCGATATGAAGGTGAT
       E  D  D  G  N  K  V  T  V  V  L  E  N  G  Q  R  Y  E  G  D
 721  CTTTTAGTTGGAGCTGATGGTATCTGGTCAAAGGTACGGAAGAATTTATTTGGGCCCAAG
       L  L  V  G  A  D  G  I  W  S  K  V  R  K  N  L  F  G  P  K
 781  GATGCGCAGTACTCTGGTTACACTTGTTATACTGGAATCTGCGATTTTGTTCCTCCAGAT
       D  A  Q  Y  S  G  Y  T  C  Y  T  G  I  C  D  F  V  P  P  D
 841  ATTGAAAGTGTTGGGTACCGAGTATTTTTGGGTCACAAGCAATACTTTGTGTCTTCAGAT
       I  E  S  V  G  Y  R  V  F  L  G  H  K  Q  Y  F  V  S  S  D
 901  GTGGGTGCTGGAAAGATGCAGTGGTATGCATTCCATAACGAACCAGCTGGTGGCACTGAT
       V  G  A  G  K  M  Q  W  Y  A  F  H  N  E  P  A  G  G  T  D
 961  GCTCCAAATGGTAAAAAGGAACGATTACTTCAGATATTTGGTTCCTGGTGTGATAATGTG
       A  P  N  G  K  K  E  R  L  L  Q  I  F  G  S  W  C  D  N  V
1021  GTTGACTTGTTAAATGCCACGGATGAGGATGCAATTCTTCGACGTGACATATATGATCGA
       V  D  L  L  N  A  T  D  E  D  A  I  L  R  R  D  I  Y  D  R
1081  GTGCCTATTTTTACCTGGGGAAAGGGTCGTGTAACCTTGCTGGGTGATTCCGTTCATGCT
       V  P  I  F  T  W  G  K  G  R  V  T  L  L  G  D  S  V  H  A
1141  ATGCAGCCAAATATGGGTCAAGGTGGATGCATGGCTATTGAGGATAGCTATCAACTTGCT
       M  Q  P  N  M  G  Q  G  G  C  M  A  I  E  D  S  Y  Q  L  A
1201  GTAGAGCTCGAGAAATCTTGGAACGAAAGTGTAGAATCTGGAATCTCTATTGATGTCACA
       V  E  L  E  K  S  W  N  E  S  V  E  S  G  I  S  I  D  V  T
1261  TCTGCTCTAAAGCGTTATGAGAAAGAAAGACGATTGCGTGTTGCAGTCATCTATGGAATG
       S  A  L  K  R  Y  E  K  E  R  R  L  R  V  A  V  I  Y  G  M
1321  GCAAGGATGGCCGCAATAATGGCGTCCACTTATAAACCATATTTGGGTGTAGGGCTTGGT
       A  R  M  A  A  I  M  A  S  T  Y  K  P  Y  L  G  V  G  L  G
1381  CCACTGTCGTTTATTACAAAATTTCGGATACCACATCCAGGAAGAGTTGGCGGGAGATTT
       P  L  S  F  I  T  K  F  R  I  P  H  P  G  R  V  G  G  R  F
1441  TTCATTGACATAGGCATGCCCTTAATGCTAAGTTGGGTCTTAGGTGGTAACAGTTCAAAG
       F  I  D  I  G  M  P  L  M  L  S  W  V  L  G  G  N  S  S  K
1501  CTTGAAGGGAGGTCAGTATGCTGTCGACTCTCGGACAAGGCAAACGATCAATTAAGAAGA
       L  E  G  R  S  V  C  C  R  L  S  D  K  A  N  D  Q  L  R  R
1561  TGGTTCGAAGATGATGATGCACTGGAGCGTGCCCTTAATGGAGAGTGGTTTCTTTTTCCC
       W  F  E  D  D  D  A  L  E  R  A  L  N  G  E  W  F  L  F  P
1621  TCTGAAAATAATAGTGGTTCCCAAGAACCTATTCATTTGAGTAGAGATGAAAACAAGCCG
       S  E  N  N  S  G  S  Q  E  P  I  H  L  S  R  D  E  N  K  P
1681  TGCCTTATTGGGAATGTTTCACACGCTGACGTTGCAGGAACGTCGATAGCCATACCTTCA
       C  L  I  G  N  V  S  H  A  D  V  A  G  T  S  I  A  I  P  S
1741  CCTCAGATTTCCAAAATGCACGCTCGTATAAGCTATAAAGATGGCGCCTTCTATCTCACC
       P  Q  I  S  K  M  H  A  R  I  S  Y  K  D  G  A  F  Y  L  T
1801  GATTTGCAGAGTGAACACGGCACTTGGATCACCGATATCGAGGGAAGGCGTTACCGTGTG
       D  L  Q  S  E  H  G  T  W  I  T  D  I  E  G  R  R  Y  R  V
1861  CCTCCCAATCTCCCCTCTAGATTCCGTCCATCTGACGTAATTGAGTTTGGTTCTGAGGCC
       P  P  N  L  P  S  R  F  R  P  S  D  V  I  E  F  G  S  E  A
1921  ACCTTTAAAGTGAAGGTGCTGGCGGTTCCTTCAAATATTCCAGACAAGAAACAAACTCCA
       T  F  K  V  K  V  L  A  V  P  S  N  I  P  D  K  K  Q  T  P
1981  GTCCTCAGTGCCACATGACGATTGGAAGTTTCTGCTTTTGTATCACCCTTATACTGTACA
       V  L  S  A  T  *
      GCAAGCATTCACACTGCCAGAGCAAAATAGCGTGCCACTGTTTTCGTACGGATA +135
```

**Figure S1J.** The nucleotide sequence and deduced amino acid sequences of CmZEP. An asterisk (*) represents a stop codon.

```
 -63  TCGTCTCCTCTGCAAGTACTTTTGCTGCTGTCATGCAAAATATTGTCTACTTCATTTCTT
      CAATATCTTCTCTCTTCAATTGTCCTCACCAGGTAAATTTGGCCTTCAAACCATTGTAAT
   1  ATGGCATCTGCCGCATATTCAACCTGCTTATCCTATGACAAGAATGTTGTTACATGTATT
       M  A  S  A  A  Y  S  T  C  L  S  Y  D  K  N  V  V  T  C  I
  61  GAGTTGCTAAAGCAAAGCAATGAAAGGTTTCTTCGAAGGTCAAACTGGAAGCATCAAGGT
       E  L  L  K  Q  S  N  E  R  F  L  R  R  S  N  W  K  H  Q  G
 121  TTAATTAAGATGAAATTGTGGTCCAATACCATAGATTCAAAGATAACCCAGTGGACTAGA
       L  I  K  M  K  L  W  S  N  T  I  D  S  K  I  T  Q  W  T  R
 181  TCTTACAAAAATTATTTCCAGTTGAAGTCTAGAGGTTCTCATGTCATATTAGGTGGGAAA
       S  Y  K  N  Y  F  Q  L  K  S  R  G  S  H  V  I  L  G  G  K
 241  GACCATAGTGCTTCAGCACGTAGTGGCAATACGGAAGGCCGCTGTCCTGAGGTAGTGGAA
       D  H  S  A  S  A  R  S  G  N  T  E  G  R  C  P  E  V  V  E
 301  GCTGATTTTCCCGATCCGATGCTGTTGGGTGTTATGCAGCAGTGGAGGCAAATGCAGGTC
       A  D  F  P  D  P  M  L  L  G  V  M  Q  Q  W  R  Q  M  Q  V
 361  ATGGCAGTAGCTGGTGTACTTACATGCATATTCTTGGTTGTGCCATCTGCTGATGCTGTA
       M  A  V  A  G  V  L  T  C  I  F  L  V  V  P  S  A  D  A  V
 421  GATGCACTGAAGACATGTGCTTGTTTATTGAAAGAGTGCAGGGTGGAATTGGCCAAGTGT
       D  A  L  K  T  C  A  C  L  L  K  E  C  R  V  E  L  A  K  C
 481  ATCGCAAACCCATCTTGTGCAGCCAATGTTGCATGTTTACAGACCTGCAACAACCGGCCC
       I  A  N  P  S  C  A  A  N  V  A  C  L  Q  T  C  N  N  R  P
 541  GATGAGACCGAATGCCAGATTAAATGTGGTGATCTGTTTGAGAATAAAGTCGTAGATGAG
       D  E  T  E  C  Q  I  K  C  G  D  L  F  E  N  K  V  V  D  E
 601  TTCAATGAATGTGCTGTTTCACGCAAGAAATGCGTTCCACAAAAGTCAGATGTTGGTGAA
       F  N  E  C  A  V  S  R  K  K  C  V  P  Q  K  S  D  V  G  E
 661  TTTCCAATACCAGATCCTGCGGCACTCGTCAAAAATTTCAATGTTGGGGATTTTAGTGGA
       F  P  I  P  D  P  A  A  L  V  K  N  F  N  V  G  D  F  S  G
 721  AAGTGGTATATTTCTAGTGGCTTAAATCCCACTTTCGATGCTTTTGATTGCCAATTGCAC
       K  W  Y  I  S  S  G  L  N  P  T  F  D  A  F  D  C  Q  L  H
 781  GAGTTCCATATGGAGGGCAACAAACTCGTAGGCAATTTATCATGGCGTATACGGACTCCT
       E  F  H  M  E  G  N  K  L  V  G  N  L  S  W  R  I  R  T  P
 841  GATAGTGGTTTTTTCACTCGAACAGCTACGCAGAAGTTTGTGCAGGATCCATTGCAGCCT
       D  S  G  F  F  T  R  T  A  T  Q  K  F  V  Q  D  P  L  Q  P
 901  GGAATTCTTTACAATCATGACAACGAGTTCCTTCACTACCAAGATGATTGGTACGTCTTG
       G  I  L  Y  N  H  D  N  E  F  L  H  Y  Q  D  D  W  Y  V  L
 961  TCATCCAAGATAGAAAACAATCCGGATGATTACGTGTTTGTATACTATCGTGGTAAGAAT
       S  S  K  I  E  N  N  P  D  D  Y  V  F  V  Y  Y  R  G  K  N
1021  GATGCATGGGATGGATATGGCGGTGCTTTTGTGTACACGAGAAGCGCAGTTTTACCTGAT
       D  A  W  D  G  Y  G  G  A  F  V  Y  T  R  S  A  V  L  P  D
1081  AGCATAGTACCCGAACTAGAAAGAGCAGCAAAAAGTGTAGGACGGGATTTCAACACGTTC
       S  I  V  P  E  L  E  R  A  A  K  S  V  G  R  D  F  N  T  F
1141  ATCAGAACAGATAATACTTGTGGGCCTGAACCTCCTTTAGTTGAAAGACTTGAGAAGACC
       I  R  T  D  N  T  C  G  P  E  P  P  L  V  E  R  L  E  K  T
1201  GTGGAAGAAGGAGAGAGGACGATCATACGAGAGGTAGAGGAAATAGAAGGAGAGATAGAA
       V  E  E  G  E  R  T  I  I  R  E  V  E  E  I  E  G  E  I  E
1261  AGTGAGGTTGAGAAAGTGGAGAAGACTGAAATGAATCTGCTCCAAAGGTTGTTTGATGGA
       S  E  V  E  K  V  E  K  T  E  M  N  L  L  Q  R  L  F  D  G
1321  TTTAAGGAGCTCCAGCAAGATGAAGAAAACCTTCTAAAAGGTCTTAGCCAGGAAGAGATG
       F  K  E  L  Q  Q  D  E  E  N  L  L  K  G  L  S  Q  E  E  M
1381  GAGATTTTAAATGATCTTAAGATGGAAGCAAAAGAGGTAGAAAAACTCTTTGGGCAGGCA
       E  I  L  N  D  L  K  M  E  A  K  E  V  E  K  L  F  G  Q  A
1441  ATACCACTGAGGAAGCTAAGGTAGAATGCAGTTGGATGAATTTTAAGCCTTGTTACAAGT
       I  P  L  R  K  L  R  *
      ATCTTATAAGCAACATGCTTAATGAGTTCCACTCTAAATGAATTTGAGTGCTTAGAAACC
      CCAAAGTGTTATTGCAAAGTTACATTGCTTTTAGGAAAGAAGTGGGGCTCTGGAA +136
```

**Figure S1K.** The nucleotide sequence and deduced amino acid sequences of CmVDE. An asterisk (*) represents a stop codon.

```
-44 1   GATTCTTTTTTGTTCATACCCCTGAAAATATTTCTTAGTTCTCATTTGAACATCAATAGA
          D  S  F  L  F  I  P  L  K  I  F  L  S  S  H  L  N  I  N  R
    61  ACTTGGCTAGTTTTCTCAAAATTTCACAGCTTCTCCCATCTCAGGTTTCTCATGTCGACT
          T  W  L  V  F  S  K  F  H  S  F  S  H  L  R  F  L  M  S  T
   121  CTTCTCAGGTCTCCTATGCTTTCCACTCTTACAGTCCCAACCCTCAAAACTGCTCCATCA
          L  L  R  S  P  M  L  S  T  L  T  V  P  T  L  K  T  A  P  S
   181  TTCTTCCCAATCACTTCCACCAATTCAAGAAAACTAAAGCAACCCAGAATTCAAATCAGT
          F  F  P  I  T  S  T  N  S  R  K  L  K  Q  P  R  I  Q  I  S
   241  TCTAAATTTAACAGCTTCCTAGATCTGAAGCCTGAATCAAAACCCGAGTTTTTAACCTTT
          S  K  F  N  S  F  L  D  L  K  P  E  S  K  P  E  F  L  T  F
   301  GATTTACCGTTCCACGACAAGTCTGATCGTGTTAAGTATGATGTGATCATCATTGGTGCA
          D  L  P  F  H  D  K  S  D  R  V  K  Y  D  V  I  I  I  G  A
   361  GGACCAGCTGGTCTTCGTTTAGCTGAAAG
          G  P  A  G  L  R  L  A  E
```

**Figure S1L.** The nucleotide sequence and deduced amino acid sequences of CmCCS. An asterisk (*) represents a stop codon.

```
-1 1  ATGGATTCCCTCTCTTCTTCTTTCTTCACAAAATTTCATCCACATAAACTCATTCTCTCT
       M  D  S  L  S  S  S  F  F  T  K  F  H  P  H  K  L  I  L  S
  61  CCTCCTTCTCCCACTAAAAATCCTCTCTTCTACATATCTGCTGTAAATGTAGAAGATAGA
       P  P  S  P  T  K  N  P  L  F  Y  I  S  A  V  N  V  E  D  R
 121  CTGACCATCAATGATCCAAAACCAACACCTTCATCTCCATTATCTCAAAACCCTACTAAC
       L  T  I  N  D  P  K  P  T  P  S  S  P  L  S  Q  N  P  T  N
 181  CGAACACCAATTACATCATCATCAGTAGCAACAAAAGCTAAAAACGTTGGAAAAGATCCA
       R  T  P  I  T  S  S  S  V  A  T  K  A  K  N  V  G  K  D  P
 241  AAGAAATCAACAATAACTAGAAGAAGATCAATAACAACAACAGATCTTCCATCAGTTCCA
       K  K  S  T  I  T  R  R  R  S  I  T  T  T  D  L  P  S  V  P
 301  ACTAGCATTTTCAACGCATTTGATAGCATCATAAATAACTTCATTGACCCGGCAATCCGT
       T  S  I  F  N  A  F  D  S  I  I  N  N  F  I  D  P  A  I  R
 361  CCTTCTGTAGACCCAAAACACGTACTTGCTAAAAATTTCGCACCCGTTGATGAACTACCT
       P  S  V  D  P  K  H  V  L  A  K  N  F  A  P  V  D  E  L  P
 421  CCAACAAAGTGCACTGTAATTCAAGGTGCCATTCCACCCTCCATTAATGGTGCTTACATC
       P  T  K  C  T  V  I  Q  G  A  I  P  P  S  I  N  G  A  Y  I
 481  CGCAACGGGCCAAACCCACAACATCTTCCACATGGACCTTACCATTTATTCGATGGAGAC
       R  N  G  P  N  P  Q  H  L  P  H  G  P  Y  H  L  F  D  G  D
 541  GGCATGCTTCACTCTCTCCTCATCTCTAATGGCACTGCAACTTTCTGTAGTCGATACGTT
       G  M  L  H  S  L  L  I  S  N  G  T  A  T  F  C  S  R  Y  V
 601  AAGACCTACAAATATACAGTTGAACGTGAACATGGCTCTTCCATATTTCCTAATGTATTT
       K  T  Y  K  Y  T  V  E  R  E  H  G  S  S  I  F  P  N  V  F
 661  TCTGGTTTTAATGGCCTCACTGCTTCTGTTGCTCGTGGGGCTGTCACTGCTGCTAGGGTT
       S  G  F  N  G  L  T  A  S  V  A  R  G  A  V  T  A  A  R  V
 721  CTCTCTGGTCAGTATAATCCTGTTAATGGAATCGGTCTTGCAAATACAAGTCTTGCTTTC
       L  S  G  Q  Y  N  P  V  N  G  I  G  L  A  N  T  S  L  A  F
 781  TTTGGTGACAAACTTTTTGCTCTTGGAGAATCTGATCTTCCTTATGAAATCAAATTAACT
       F  G  D  K  L  F  A  L  G  E  S  D  L  P  Y  E  I  K  L  T
 841  CCTGATGGTGAAATTGTTACCCTCGGTCGTCACGACTTCGATGGAAAATTGTTTATGTCC
       P  D  G  E  I  V  T  L  G  R  H  D  F  D  G  K  L  F  M  S
 901  ATGACTGCTCATCCTAAAGTAGACCCCAGCACAGGTGAAGCCTTCGCATTCCGATACGGC
       M  T  A  H  P  K  V  D  P  S  T  G  E  A  F  A  F  R  Y  G
 961  CCAATTCCTCCATTTTTGACCTTTTTCCGGTTTAATTCATCCGGTTCGAAACAACCTGAT
       P  I  P  P  F  L  T  F  F  R  F  N  S  S  G  S  K  Q  P  D
1021  GTACCCATTTTCTCCATGACACGACCAGCATTTCTTCATGACTTTGCTATCACCCAAAAA
       V  P  I  F  S  M  T  R  P  A  F  L  H  D  F  A  I  T  Q  K
1081  TTTGCCATATTTACAGAGATACAAATTGGGATGAATCCAATGGAGATGATTGTGGGTAAT
       F  A  I  F  T  E  I  Q  I  G  M  N  P  M  E  M  I  V  G  N
1141  GGGTCACCTGTCGGGGCAGATCCGAGTGTAGTATCTAGGATTGGTGTGATTCCACGGTAT
       G  S  P  V  G  A  D  P  S  V  V  S  R  I  G  V  I  P  R  Y
1201  GCCAAAGACGAATCAGATATGAAATGGTTCGACGTACCCGGGTTTAACATCATCCATGCA
       A  K  D  E  S  D  M  K  W  F  D  V  P  G  F  N  I  I  H  A
1261  GTTAATGCATGGGATGAAGGTGAAGATGAAATAGTTTTAATTGCACCAAATATATTATCA
       V  N  A  W  D  E  G  E  D  E  I  V  L  I  A  P  N  I  L  S
1321  GTAGAACATACTTTAGAAAGAATGGAACTCATTCATGCGCTAATGGAGAAAGTACGTATC
       V  E  H  T  L  E  R  M  E  L  I  H  A  L  M  E  K  V  R  I
1381  AATTTGAGAACGGGTATGGTTTCGCGACACCCGGTGTCAGCAAGAAATCTAGATTTTGGT
       N  L  R  T  G  M  V  S  R  H  P  V  S  A  R  N  L  D  F  G
1441  GTGATTAATCAAAACTTCACAGGGAAAAAGAACAGATTTGTTTATGCAGGAATTGGTGAC
       V  I  N  Q  N  F  T  G  K  K  N  R  F  V  Y  A  G  I  G  D
1501  CCAATGCCTAAGATTTCAGGAGTAGTGAAATTGGATGTGGGAGATGAACATGAGCAGAAT
       P  M  P  K  I  S  G  V  V  K  L  D  V  G  D  E  H  E  Q  N
1561  GATTGTACTGTGGGTTGCAGGATGTTCGGGTCTGGTTGTTATGGTGGTGAACCGTTTTAT
       D  C  T  V  G  C  R  M  F  G  S  G  C  Y  G  G  E  P  F  Y
1621  GTTGCAAAAGAACCCGAGAACCTCGATGCCGATGAAGATGATGGGTATGTAGTGACATAC
       V  A  K  E  P  E  N  L  D  A  D  E  D  D  G  Y  V  V  T  Y
1681  GTACATGATGAGAATTTAGGGGAATCTAAGTTTTTGGTGATGGATGCTAAGTCACCAACG
       V  H  D  E  N  L  G  E  S  K  F  L  V  M  D  A  K  S  P  T
1741  CTTGATGTAGTGGCTGCTGTGACGTTGCCGGGTCGGGTTCCATACGGGTTTCATGGAATC
       L  D  V  V  A  A  V  T  L  P  G  R  V  P  Y  G  F  H  G  I
1801  TTTGTGAGGGAAAGTGAACTCAAGAAGCTGATGTAAAAAAGGGGTGGATGTCATATTGAG
       F  V  R  E  S  E  L  K  K  L  M  *
      TCCTAGTAAATCCTATTATATGTACATGGTAACCACCATACCCATATTTAGGAACA +13
```

**Figure S1M.** The nucleotide sequence and deduced amino acid sequences of CmCCD. An asterisk (*) represents a stop codon.

```
                                          ATAAGCAAAAATCTACACTCCTTTTCACC
  1  ATGCCTACTTCTTCAAGTACTACTTGGATTAATAAACCCTCCATTTCCATAAACAAACCT   60
      M  P  T  S  S  S  T  T  W  I  N  K  P  S  I  S  I  N  K  P
 61  AGAAGAAGAAGTAGTAGTAGTACTCTGTCTTCATCTTTACAGTCTCCTTCTCTACTTCAC  120
      R  R  R  S  S  S  S  T  L  S  S  S  L  Q  S  P  S  L  L  H
121  TTCCCTACCCAACGCCACCAATCTTCTCCTTCCACCACCACCTCCACCTCAGCTGCTATT  180
      F  P  T  Q  R  H  Q  S  S  P  S  T  T  T  S  T  S  A  A  I
181  TCTACCTACACCCATACCCACACTCCAATCACTCTTCCCAAAGAGAAATCCAAAACACTA  240
      S  T  Y  T  H  T  H  T  P  I  T  L  P  K  E  K  S  K  T  L
241  CACCCAATTTCTTCTCCTAATCCTAATCCTAATCCTACGCCAACAACAAAGACAATCCAA  300
      H  P  I  S  S  P  N  P  N  P  N  P  T  P  T  T  K  T  I  Q
301  CACCCACCAAACTGGAATCCTTTCCAAAAAGCTGCATCCATGGCGTTAGATGCTTTTGAA  360
      H  P  P  N  W  N  P  F  Q  K  A  A  S  M  A  L  D  A  F  E
361  ACAGCTTTACTCAACAAAGAACGTCAACACCCACTTCCCAAAACAGCTGACCCTTCCGTT  420
      T  A  L  L  N  K  E  R  Q  H  P  L  P  K  T  A  D  P  S  V
421  CAAATCTCCGGTAACTTCGCTCCCGTTCCAGAACAACCTGTTCGACAGAATCTCCCAGTC  480
      Q  I  S  G  N  F  A  P  V  P  E  Q  P  V  R  Q  N  L  P  V
481  ACCGGCAGAATCCCTTGTTCCATTAGTGGATTTTACGTCCGTAACGGAGCTAATCCTTTA  540
      T  G  R  I  P  C  S  I  S  G  F  Y  V  R  N  G  A  N  P  L
541  CACGAACCA
      H  E  P
```

**Figure S1N.** The nucleotide sequence and deduced amino acid sequences of CmNCED. An asterisk (*) represents a stop codon.

```
-81 1  ATGGAAGCCAGACAAGAAGATGAAGTTAAGAAGAATAAGAAACTGGTTTTCGCCATTAAT
        M  E  A  R  Q  E  D  E  V  K  K  N  K  K  L  V  F  A  I  N
  61   GGAGAGAGATTTGAGCTATCTAAGATCGACCCATCAACTACTGTACTTGAATTCTTACGT
        G  E  R  F  E  L  S  K  I  D  P  S  T  T  V  L  E  F  L  R
 121   ACCCAGACTCGTTTTACTGGAACTAAGCTTAGCTGTGGAGAGGGTGGTTGCGGAGCTTGT
        T  Q  T  R  F  T  G  T  K  L  S  C  G  E  G  G  C  G  A  C
 181   GTCGTTCTTCTGTCCAAGTATGATCCTGTACTTGACAAAGTTGAGGATTTTAAAGTGAGT
        V  V  L  L  S  K  Y  D  P  V  L  D  K  V  E  D  F  K  V  S
 241   TCATGCTTAACTCTTGTTTGCAGTATACATGGATGTTCAATTACGACTACCGAAGGCCTT
        S  C  L  T  L  V  C  S  I  H  G  C  S  I  T  T  T  E  G  L
 301   GGAAATGTCAAAGATGGGTTCCACCCAATCCACAAAAGAATGTCTGGTTTTCATGCTTCT
        G  N  V  K  D  G  F  H  P  I  H  K  R  M  S  G  F  H  A  S
 361   CAGTGTGGGTTCTGTACTCCGGGAATGTGTATGTCTCTCTTCTCTGCTCTTGTTAATGCT
        Q  C  G  F  C  T  P  G  M  C  M  S  L  F  S  A  L  V  N  A
 421   GAGAAAACTCAAAAGGACGATCCTCCTCCTGGATTTTCGAAGCTCACAGTATCAGAAGCT
        E  K  T  Q  K  D  D  P  P  P  G  F  S  K  L  T  V  S  E  A
 481   GAGATGGCTATTTCAGGCAATCTTTGCCGGTGTACAGGTTACAGACCAATTGCCGATGCC
        E  M  A  I  S  G  N  L  C  R  C  T  G  Y  R  P  I  A  D  A
 541   TGCAAGAGTTTTGCAGTAAATGCCGACATGGAAGATCTAGGTCTGAATTCCTTTTGGAAG
        C  K  S  F  A  V  N  A  D  M  E  D  L  G  L  N  S  F  W  K
 601   AAGGGGGACAATGATAAAGCAAGTAAATTACCTTTTTATACCCCACAAAATGAAATTTGC
        K  G  D  N  D  K  A  S  K  L  P  F  Y  T  P  Q  N  E  I  C
 661   ACCTTCCCTGAGTTCTTAAAGACAGAGCTTAAGTCCAAAACACTCTTGGATTCGAAAAAG
        T  F  P  E  F  L  K  T  E  L  K  S  K  T  L  L  D  S  K  K
 721   CATTGTTGGTATAGTCCTGTTACTATTGAGGAACTTAAGGGCCTAATGGAATGCTTAGAA
        H  C  W  Y  S  P  V  T  I  E  E  L  K  G  L  M  E  C  L  E
 781   AATGAAAATTGTAACGCGCTTAAACTAGTTGTTGGTAATACGGGTACAGGCTATTACAAG
        N  E  N  C  N  A  L  K  L  V  V  G  N  T  G  T  G  Y  Y  K
 841   GAGCTAAAACAGTACGACAAGTATATTGATCTAAGACATATTCCTGAGCTCTCAGTGATC
        E  L  K  Q  Y  D  K  Y  I  D  L  R  H  I  P  E  L  S  V  I
 901   AGAAGTGATAGCAGTGGTGTTGAAATTGGAGCTGCTGTGACTATCTCTAACACTATCCAT
        R  S  D  S  S  G  V  E  I  G  A  A  V  T  I  S  N  T  I  H
 961   GCTTTGATGGAAGAAAGAAAAGATGGGTATAACTCAACCATAAATCTTGTGTTCAGGAAA
        A  L  M  E  E  R  K  D  G  Y  N  S  T  I  N  L  V  F  R  K
1021   ATTTCTGACCATATGAACAAGGTTGCGTCAGAGTTTGTTCGCAATACTGCGAGTTTGGGG
        I  S  D  H  M  N  K  V  A  S  E  F  V  R  N  T  A  S  L  G
1081   GGTAATTTGGTCATGGCTCAGAGGAATCACTTCCCCTCAGATATTGCTACTATACTTCTT
        G  N  L  V  M  A  Q  R  N  H  F  P  S  D  I  A  T  I  L  L
1141   GCTGCTGGTTCATCGCTAGATATACTTAAAGGCTCCAAAAGACTAAAGCTTACATTGGAG
        A  A  G  S  S  L  D  I  L  K  G  S  K  R  L  K  L  T  L  E
1201   GAATTCTTAGAGAGTCCTCCTTGTGATACTAAAACTGTACTCTTGAGTATTAGAATCCCA
        E  F  L  E  S  P  P  C  D  T  K  T  V  L  L  S  I  R  I  P
1261   AGTTGGGACCCTGTAAGAAGCTTTTCTTCTGCAAATGGAACTGGATTGATGTTTGAGACC
        S  W  D  P  V  R  S  F  S  S  A  N  G  T  G  L  M  F  E  T
1321   TACCGAGCTGCACCACGACCCCTCGGGAATGCCTTGGCTTACCTTAATGCTGCTTTCTTG
        Y  R  A  A  P  R  P  L  G  N  A  L  A  Y  L  N  A  A  F  L
1381   GCTGAGGTTTCTACCTCTAAAACATCTGGTTGTATTGTTTTGGAGAAACTTCATTTGGCC
        A  E  V  S  T  S  K  T  S  G  C  I  V  L  E  K  L  H  L  A
1441   TTTGGTGCTTATGGGACAAAACATGCAATAAGGGCAAGAAATGTTGAGGAACTTTTGATT
        F  G  A  Y  G  T  K  H  A  I  R  A  R  N  V  E  E  L  L  I
1501   GGGAGATCTTTTAATAAAGGTGTTCTGTTTGAAGCCATTAAATTACTTAGAGCTACAATC
        G  R  S  F  N  K  G  V  L  F  E  A  I  K  L  L  R  A  T  I
1561   ATACCTGAAGGGGGCACTTCAAGTCCTGCTTACAGGTCTAGCTTGGCTGTTAGTTTCCTC
        I  P  E  G  G  T  S  S  P  A  Y  R  S  S  L  A  V  S  F  L
1621   TTTGATTTTCTTCACCGTCTTACAGAAATTAGTGCTGCAGTACCTAGTAATGGTTTAACT
        F  D  F  L  H  R  L  T  E  I  S  A  A  V  P  S  N  G  L  T
1681   GGGGGTGTTCATGCTCCAGATTGTAAGGCGTCCAAACCCAACAAATGGTTAGATCAGTGT
        G  G  V  H  A  P  D  C  K  A  S  K  P  N  K  W  L  D  Q  C
1741   TCTCATATGGATAACCCAGGTCTACTCTCTTCAGGAAAGCAGGTGATCGAAGTTAGCAGG
        S  H  M  D  N  P  G  L  L  S  S  G  K  Q  V  I  E  V  S  R
1801   GAGTATCATCCAGTTGGTGAGCCTACTAAAAAAGTTGGAGCCGAGATCCAAGCTTCTGGT
        E  Y  H  P  V  G  E  P  T  K  K  V  G  A  E  I  Q  A  S  G
1861   GAGGCTGTTTATGTGGATGACATTCCATCCCCCAAGGACTGCCTTCATGGAGCATTCATA
        E  A  V  Y  V  D  D  I  P  S  P  K  D  C  L  H  G  A  F  I
1921   TACAGCAAAAGGCCCTTGGCATGGATAAAGAGTGTCGAGCTCGTGTCTCCTTTACCATCA
        Y  S  K  R  P  L  A  W  I  K  S  V  E  L  V  S  P  L  P  S
1981   GCTCGAGTTTTTAGAGTTATTTCTGTCAAAGATATCCCTAAAGGCGGGGAAAATGTTGGA
        A  R  V  F  R  V  I  S  V  K  D  I  P  K  G  G  E  N  V  G
2041   AGTAGGACCATGTTTGGTACTGAACCTCTATTTGCTGATCAACTTACTCAGTATGCAGGC
        S  R  T  M  F  G  T  E  P  L  F  A  D  Q  L  T  Q  Y  A  G
2101   CAGATGCTCGGCTTTGTGGTTGCAGATACACAGAAACATGCAGATATGGCTGCTGATGGT
        Q  M  L  G  F  V  V  A  D  T  Q  K  H  A  D  M  A  A  D  G
2161   GCAGTGGTAGAATATGACACTGGAAATCTAGAACCACCCATTTTATCAGTAGAAGAGGCT
        A  V  V  E  Y  D  T  G  N  L  E  P  P  I  L  S  V  E  E  A
2221   GTCAATAGATGCAGCTTCTTTGACGTGCCTCCATTCCTTTCCCCGAAACAGGTAGGAGAT
        V  N  R  C  S  F  F  D  V  P  P  F  L  S  P  K  Q  V  G  D
2281   TTCTCAGAAGGGATGCGCGAAGCAGATCACAAAATTCTCTCAGCAGAGATCAAACTTGGT
        F  S  E  G  M  R  E  A  D  H  K  I  L  S  A  E  I  K  L  G
2341   TCTCAGTACTATTTCTATATGGAGACACAAACTGCCCTTGCCGTACCAGATGAAGACAAC
        S  Q  Y  Y  F  Y  M  E  T  Q  T  A  L  A  V  P  D  E  D  N
2401   TGCATGGTGGTTTACAGCTCAACTCAGTGCCCTGAGAATGCACAGATTGTGATTGCTAAA
        C  M  V  V  Y  S  S  T  Q  C  P  E  N  A  Q  I  V  I  A  K
2461   TGCCTTGGTCTTCCTGAACATAACGTCCGTGTGATTACAAGAAGAGTTGGAGGAGGATTC
        C  L  G  L  P  E  H  N  V  R  V  I  T  R  R  V  G  G  G  F
2521   GGTGGAAAGGCCTTAAAAGCAATGCCTGTTGCTACAGCATGCGCAGTTGCTGCACACAAA
        G  G  K  A  L  K  A  M  P  V  A  T  A  C  A  V  A  A  H  K
2581   TTACGTCATCCCGTTCGAGTTTACCTCAGTCGTAAAACGGATATGATAATGGCAGGAGGA
        L  R  H  P  V  R  V  Y  L  S  R  K  T  D  M  I  M  A  G  G
2641   AGACATCCTATGAAAATAAAGTACAGTGTCGGTTTCAAGTCTGACGGAAAGATCACTGGG
        R  H  P  M  K  I  K  Y  S  V  G  F  K  S  D  G  K  I  T  G
2701   CTACACCTGGATATACTAATCAATGCAGGAATATCTGAAGATATTAGTCCAATCATGCCA
        L  H  L  D  I  L  I  N  A  G  I  S  E  D  I  S  P  I  M  P
2761   AATAATATGTTGGGTGCACTGAAGAAGTACAACTGGGGAGCGTTGTCTTTTGATATAAAG
        N  N  M  L  G  A  L  K  K  Y  N  W  G  A  L  S  F  D  I  K
2821   CTCTGTAAGACAAACCTTTCAAGTAAGTCAGCCATGCGAGCACCGGGAGAGGTACAGGCA
        L  C  K  T  N  L  S  S  K  S  A  M  R  A  P  G  E  V  Q  A
2881   TCCTTTATTGCAGAGGCTGTGATTGAGCATGTAGCATCATTTCTTTCTATGGAAGTTGAT
        S  F  I  A  E  A  V  I  E  H  V  A  S  F  L  S  M  E  V  D
2941   TATGTCAGGGAAAGAAATATACATACATACGAGAGCCTCAAATTATTTTATCAGGATACT
        Y  V  R  E  R  N  I  H  T  Y  E  S  L  K  L  F  Y  Q  D  T
3001   GCTGGGGAACCTATTGAGTATACTTTGCCTTTGGTTTTAGATAAATTGTCCAAATCTTCT
        A  G  E  P  I  E  Y  T  L  P  L  V  L  D  K  L  S  K  S  S
3061   AACTTCTTCCAAAGGGTAGAAGTAATAAAACAATTCAATAGCTATAATAAATGGAGGAAA
        N  F  F  Q  R  V  E  V  I  K  Q  F  N  S  Y  N  K  W  R  K
3121   AGAGGAATTTCTCGGGTGCCCATCTTTCATCAAGTAATGCTTAGACCAACTCCAGGTAAA
        R  G  I  S  R  V  P  I  F  H  Q  V  M  L  R  P  T  P  G  K
3181   GTAAGCATTCTCAACGATGGGTCGGTGGTGGTTGAAGTTGGAGGCATTGAGCTGGGGCAA
        V  S  I  L  N  D  G  S  V  V  V  E  V  G  G  I  E  L  G  Q
3241   GGGCTTTGGACAAAGGTGAAGCAGATGGCGGCATTTGCTCTCAGTCCTGTAGAATGTGAT
        G  L  W  T  K  V  K  Q  M  A  A  F  A  L  S  P  V  E  C  D
3301   GGAAGTGAAAATCTCTTGGAGCGGGTGCGTGTCATTCAGGCTGATACCTTGAGCTTAGTG
        G  S  E  N  L  L  E  R  V  R  V  I  Q  A  D  T  L  S  L  V
3361   CAAGGAGGATTTACCGCAGGGAGCACAACCTCAGAATCAAGTTGCGAAGCTGTCCGTCTT
        Q  G  G  F  T  A  G  S  T  T  S  E  S  S  C  E  A  V  R  L
3421   TGCTGTAATGCTTTGGTTGAAAGACTTACCATACTTAAGGAAAGGTTGCAAGAGCAGATG
        C  C  N  A  L  V  E  R  L  T  I  L  K  E  R  L  Q  E  Q  M
3481   GGTCCAGTTTCATGGGATACTTTAATAATCCAGGCACATCTCCAATCTGTCAACTTATCT
        G  P  V  S  W  D  T  L  I  I  Q  A  H  L  Q  S  V  N  L  S
3541   GCAAGTGTTTTTTATGTTCCTGATTTAAGTTCAACGCATTATCTGAACTACGGTGCCGCT
        A  S  V  F  Y  V  P  D  L  S  S  T  H  Y  L  N  Y  G  A  A
3601   GTGAGTGAGGTGGAGATTGATCTTCTCACAGGAGCAACCACAATATTGCGAACAGATATT
        V  S  E  V  E  I  D  L  L  T  G  A  T  T  I  L  R  T  D  I
3661   GTCTATGATTGTGGACAGAGCCTGAACCCTGCCGTAGATCTGGGACAGATTGAAGGCGCT
        V  Y  D  C  G  Q  S  L  N  P  A  V  D  L  G  Q  I  E  G  A
3721   TTTGTTCAAGGAGTTGGGTTTTTTATGCTGGAAGAGTACCTGACAAACTCCGATGGATTG
        F  V  Q  G  V  G  F  F  M  L  E  E  Y  L  T  N  S  D  G  L
3781   GTAGTATCTGAAGGAACATGGACCTACAAGATTCCAACAATAGACACCATCCCGAGACAG
        V  V  S  E  G  T  W  T  Y  K  I  P  T  I  D  T  I  P  R  Q
3841   TTCAATGTTGAGCTACTGAACAGCGGACATCATCAAAAACGTGTGCTCTCCTCTAAAGCT
        F  N  V  E  L  L  N  S  G  H  H  Q  K  R  V  L  S  S  K  A
3901   TCTGGGGAACCACCGTTACTCATGGCAGCTTCAGTTCACTGTGCAACAAGAGACGCTATT
        S  G  E  P  P  L  L  M  A  A  S  V  H  C  A  T  R  D  A  I
3961   AAAGAAGCAAGAAAACAGGTCCTTTCTTGGAGTGGGTCAGAACTCGATGCATCATCTTAT
        K  E  A  R  K  Q  V  L  S  W  S  G  S  E  L  D  A  S  S  Y
4021   TCCACATTCCAATTGGATGTCCCTGCAACAATGCCTGTAGTGAAGGAGCTATGTGGACTC
        S  T  F  Q  L  D  V  P  A  T  M  P  V  V  K  E  L  C  G  L
4081   GATAGCGTTGAGCGGTATTTAGAAAGTTTACTGCTTTCCCATTAATGAAACTGT +155
        D  S  V  E  R  Y  L  E  S  L  L  L  S  H  *
```

**Figure S1O.** The nucleotide sequence and deduced amino acid sequences of CmAO. An asterisk (*) represents a stop codon.

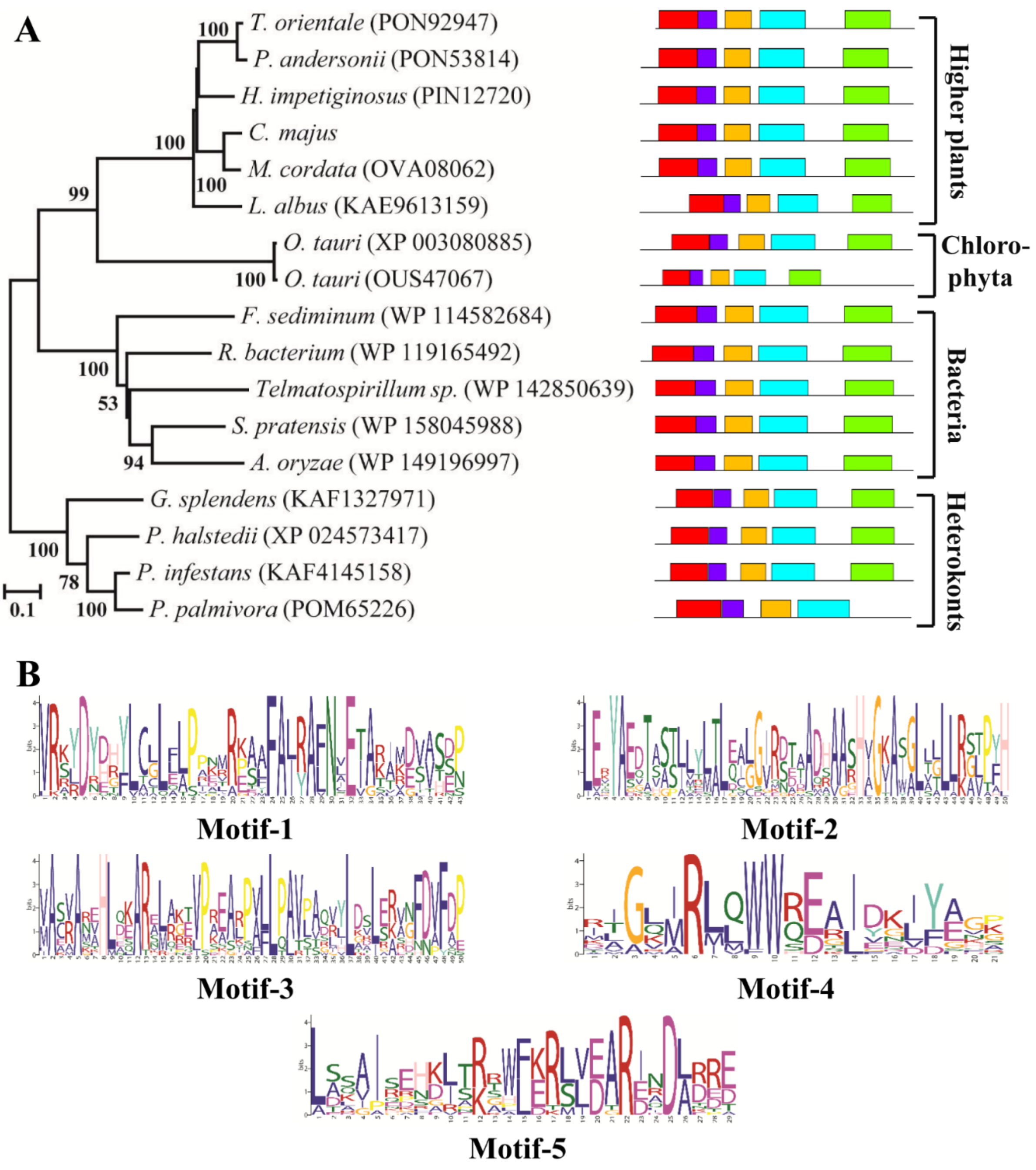


**Figure S2A:** Phylogeny of deduced CmPSY amino acid sequences along with other PSY sequences. The tree was constructed using MEGA 7 [116] on the basis of the Neighbor–Joining method [117]. Bootstrap values on the nodes indicate the number of times each group occurred within 1000 replicates [118]. On the right side, the analysis of conserved protein motifs using MEME software is presented, showing motifs and are ordered in colored boxes corresponding to PSY sequence in the phylogenetic tree. Other protein sequence obtained from NCBI database as well as their accession numbers are provided in the bracket. (B) Conserved protein motif 1-5 present in the variable region of CmPSY gene. The height of the letters denotes the degree of conservation.

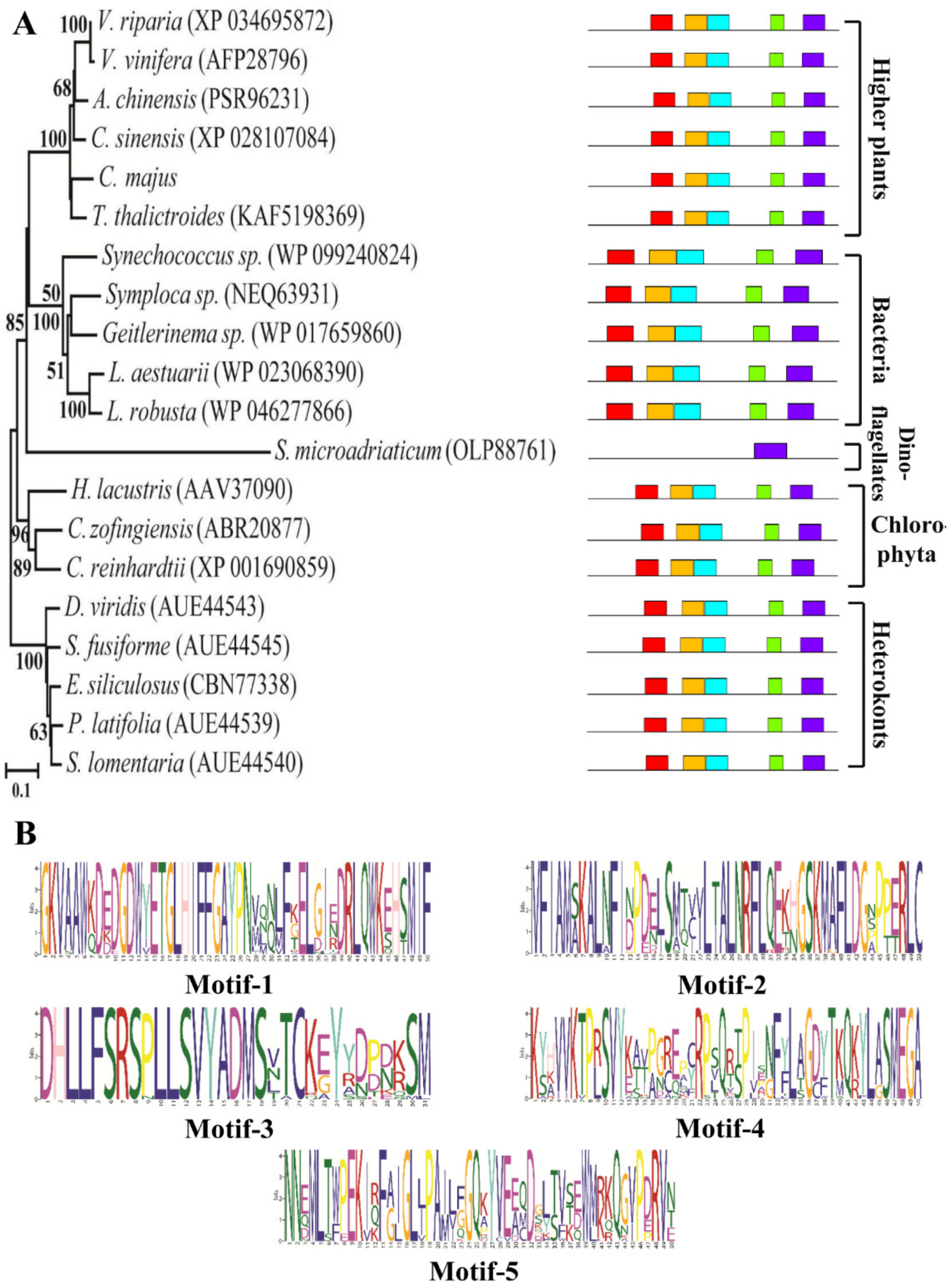


**Figure S2B:** Phylogeny of deduced CmPDS amino acid sequences along with other PDS sequences. The tree was constructed using MEGA 7 [116] on the basis of the Neighbor–Joining method [117]. Bootstrap values on the nodes indicate the number of times each group occurred within 1000 replicates [118]. On the right side, the analysis of conserved protein motifs using MEME software is presented, showing motifs and are ordered in colored boxes corresponding to PDS sequence in the phylogenetic tree. Other protein sequence obtained from NCBI database as well as their accession numbers are provided in the bracket. (B) Conserved protein motif 1-5 present in the variable region of CmPDS gene. The height of the letters denotes the degree of conservation.

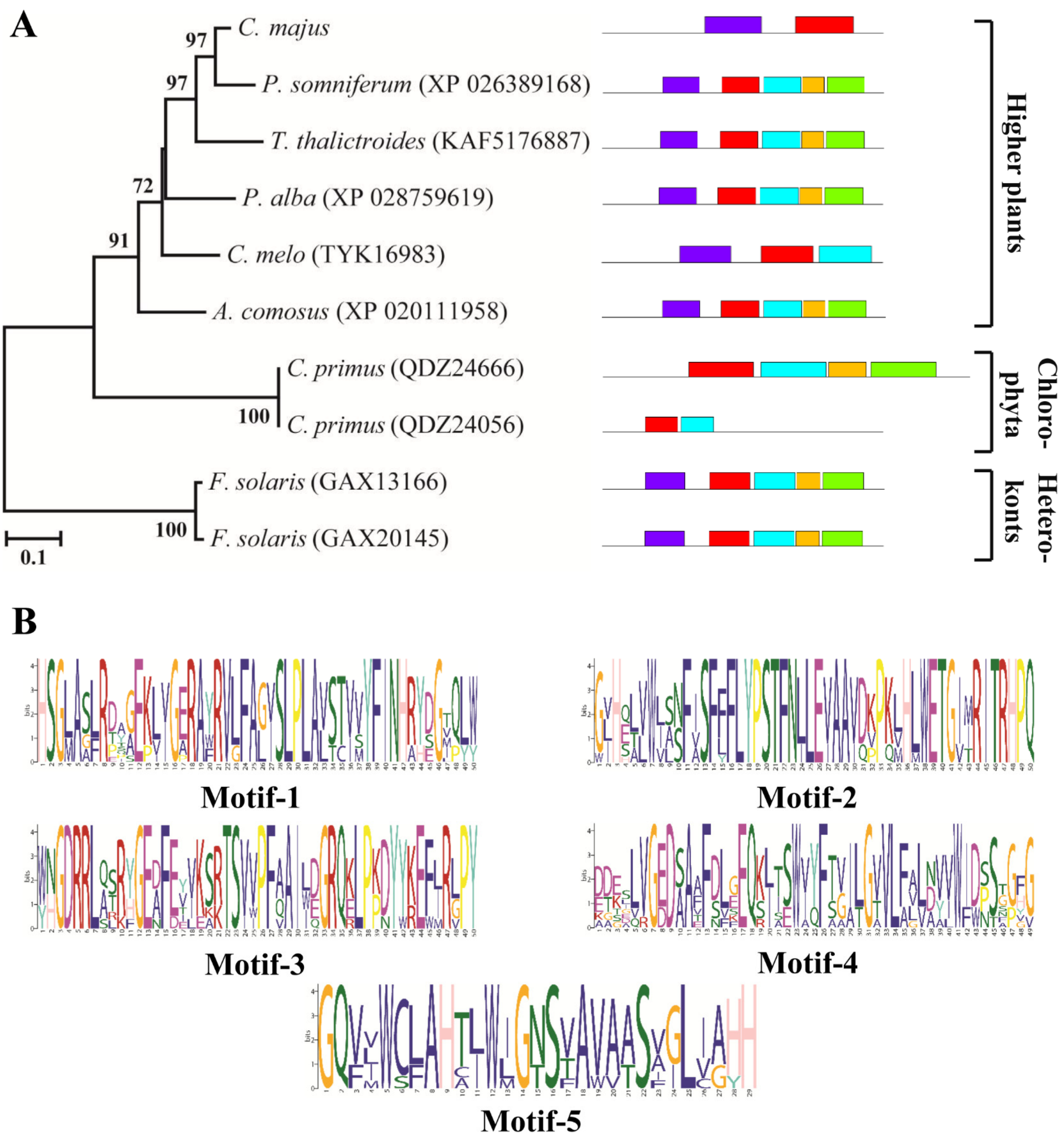


**Figure S2C:** Phylogeny of deduced CmZ-ISO amino acid sequences along with other Z-ISO sequences. The tree was constructed using MEGA 7 [116] on the basis of the Neighbor–Joining method [117]. Bootstrap values on the nodes indicate the number of times each group occurred within 1000 replicates [118]. On the right side, the analysis of conserved protein motifs using MEME software is presented, showing motifs and are ordered in colored boxes corresponding to Z-ISO sequence in the phylogenetic tree. Other protein sequence obtained from NCBI database as well as their accession numbers are provided in the bracket. (B) Conserved protein motif 1-5 present in the variable region of CmZ-ISO gene. The height of the letters denotes the degree of conservation.

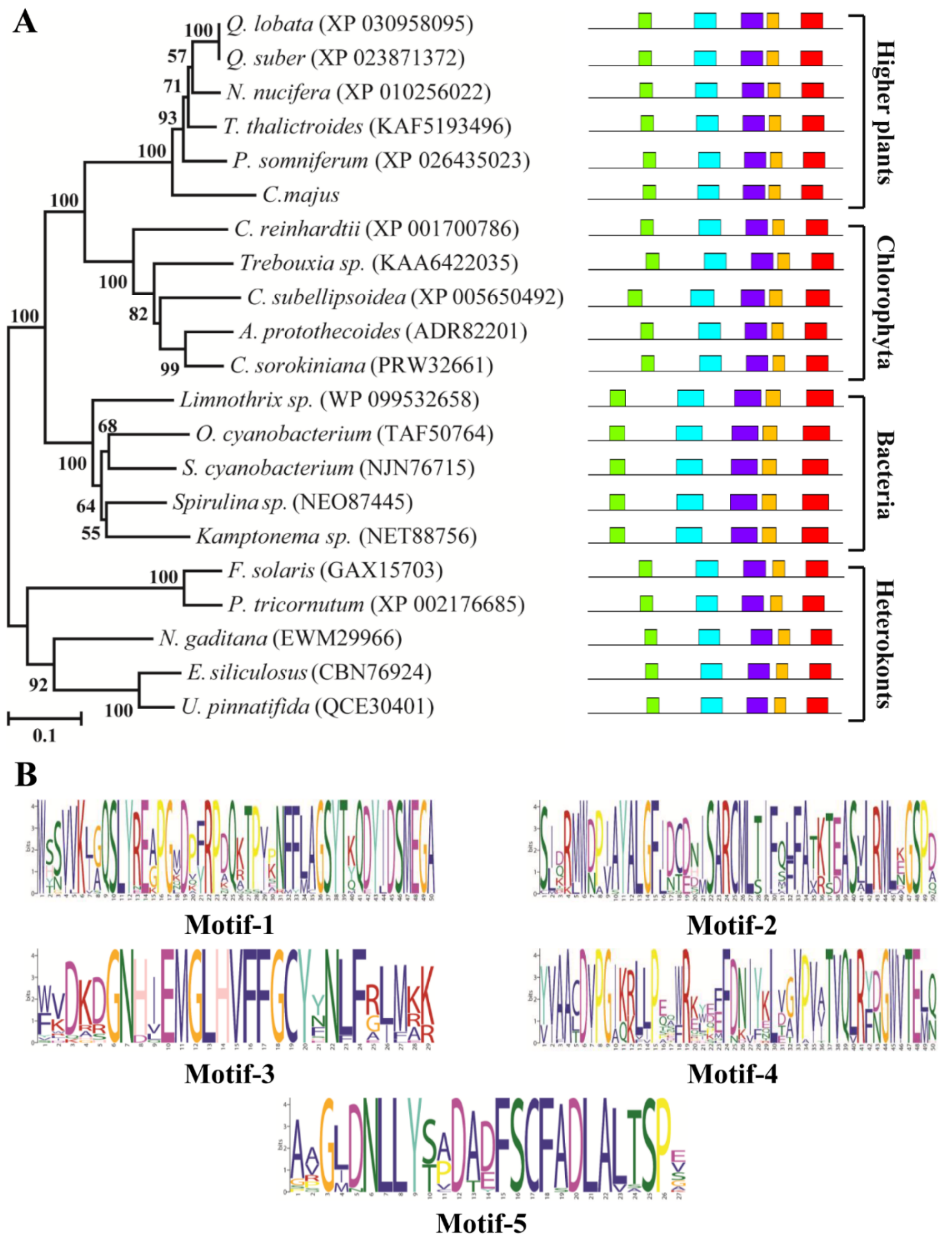


**Figure S2D:** Phylogeny of deduced CmZDS amino acid sequences along with other ZDS sequences. The tree was constructed using MEGA 7 [116] on the basis of the Neighbor–Joining method [117]. Bootstrap values on the nodes indicate the number of times each group occurred within 1000 replicates [118]. On the right side, the analysis of conserved protein motifs using MEME software is presented, showing motifs and are ordered in colored boxes corresponding to ZDS sequence in the phylogenetic tree. Other protein sequence obtained from NCBI database as well as their accession numbers are provided in the bracket. (B) Conserved protein motif 1-5 present in the variable region of CmZDS gene. The height of the letters denotes the degree of conservation.

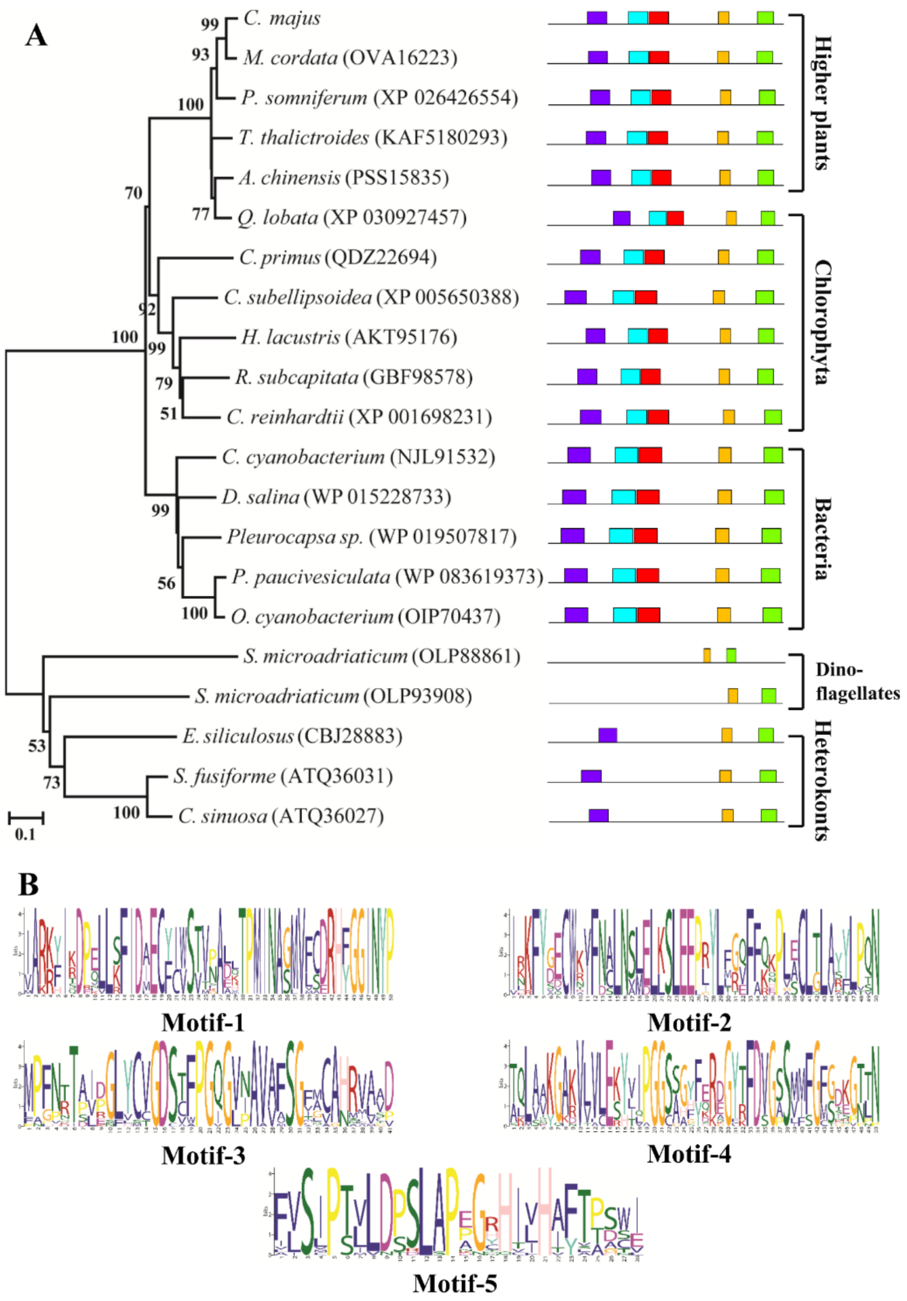


**Figure S2E:** Phylogeny of deduced CmCrtISO amino acid sequences along with other CrtISO sequences. The tree was constructed using MEGA 7 [116] on the basis of the Neighbor–Joining method [117]. Bootstrap values on the nodes indicate the number of times each group occurred within 1000 replicates [118]. On the right side, the analysis of conserved protein motifs using MEME software is presented, showing motifs and are ordered in colored boxes corresponding to CrtISO sequence in the phylogenetic tree. Other protein sequence obtained from NCBI database as well as their accession numbers are provided in the bracket. (B) Conserved protein motif 1-5 present in the variable region of CmCrtISO gene. The height of the letters denotes the degree of conservation.

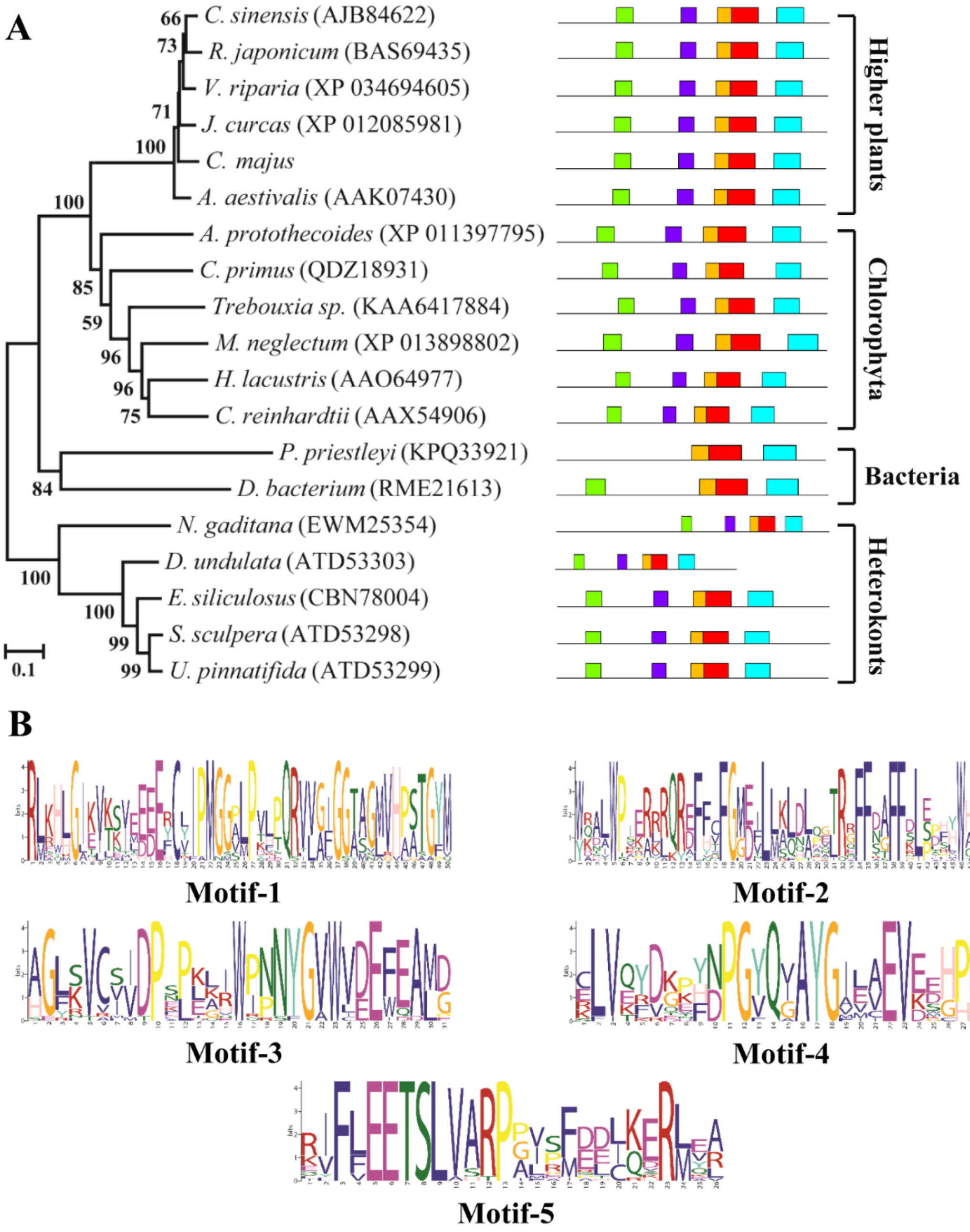


**Figure S2F:** Phylogeny of deduced CmLCYB amino acid sequences along with other LCYB sequences. The tree was constructed using MEGA 7 [116] on the basis of the Neighbor–Joining method [117]. Bootstrap values on the nodes indicate the number of times each group occurred within 1000 replicates [118]. On the right side, the analysis of conserved protein motifs using MEME software is presented, showing motifs and are ordered in colored boxes corresponding to LCYB sequence in the phylogenetic tree. Other protein sequence obtained from NCBI database as well as their accession numbers are provided in the bracket. (B) Conserved protein motif 1-5 present in the variable region of CmLCYB gene. The height of the letters denotes the degree of conservation.

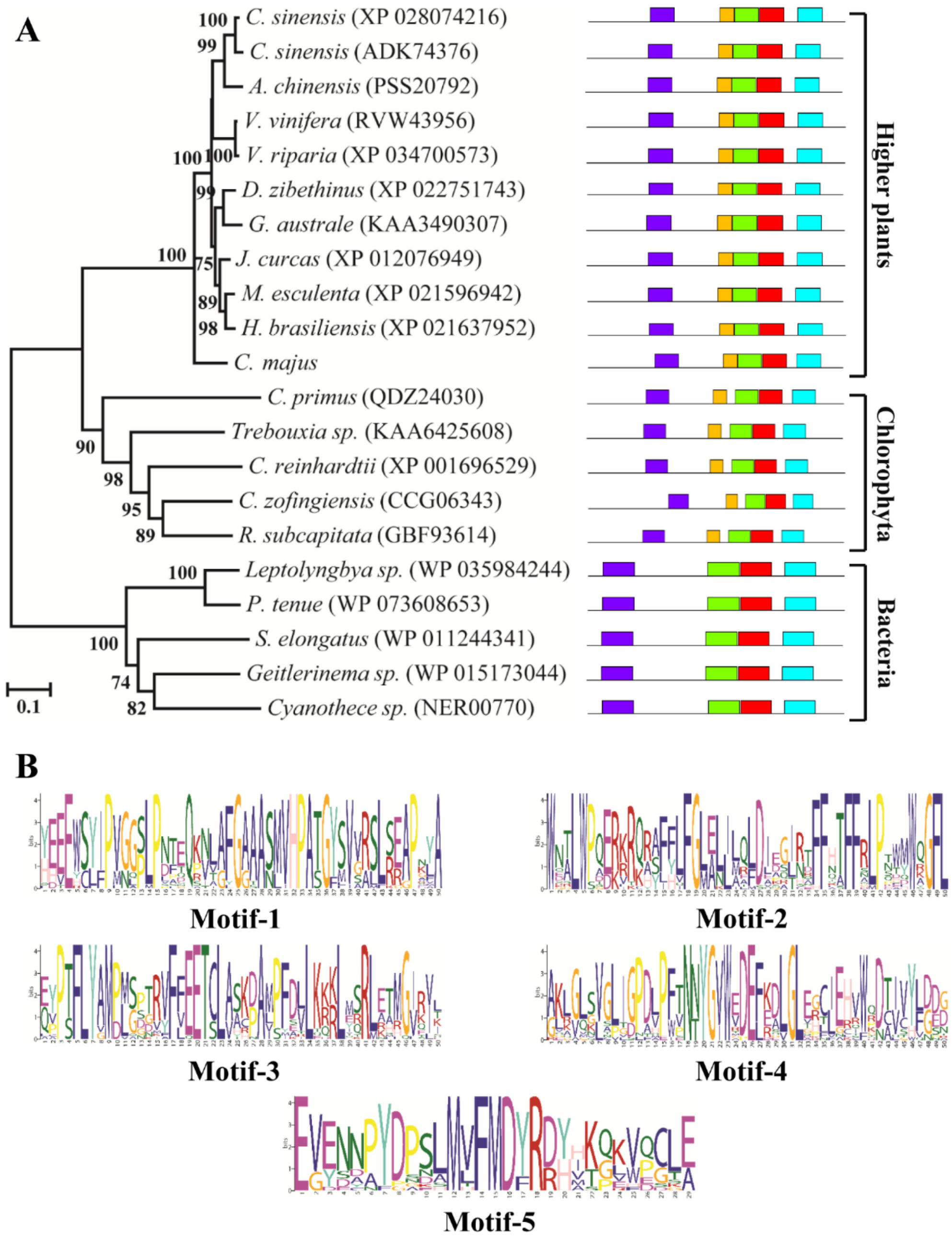


**Figure S2G:** Phylogeny of deduced CmLCYE amino acid sequences along with other LCYE sequences. The tree was constructed using MEGA 7 [116] on the basis of the Neighbor–Joining method [117]. Bootstrap values on the nodes indicate the number of times each group occurred within 1000 replicates [118]. On the right side, the analysis of conserved protein motifs using MEME software is presented, showing motifs and are ordered in colored boxes corresponding to LCYE sequence in the phylogenetic tree. Other protein sequence obtained from NCBI database as well as their accession numbers are provided in the bracket. (B) Conserved protein motif 1-5 present in the variable region of CmLCYE gene. The height of the letters denotes the degree of conservation.

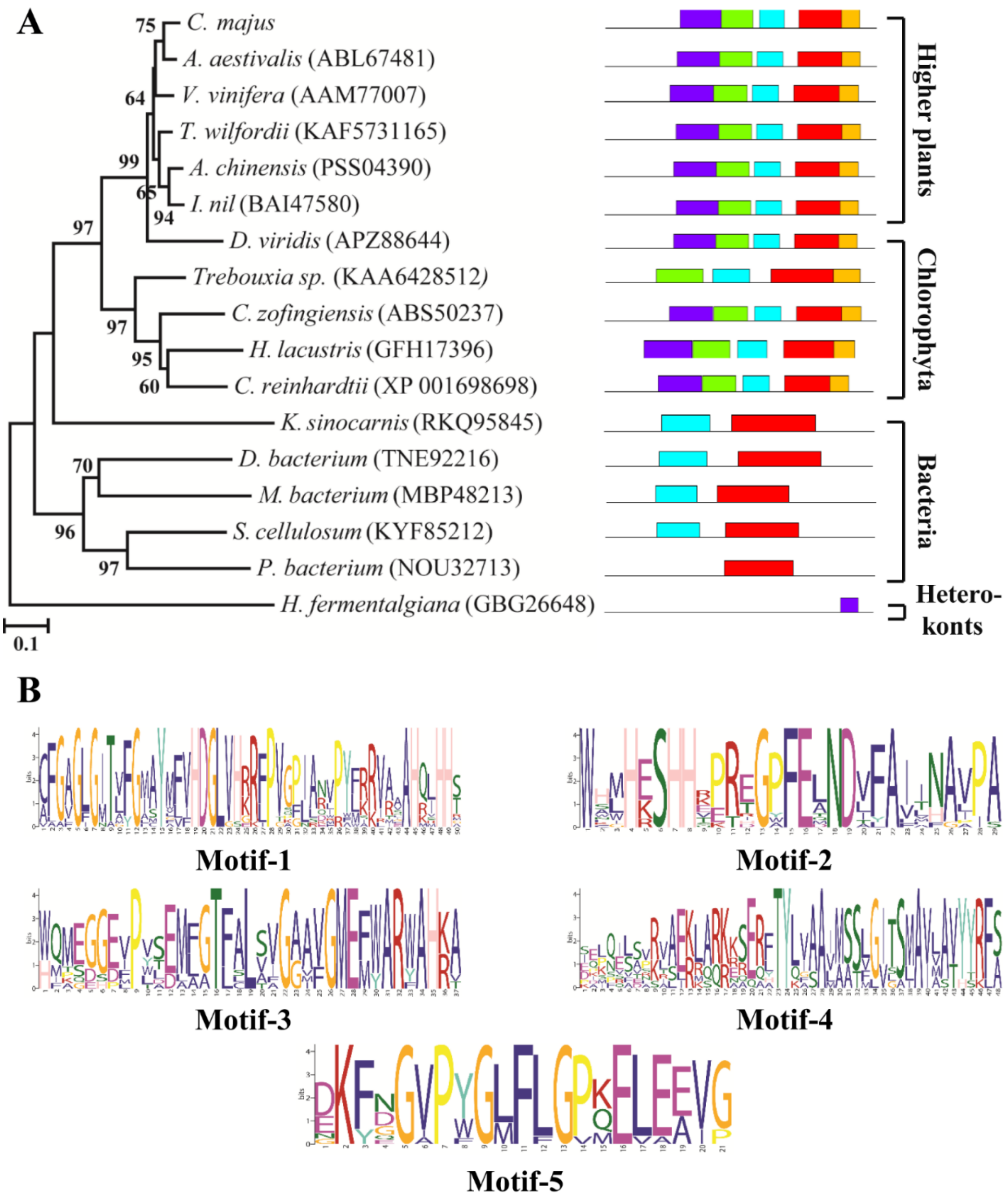


**Figure S2H:** Phylogeny of deduced CmCHXB amino acid sequences along with other CHXB sequences. The tree was constructed using MEGA 7 [116] on the basis of the Neighbor–Joining method [117]. Bootstrap values on the nodes indicate the number of times each group occurred within 1000 replicates [118]. On the right side, the analysis of conserved protein motifs using MEME software is presented, showing motifs and are ordered in colored boxes corresponding to CHXB sequence in the phylogenetic tree. Other protein sequence obtained from NCBI database as well as their accession numbers are provided in the bracket. (B) Conserved protein motif 1-5 present in the variable region of CmCHXB gene. The height of the letters denotes the degree of conservation.

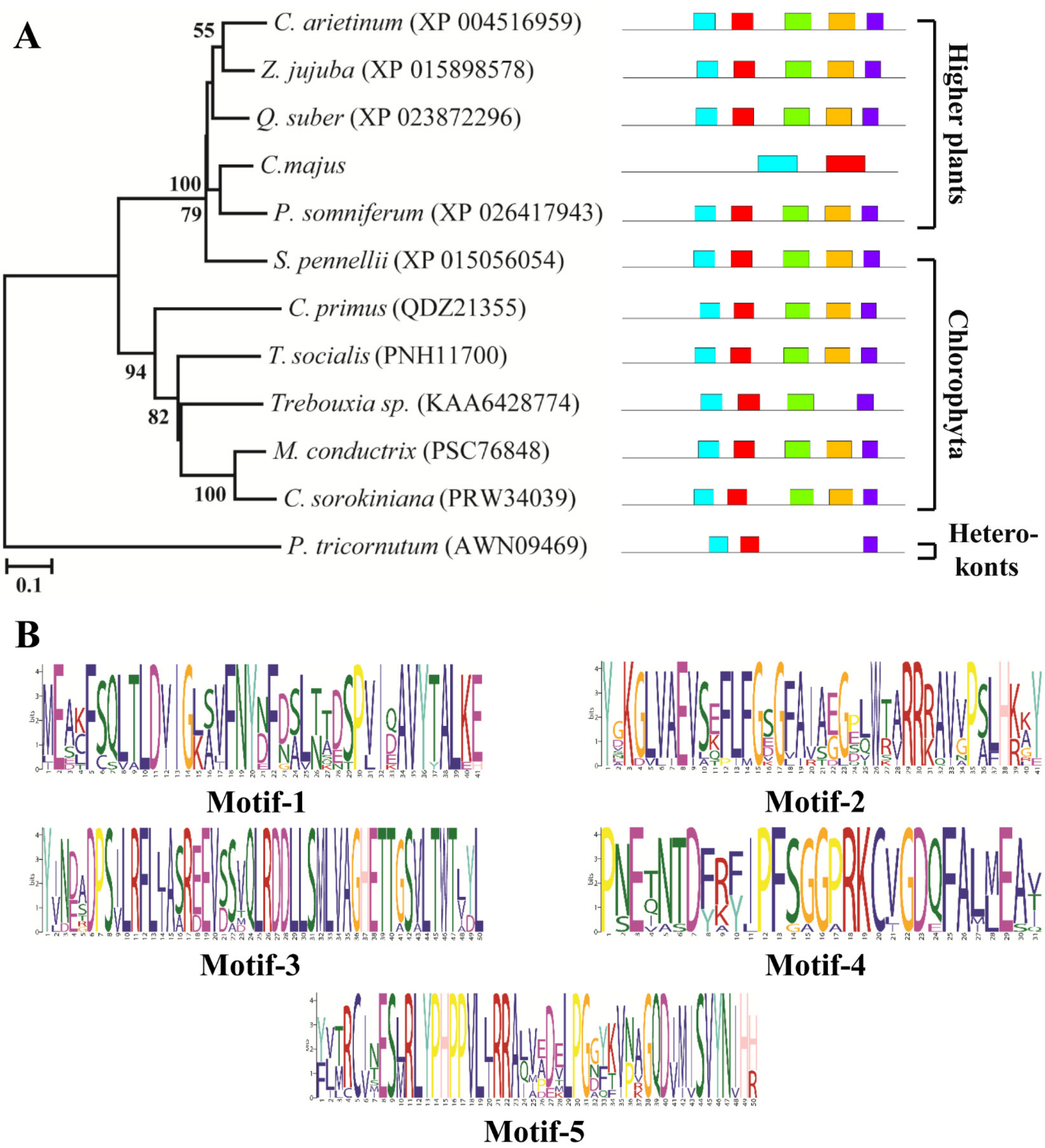


**Figure S2I:** Phylogeny of deduced CmCHXE amino acid sequences along with other CHXE sequences. The tree was constructed using MEGA 7 [116] on the basis of the Neighbor–Joining method [117]. Bootstrap values on the nodes indicate the number of times each group occurred within 1000 replicates [118]. On the right side, the analysis of conserved protein motifs using MEME software is presented, showing motifs and are ordered in colored boxes corresponding to CHXE sequence in the phylogenetic tree. Other protein sequence obtained from NCBI database as well as their accession numbers are provided in the bracket. (B) Conserved protein motif 1-5 present in the variable region of CmCHXE gene. The height of the letters denotes the degree of conservation.

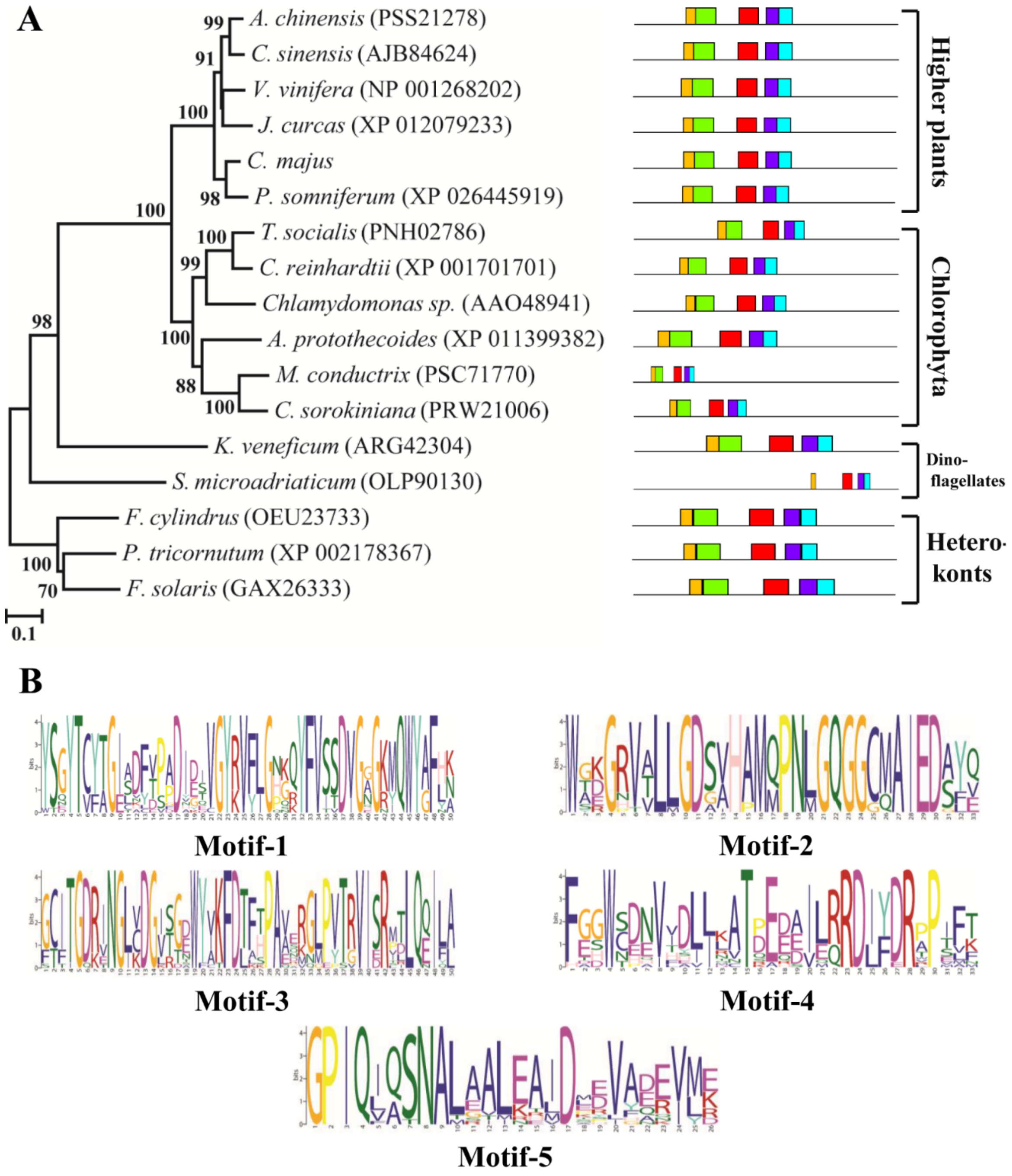


**Figure S2J:** Phylogeny of deduced CmZEP amino acid sequences along with other ZEP sequences. The tree was constructed using MEGA 7 [116] on the basis of the Neighbor–Joining method [117]. Bootstrap values on the nodes indicate the number of times each group occurred within 1000 replicates [118]. On the right side, the analysis of conserved protein motifs using MEME software is presented, showing motifs and are ordered in colored boxes corresponding to ZEP sequence in the phylogenetic tree. Other protein sequence obtained from NCBI database as well as their accession numbers are provided in the bracket. (B) Conserved protein motif 1-5 present in the variable region of CmZEP gene. The height of the letters denotes the degree of conservation.

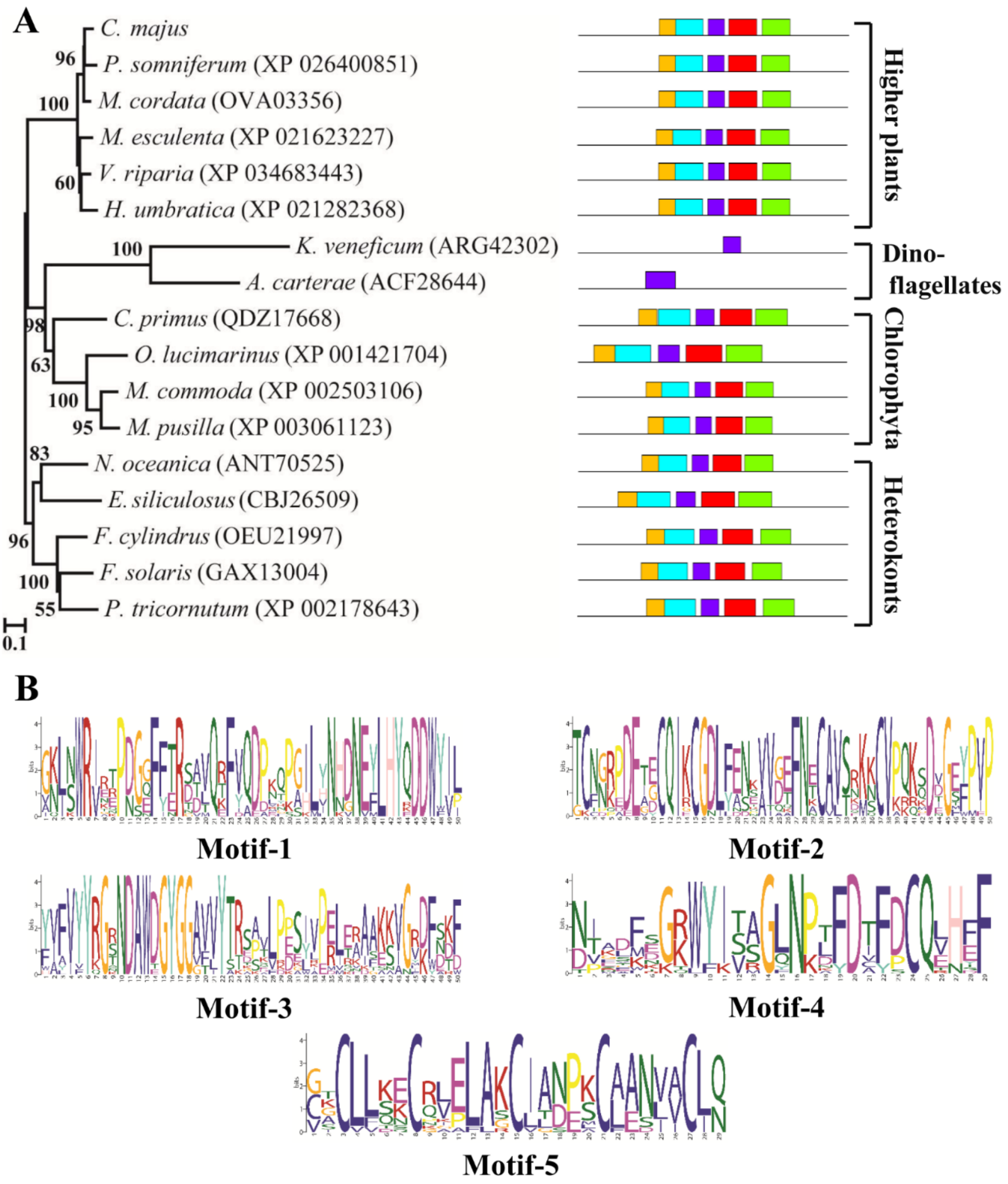


**Figure S2K:** Phylogeny of deduced CmVDE amino acid sequences along with other VDE sequences. The tree was constructed using MEGA 7 [116] on the basis of the Neighbor–Joining method [117]. Bootstrap values on the nodes indicate the number of times each group occurred within 1000 replicates [118]. On the right side, the analysis of conserved protein motifs using MEME software is presented, showing motifs and are ordered in colored boxes corresponding to VDE sequence in the phylogenetic tree. Other protein sequence obtained from NCBI database as well as their accession numbers are provided in the bracket. (B) Conserved protein motif 1-5 present in the variable region of CmVDE gene. The height of the letters denotes the degree of conservation.

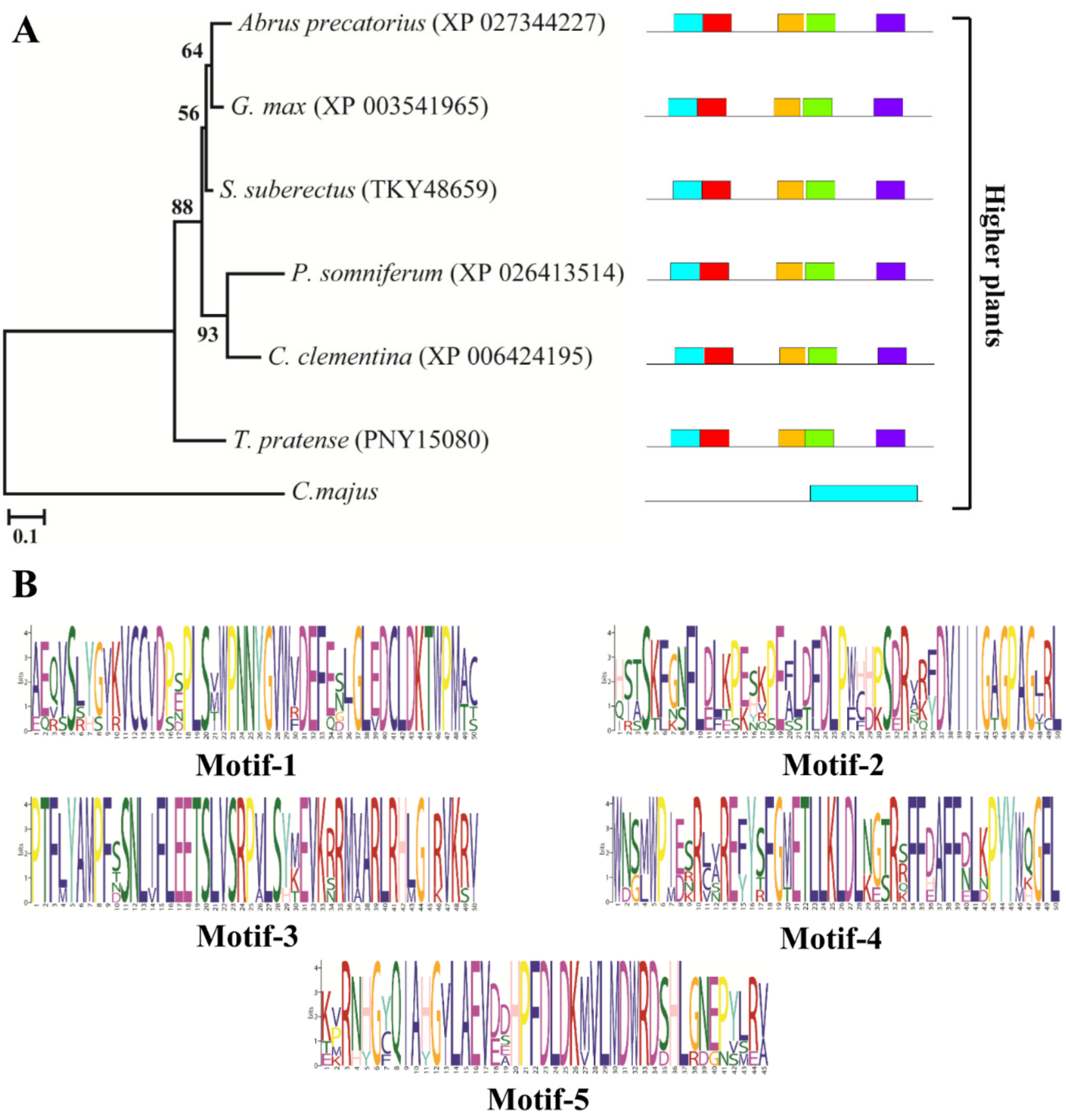


**Figure S2L:** Phylogeny of deduced CmCCS amino acid sequences along with other CCS sequences. The tree was constructed using MEGA 7 [116] on the basis of the Neighbor–Joining method [117]. Bootstrap values on the nodes indicate the number of times each group occurred within 1000 replicates [118]. On the right side, the analysis of conserved protein motifs using MEME software is presented, showing motifs and are ordered in colored boxes corresponding to CCS sequence in the phylogenetic tree. Other protein sequence obtained from NCBI database as well as their accession numbers are provided in the bracket. (B) Conserved protein motif 1-5 present in the variable region of CmCCS gene. The height of the letters denotes the degree of conservation.

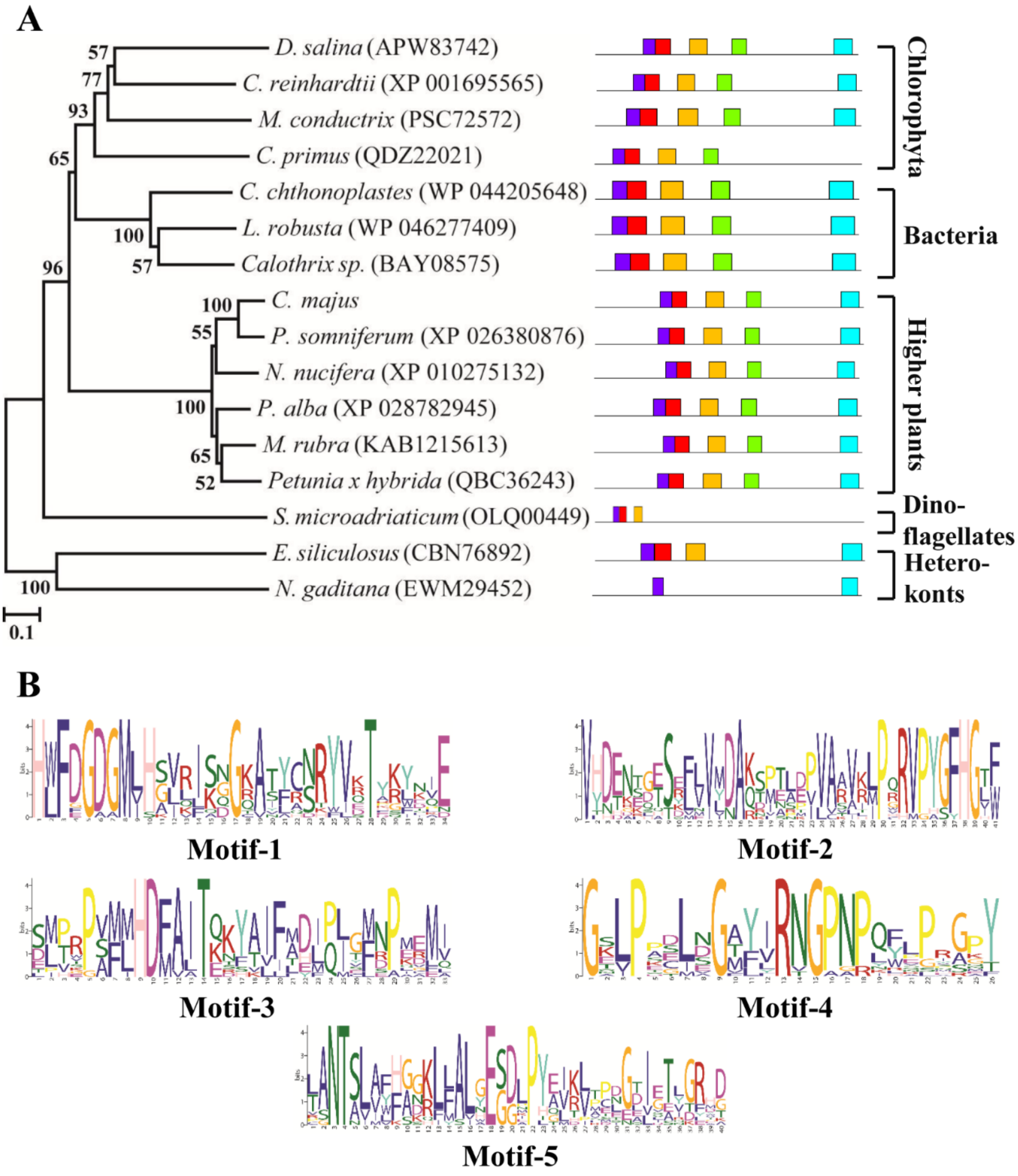


**Figure S2M:** Phylogeny of deduced CmCCD amino acid sequences along with other CCD sequences. The tree was constructed using MEGA 7 [116] on the basis of the Neighbor–Joining method [117]. Bootstrap values on the nodes indicate the number of times each group occurred within 1000 replicates [118]. On the right side, the analysis of conserved protein motifs using MEME software is presented, showing motifs and are ordered in colored boxes corresponding to CCD sequence in the phylogenetic tree. Other protein sequence obtained from NCBI database as well as their accession numbers are provided in the bracket. (B) Conserved protein motif 1-5 present in the variable region of CmCCD gene. The height of the letters denotes the degree of conservation.

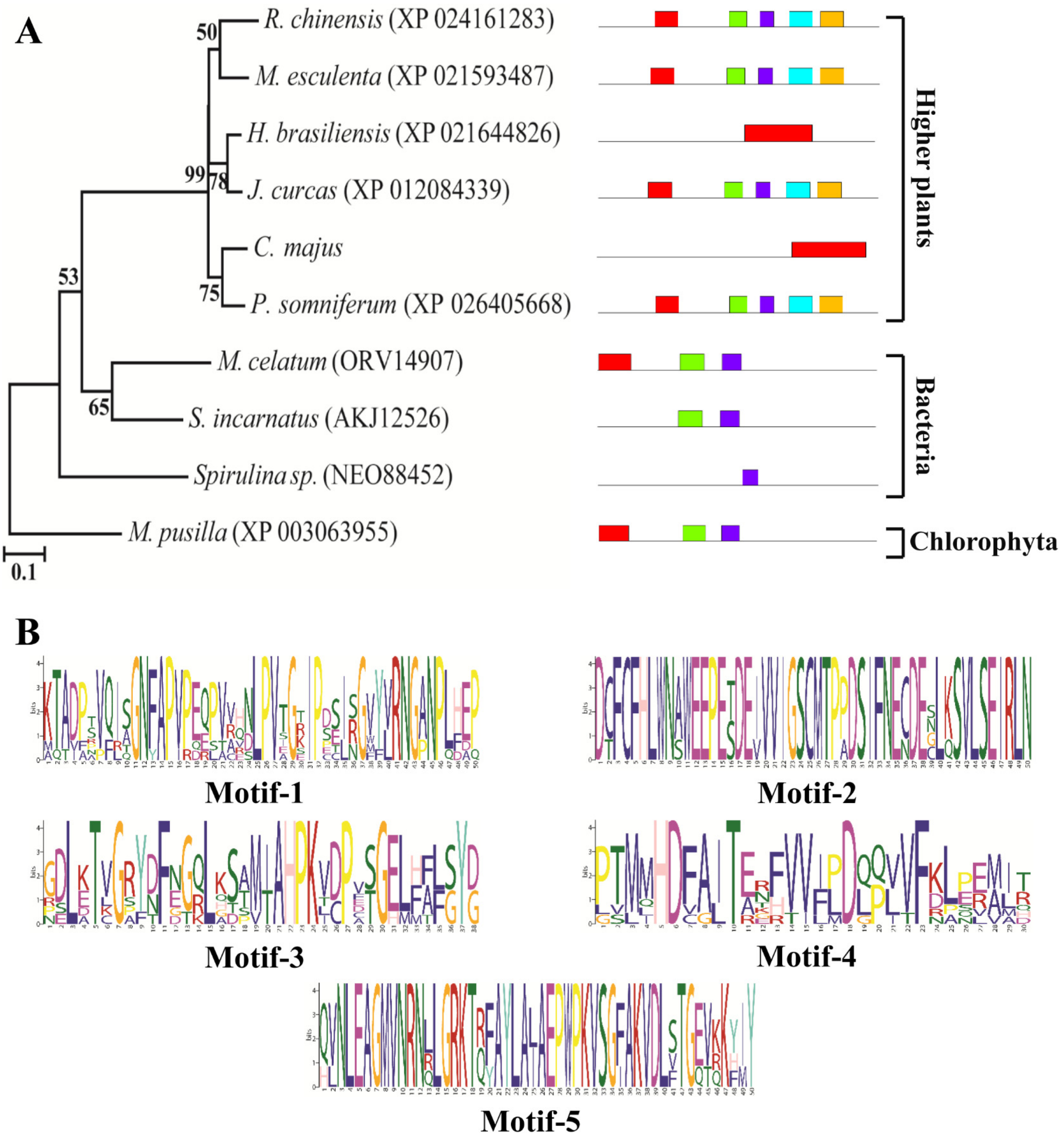


**Figure S2N:** Phylogeny of deduced CmNCED amino acid sequences along with other NCED sequences. The tree was constructed using MEGA 7 [116] on the basis of the Neighbor–Joining method [117]. Bootstrap values on the nodes indicate the number of times each group occurred within 1000 replicates [118]. On the right side, the analysis of conserved protein motifs using MEME software is presented, showing motifs and are ordered in colored boxes corresponding to NCED sequence in the phylogenetic tree. Other protein sequence obtained from NCBI database as well as their accession numbers are provided in the bracket. (B) Conserved protein motif 1-5 present in the variable region of CmNCED gene. The height of the letters denotes the degree of conservation.

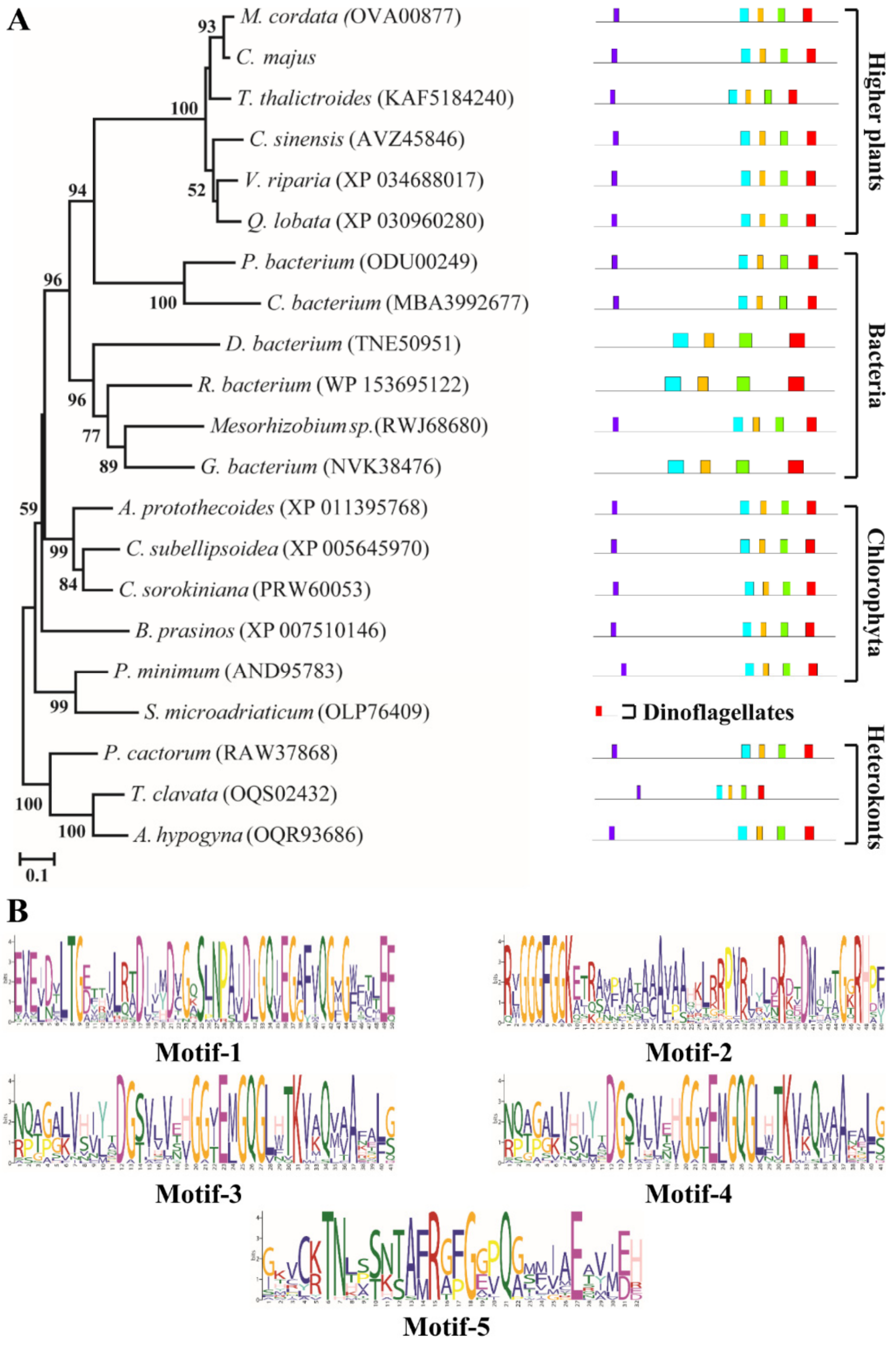


**Figure S2O:** Phylogeny of deduced CmAO amino acid sequences along with other AO sequences. The tree was constructed using MEGA 7 [116] on the basis of the Neighbor–Joining method [117]. Bootstrap values on the nodes indicate the number of times each group occurred within 1000 replicates [118]. On the right side, the analysis of conserved protein motifs using MEME software is presented, showing motifs and are ordered in colored boxes corresponding to AO sequence in the phylogenetic tree. Other protein sequence obtained from NCBI database as well as their accession numbers are provided in the bracket. (B) Conserved protein motif 1-5 present in the variable region of CmAO gene. The height of the letters denotes the degree of conservation.

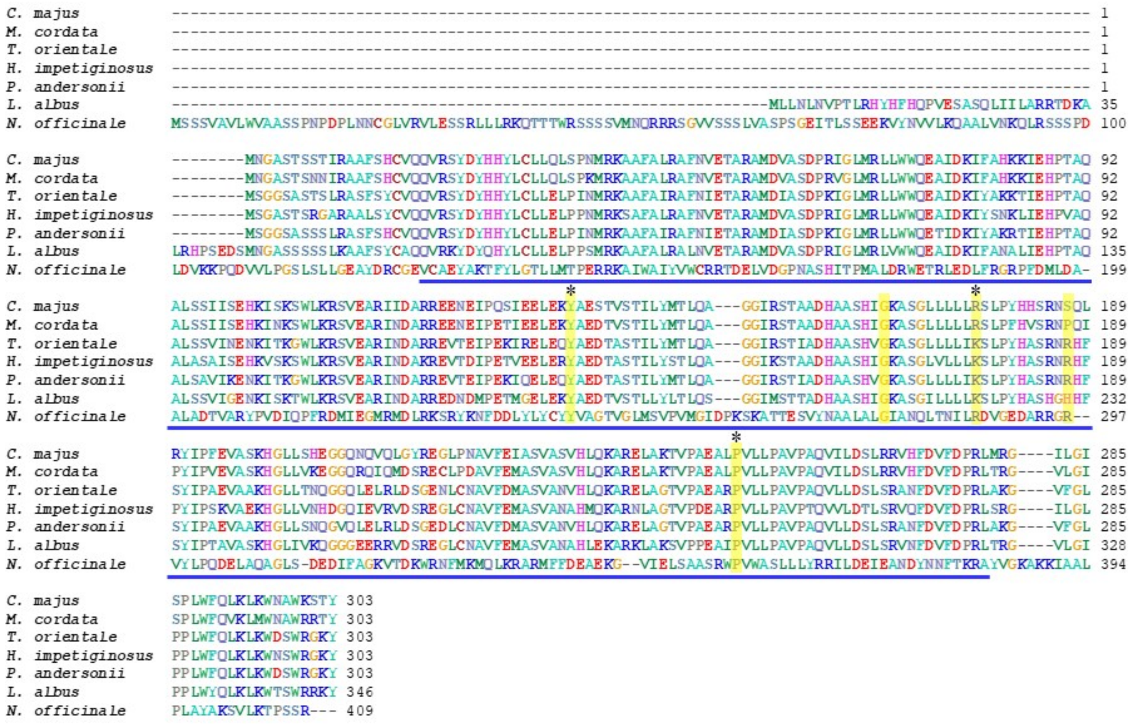

**Figure S3A.** Amino acid alignment of CmPSY with selected corresponding genes was performed with the BioEdit program of ClustalW. The yellow highlighted box represents the substrate-binding pocket. Asterisks indicate catalytic residues, and those with a blue underline represent the conserved trans-isoprenyl diphosphate synthase domain. *M. cordata* (*Macleaya cordata*, OVA08062), *T. orientale* (*Trema orientale*, PON92947), *H. impetiginosus* (*Handroanthus impetiginosus*, PIN12720), *P. andersonii* (*Parasponia andersonii*, PON53814), *L. albus* (*Lupinus albus*, KAE9613159), and *N. officinale* (*Nasturtium officinale*, MT547989) were involved in amino acid alignment of CmPSY. A dash (–) represents an aligned gap.

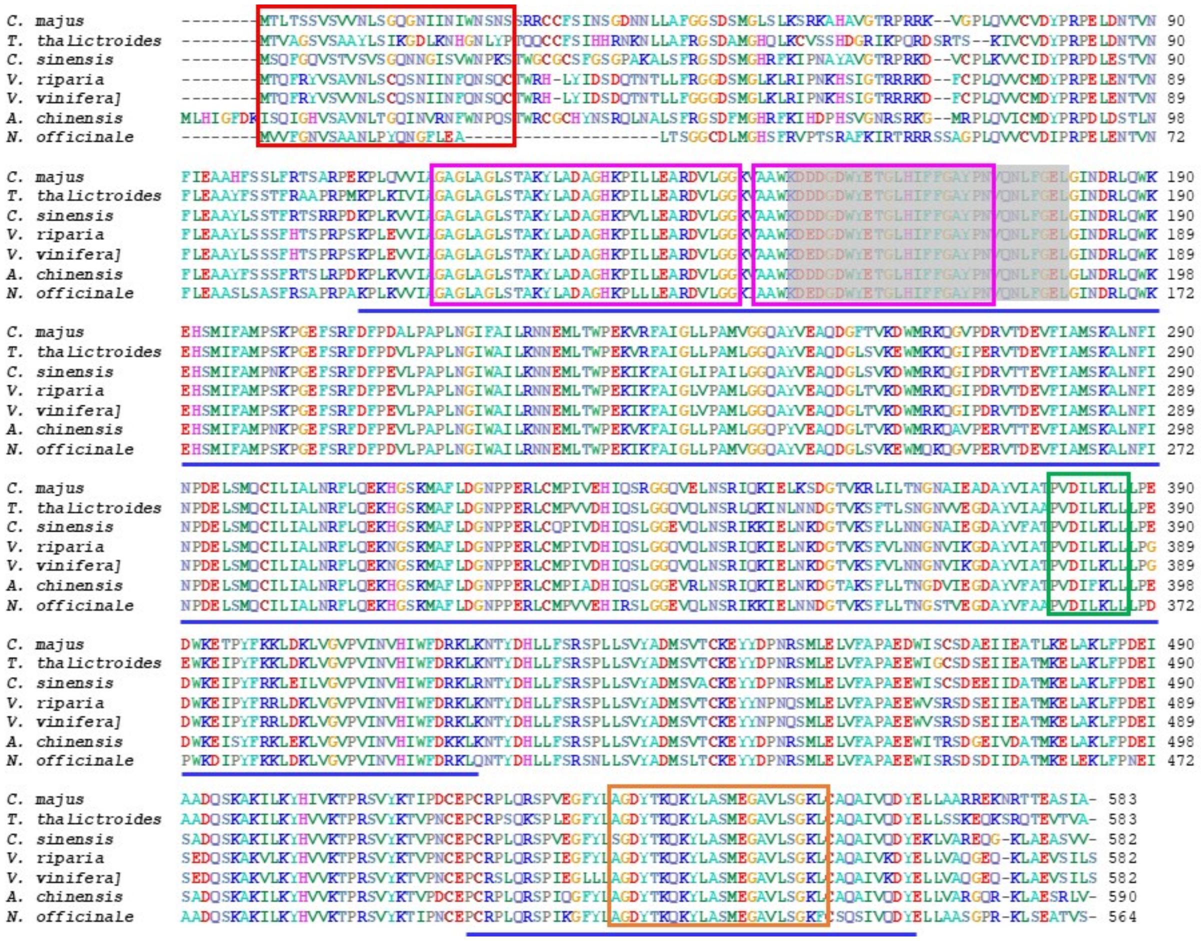

**Figure S3B.** Amino acid alignment of CmPDS with selected corresponding genes was performed with the BioEdit program of ClustalW. The red, pink, green, and orange boxes represent the signal peptide, dinucleotide-binding motif, putative substrate carrier motif, and carotenoid binding motif, respectively. A highly conserved region of the PDS protein was highlighted in an ash color. The blue underline represents the FAD/NAD(P)-binding domain. *T. thalictroides* (*Thalictrum thalictroides*, KAF5198369), *C. sinensis* (*Camellia sinensis*, XP028107084), *V. riparia* (*Vitis riparia*, XP034695872), *V. vinifera* (*Vitis vinifera*, AFP28796), *A. chinensis* (*Actinidia chinensis*, PSR96231), and *N. officinale* (*Nasturtium officinale*, MT547988) were involved in amino acid alignment of CmPDS. A dash (–) represents an aligned gap.

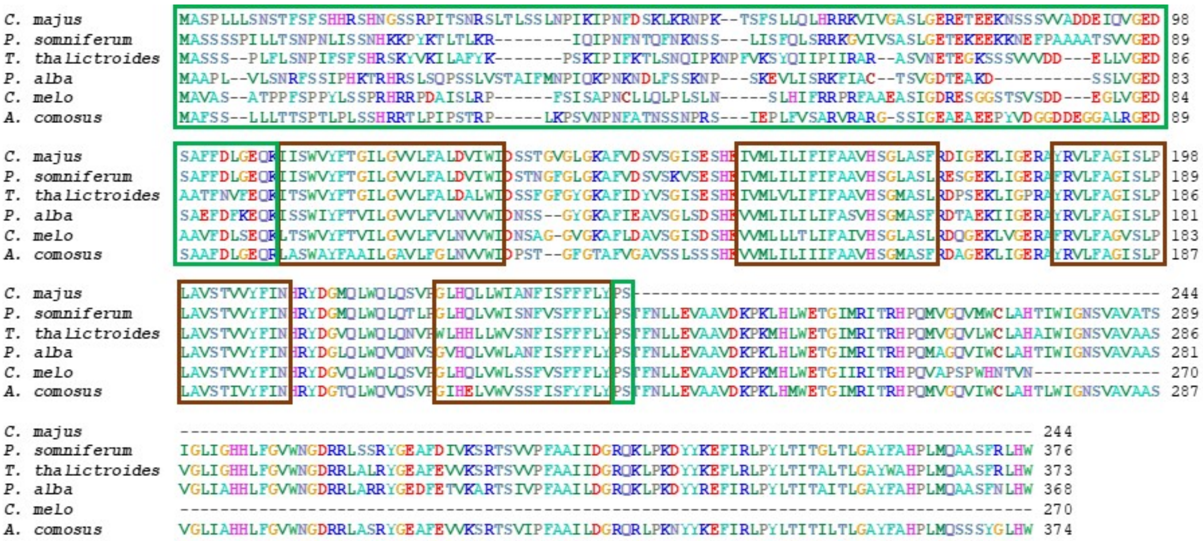

**Figure S3C.** Amino acid alignment of CmZ-ISO with selected corresponding genes was performed with the BioEdit program of ClustalW. Green and brown boxes represent the cytoplasmic domain and predicted trans membrane (TM) helix, respectively. *P. somniferum* (*Papaver somniferum*, XP026389168), *T. thalictroides* (*Thalictrum thalictroides*, KAF5176887), *P. alba* (*Prosopis alba*, XP028759619), *C. melo* (*Cucumis melo*, TYK16983), and *A. comosus* (*Ananas comosus*, XP020111958) were involved in amino acid alignment of CmZ-ISO. A dash (–) represents an aligned gap.

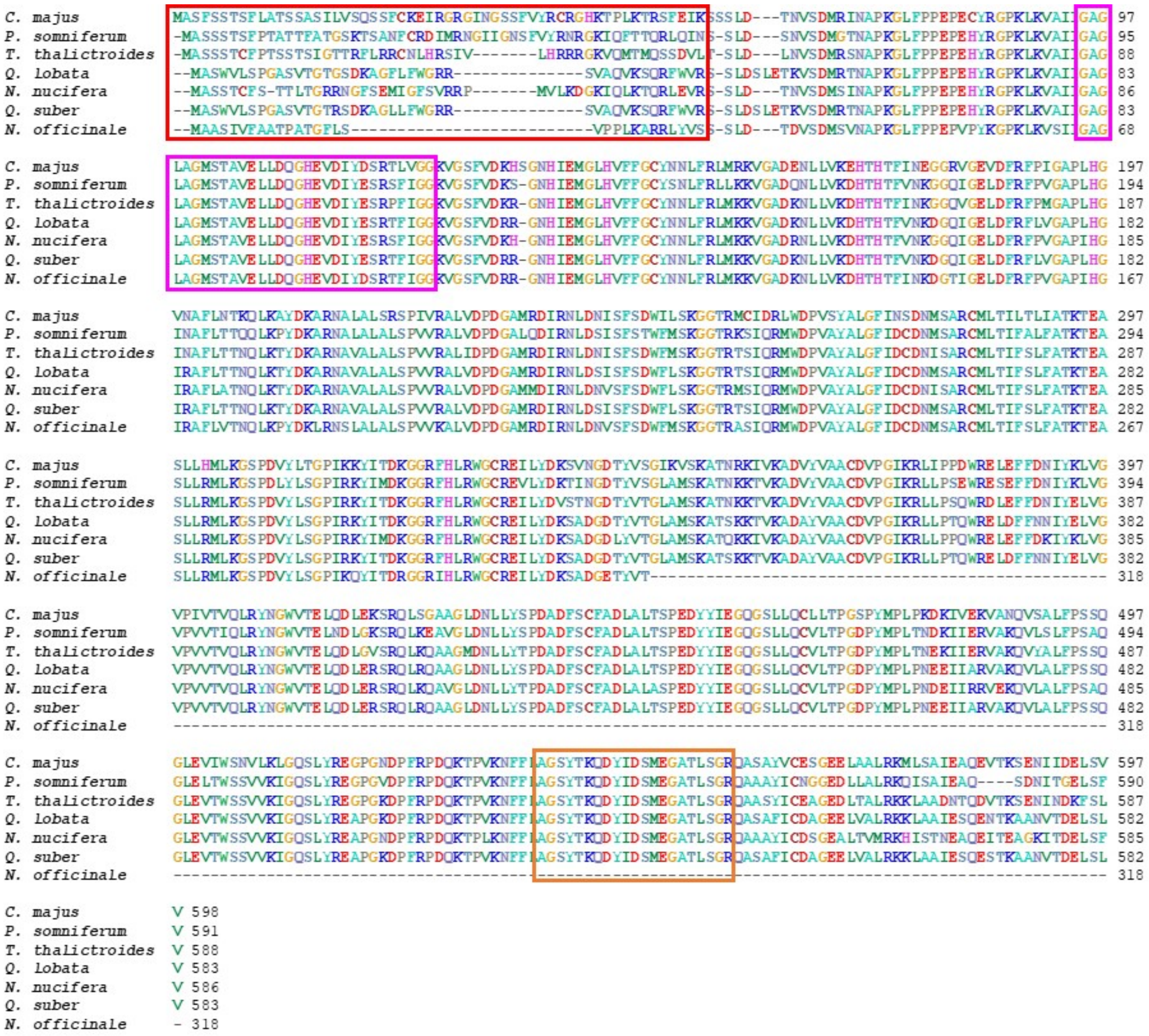

**Figure S3D.** Amino acid alignment of CmZDS with selected corresponding genes was performed with the BioEdit program of ClustalW. The red, pink, and orange boxes represent the signal peptide, dinucleotide-binding motif, and carotenoid binding motif, respectively. *P. somniferum* (*Papaver somniferum*, XP026435023), *T. thalictroides* (*Thalictrum thalictroides*, KAF5193496), *Q. lobate*, XP030958095 (*Quercus lobate*), *N. nucifera* (*Nelumbo nucifera*, XP010256022), *Q. suber* (*Quercus suber*, XP23871372), and *N. officinale* (*Nasturtium officinale*, MT547991) were involved in amino acid alignment of CmZDS. A dash (–) represents an aligned gap.

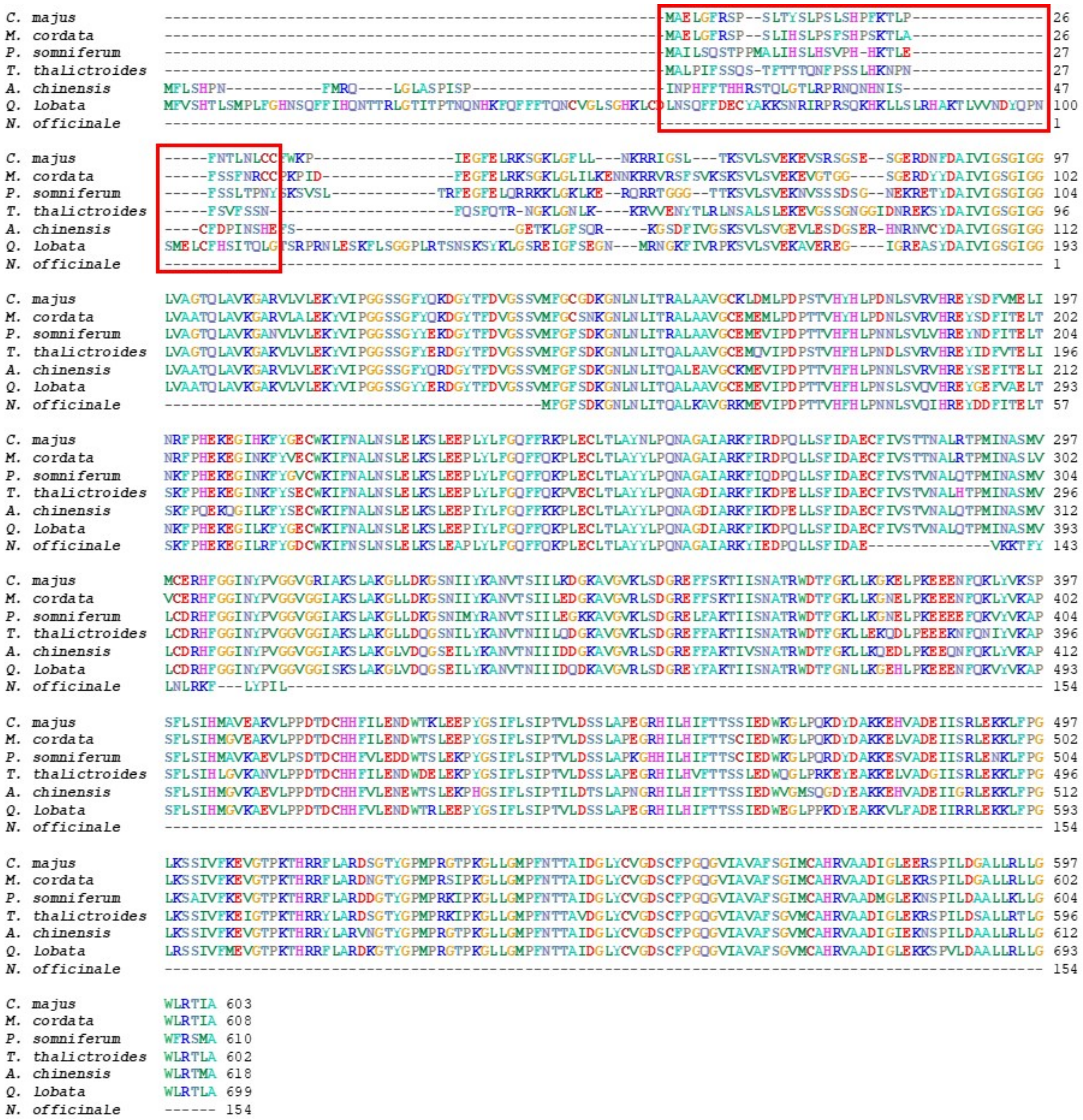


**Figure S3E.** Amino acid alignment of NoCrtISO with selected corresponding genes was performed with the BioEdit program of ClustalW. The red box represents the signal peptide. *M. cordata* (*Macleaya cordata*, OVA16223), *P. somniferum* (*Papaver somniferum*, XP026426554), *T. thalictroides* (*Thalictrum thalictroides*, KAF5180293), *A. chinensis* (*Actinidia chinensis*, PSS15835), *Q. lobate* (*Quercus lobate*, XP030927457), and *N. officinale* (*Nasturtium officinale*, MT547983) were involved in amino acid alignment of CmCrtISO. A dash (–) represents an aligned gap.

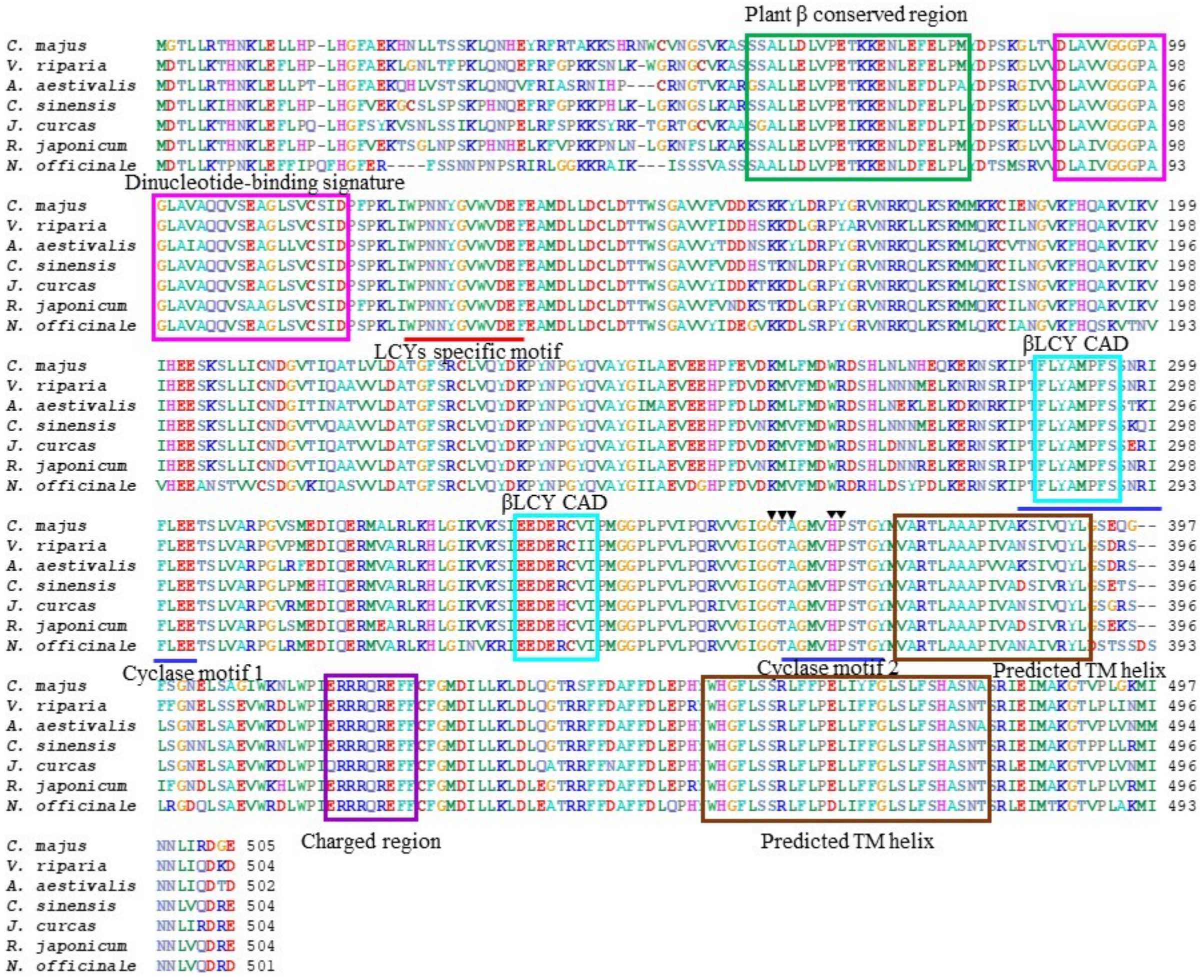


**Figure S3F.** Amino acid alignment of CmLCYB with selected corresponding genes was performed with the BioEdit program of ClustalW. The green, pink, light blue, brown, and purple boxes represent plant β conserved region, dinucleotide-binding motif, βLCY catalytic domain (CAD), predicted trans membrane (TM) helix, and charged region, respectively. The red underlined indicates LCY specific motif, whereas the blue underlined indicates cyclase motif 1 and 2. The highlighted arrowheads indicate the regulatory motif conserved in all β-bicyclase. *V. riparia* (*Vitis riparia*, XP034694605), *A. aestivalis* (*Adonis aestivalis*, AAK07430), *C. sinensis* (*Camellia sinensis*, AJB84622), *J. curcas* (*Jatropha curcas*, XP012085981), *R. japonicum* (*Rhododendron japonicum*, BAS69435), and *N. officinale* (*Nasturtium officinale*, MT547985) were involved in amino acid alignment of CmLCYB. A dash (–) represents an aligned gap.

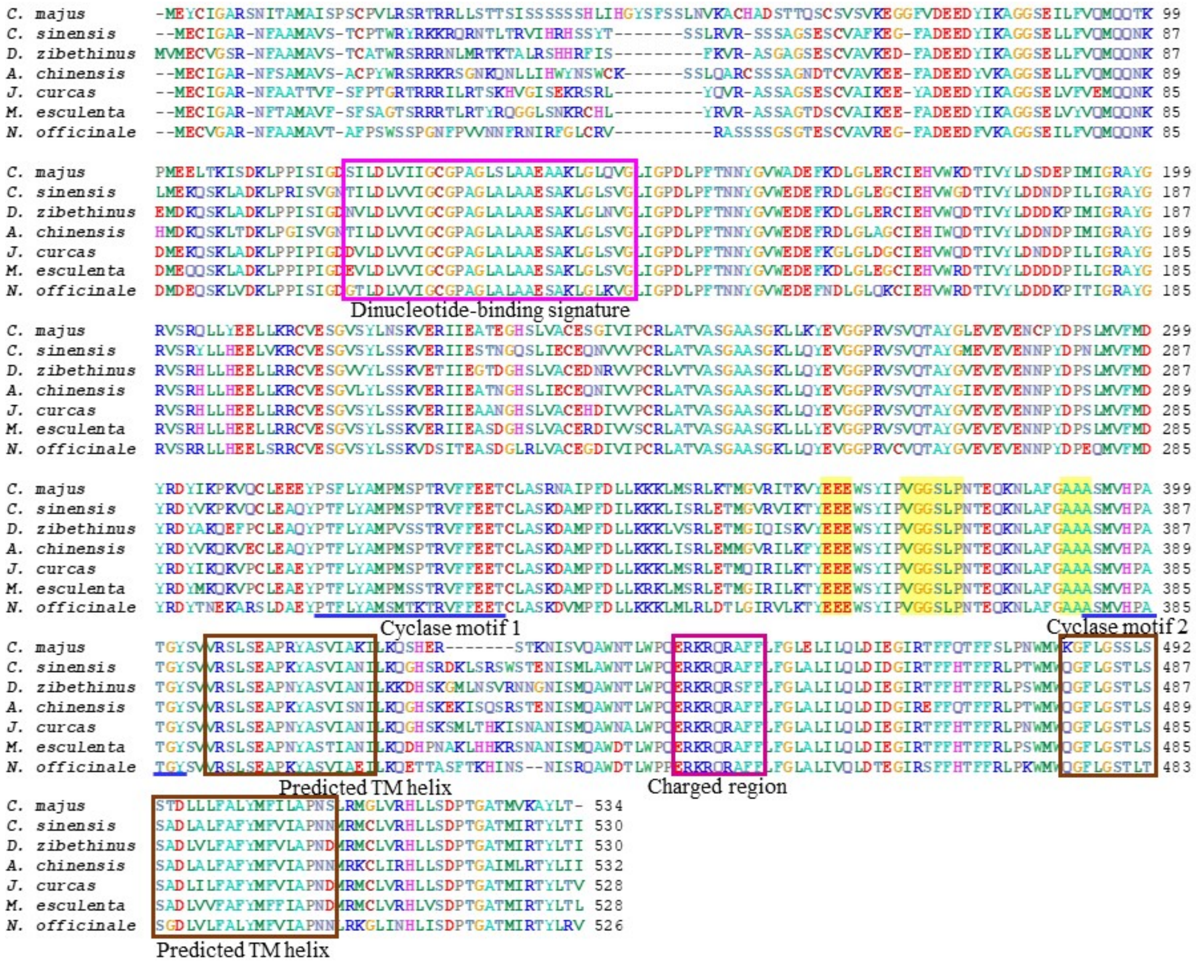


**Figure S3G.** Amino acid alignment of CmLCYE with selected corresponding genes was performed with the BioEdit program of ClustalW. Pink, brown, and purple boxes represent the dinucleotide-binding motif, predicted trans membrane (TM) helix, and charged region, respectively, and those with a blue underline represent cyclase motif 1 and 2. Yellow highlighted box indicate the conserved motif. *C. sinensis* (*Camellia sinensis*, XP028074216), *D. zibethinus* (*Durio zibethinus*, XP022751743), *A. chinensis* (*Actinidia chinensis*, PSS20792), *J. curcas* (*Jatropha curcas*, XP012076949), *M. esculenta* (*Manihot esculenta*, XP021596942), and *N. officinale* (*Nasturtium officinale*, MT547986) were involved in amino acid alignment of CmLCYE. A dash (–) represents an aligned gap.

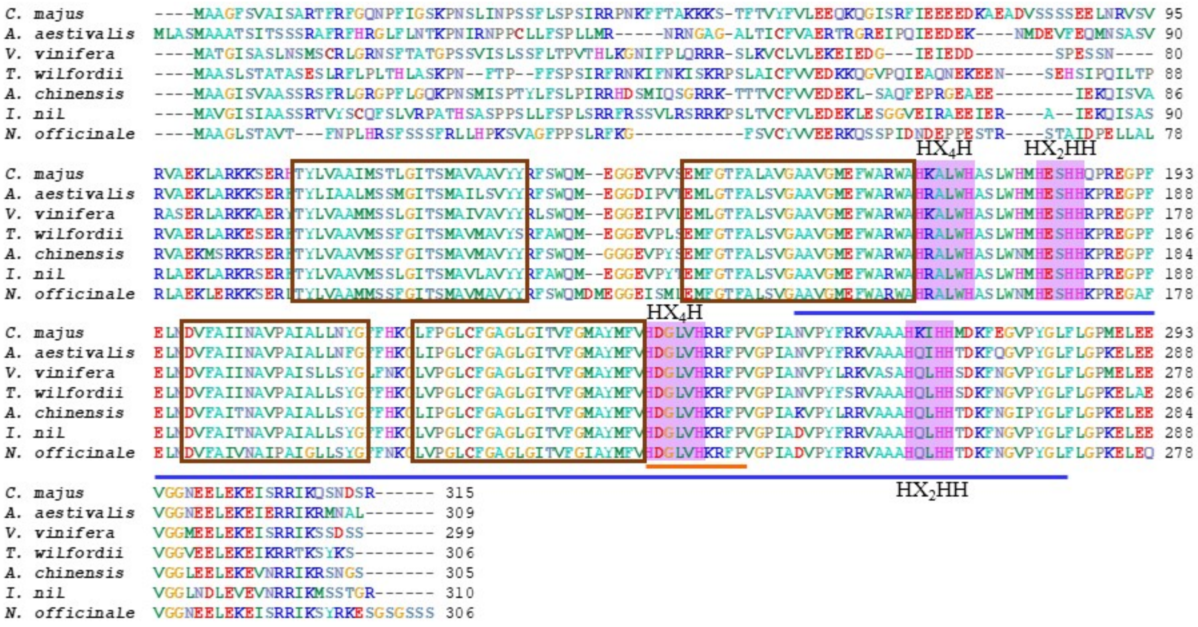


**Figure S3H.** Amino acid alignment of CmCHXB with selected corresponding genes was performed with the BioEdit program of ClustalW. The brown boxes indicate the transmembrane region. The histidine domain of the CHXB protein was highlighted in pink color. Blue and red underlined indicate the fatty acid hydroxylase domain and highly conserved region (motif 1). *A. aestivalis* (*Adonis aestivalis*, ABL67481), *V. vinifera* (*Vitis vinifera*, AAM77007), *T. wilfordii* (*Tripterygium wilfordii*, KAF5731165), *A. chinensis* (*Actinidia chinensis*, PSS04390), *I. nil* (*Ipomoea nil*, BAI47580), and *N. officinale* (*Nasturtium officinale*, MT547981) were involved in amino acid alignment of CmCHXB. A dash (–) represents an aligned gap.

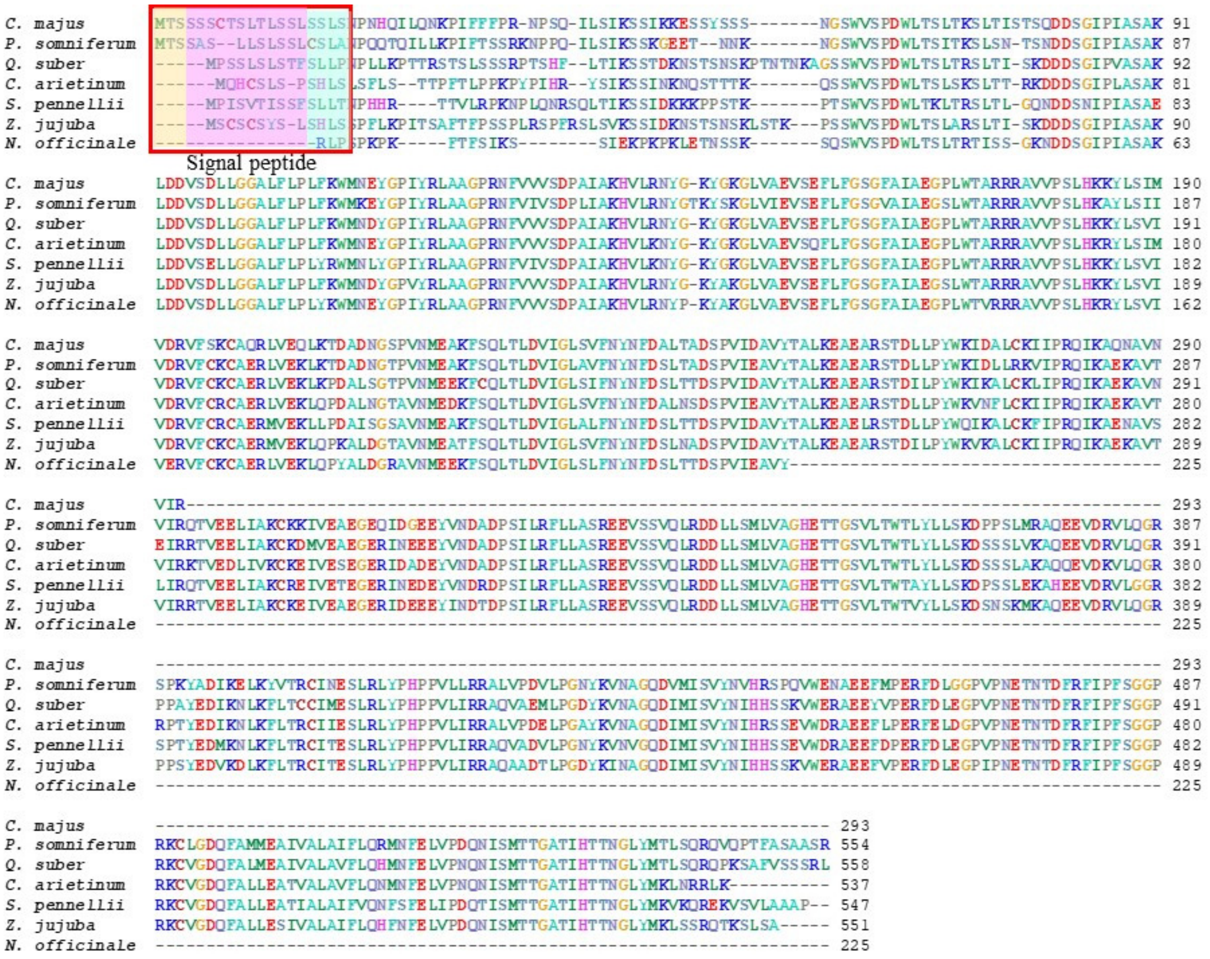


**Figure S3I.** Amino acid alignment of CmCHXE with selected corresponding genes was performed with the BioEdit program of ClustalW. The red boxes represent the signal peptide. The blue, yellow, and pink color highlighted denotes signal peptide C, N, and H regions of the CHXE protein, respectively. *P. somniferum* (*Papaver somniferum*, XP026417943), *Q. suber* (*Quercus suber*, XP023872296), *C. arietinum* (*Cicer arietinum*, XP004516959), *S. pennellii* (*Solanum pennellii*, XP015056054), *Z. jujuba* (*Ziziphus jujuba*, XP015898578), and *N. officinale* (*Nasturtium officinale*, MT547982) were involved in amino acid alignment of CmCHXE. A dash (–) represents an aligned gap.

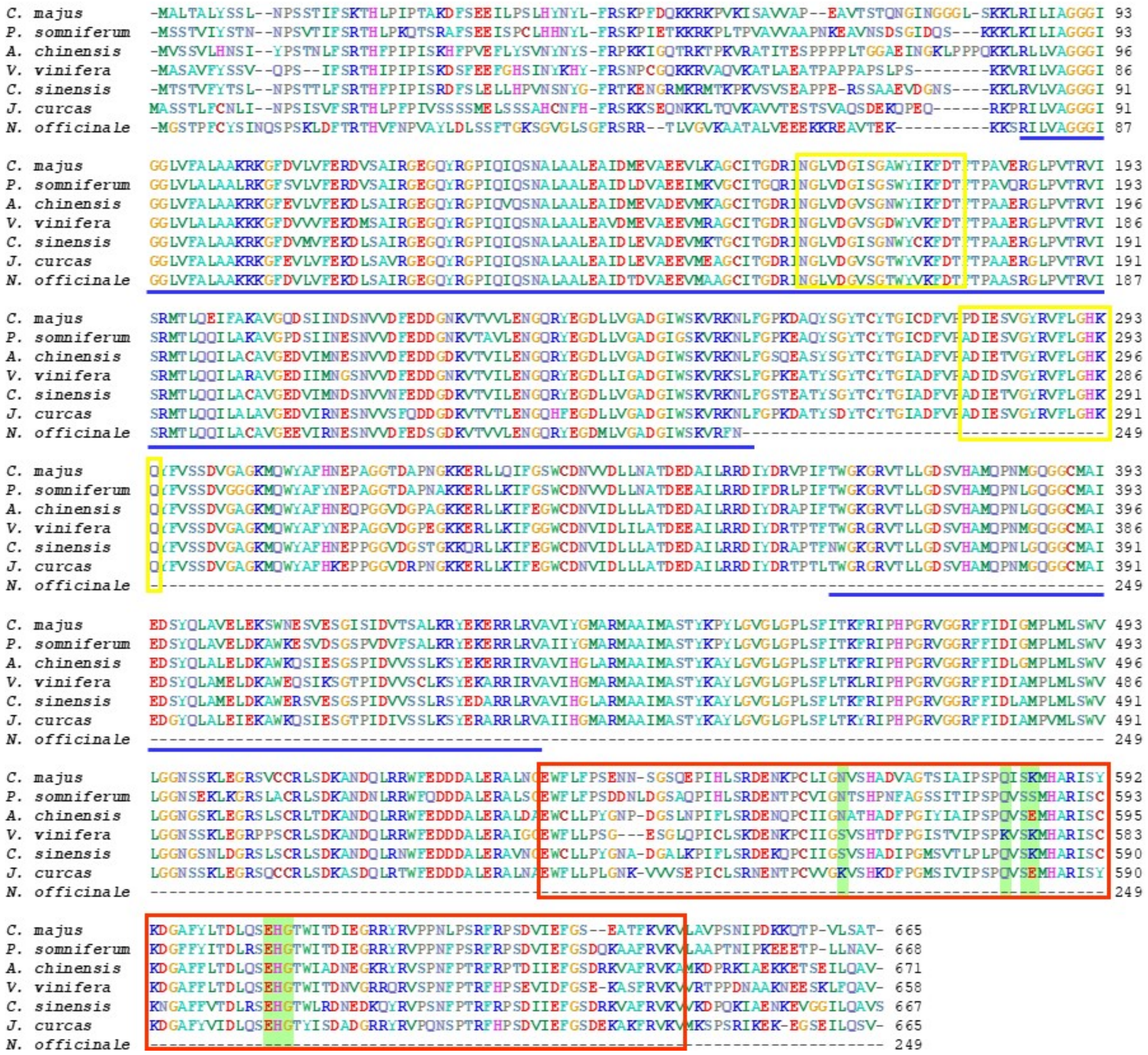

**Figure S3J.** Amino acid alignment of CmZEP with selected corresponding genes was performed with the BioEdit program of ClustalW. The yellow and red boxes represent the short motifs of lipocalin family proteins and forkhead-associated (FHA) domains, respectively. The phosphopeptide binding site of the ZEP protein was highlighted in green color. The blue underline represents the FAD-binding domain. *P. somniferum* (*Papaver somniferum*, XP_026445919), *A. chinensis* (*Actinidia chinensis*, PSS21278), *V. vinifera* (*Vitis vinifera*, NP_001268202), *C. sinensis* (*Camellia sinensis*, AJB84624), *J. curcas* (*Jatropha curcas*, XP_012079233), and *N. officinale* (*Nasturtium officinale*, MT547990) were involved in amino acid alignment of CmZEP. A dash (–) represents an aligned gap.

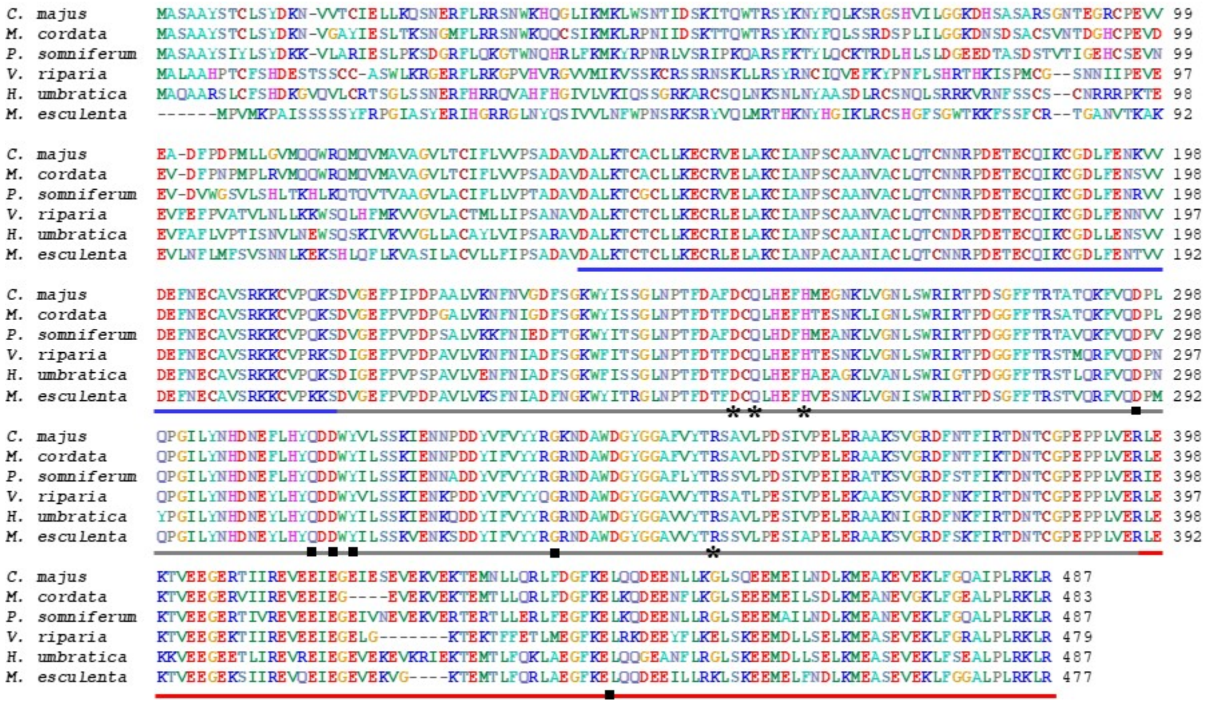

**Figure S3K.** Amino acid alignment of CmVDE with selected corresponding genes was performed with the BioEdit program of ClustalW. Blue, ash, and red underlined represent the VDE cys-rich, lipocalins, and Glu-rich domains, respectively. Asterisks indicate the important residues for pH switch, and those with black boxes represent the putative active site. *M. cordata* (*Macleaya cordata*, OVA03356), *P. somniferum* (*Papaver somniferum*, XP026400851), *V. riparia* (*Vitis riparia*, XP034683443), *H. umbratica* (*Herrania umbratica*, XP021282368), and *M. esculenta* (*Manihot esculenta*, XP021623227) were involved in amino acid alignment of CmVDE. A dash (–) represents an aligned gap.

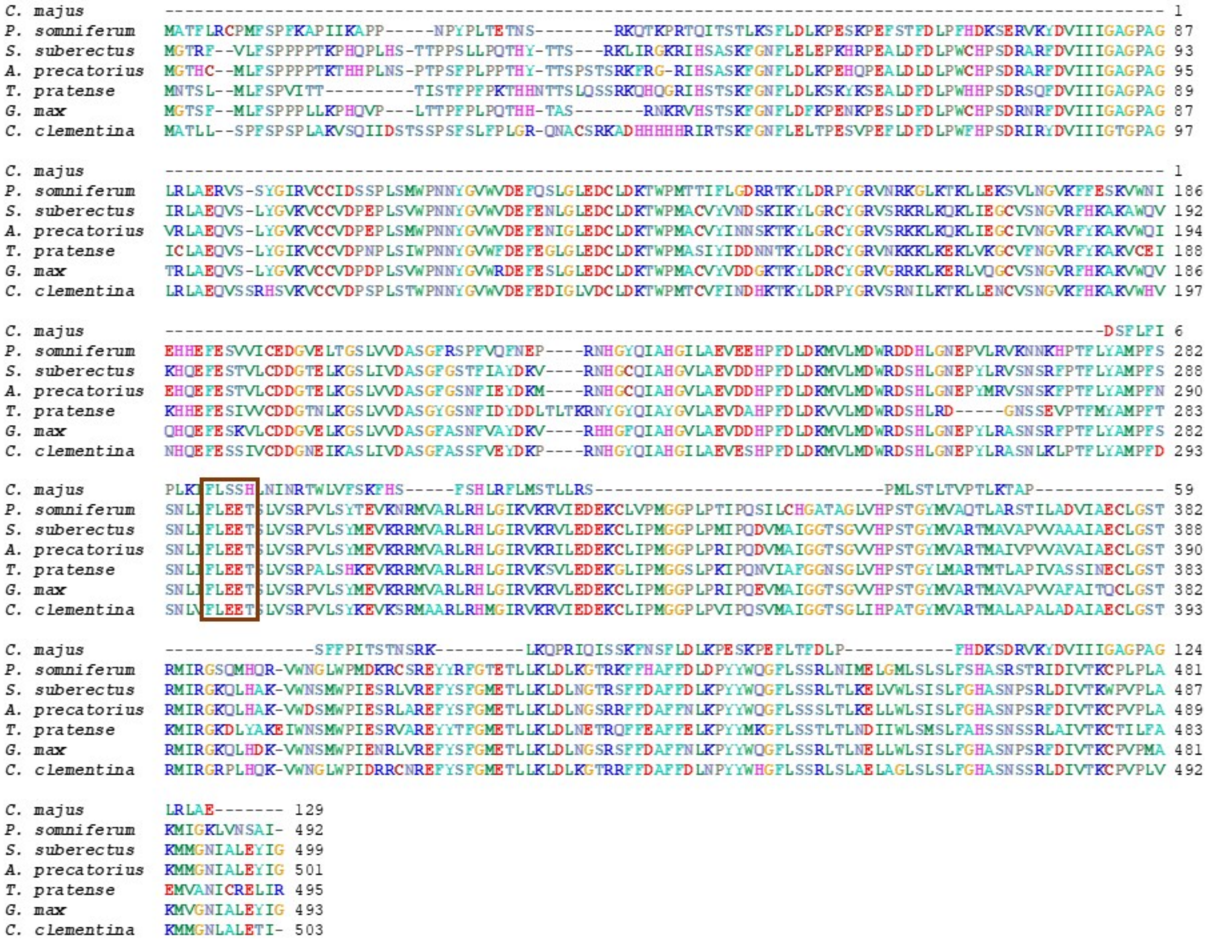

**Figure S3L.** Amino acid alignment of CmCCS with selected corresponding genes was performed with the BioEdit program of ClustalW. The brown boxes indicate the conserved FLEET motif essential for β and κ-cyclase activity. *P. somniferum* (*Papaver somniferum*, XP_026413514), *S. suberectus* (*Spatholobus suberectus*, TKY48659), *A. precatorius* (*Abrus precatorius*, XP_027344227), *T. pretense* (*Trifolium pretense*, PNY15080), *G. max* (*Glycine max*, XP_003541965), and *C. clementina* (*Citrus clementina*, XP_006424195) were involved in amino acid alignment of CmCCS. A dash (–) represents an aligned gap.

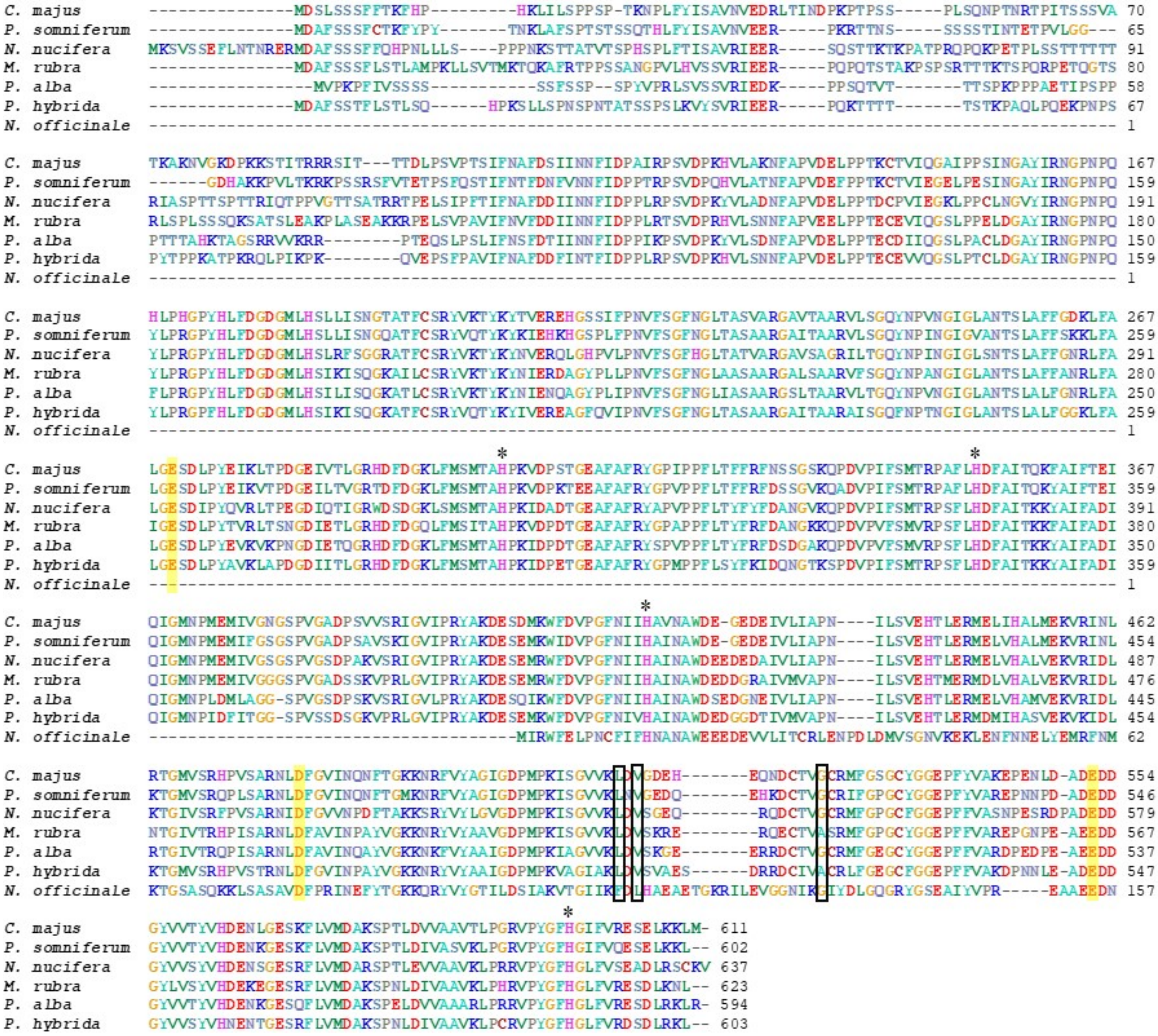

**Figure S3M.** Amino acid alignment of CmCCD with selected corresponding genes was performed with the BioEdit program of ClustalW. Asterisks indicate histidine residues involved in the coordination of the catalytic $Fe^{2+}$. The yellow highlighted boxes represent aspartate or glutamate residues which are predicted to be fixing the iron atom. The conserved amino acids present in the hydrophobic patches are shown in black boxes. *P. somniferum* (*Papaver somniferum*, XP_026380876), *N.o nucifera* (*Nelumbo nucifera*, XP_010275132), *M. rubra* (*Morella rubra*, KAB1215613), *P. alba* (*Prosopis alba*, XP_028782945), *P. hybrid* (*Petunia x hybrid*, QBC36243), and *N. officinale* (*Nasturtium officinale*, MT547984) were involved in amino acid alignment of CmCCS. A dash (–) represents an aligned gap.

```
C. majus          MPTSSST--------TWIN-KPSISINKPRRRSSSSTLSSSLQSPSLLHFPTQRHQSSPSTTTSTSAAISTYTHTHTPITLPKEKSKTLHPISSPNPNPN 91
P. somniferum     MSSSSVS--------SWVTNKPSISMSRNQRRSRNNLLSSSLQSPSLVQFTSS--SSSTSASISTYTHHTSSSSRTAQITLPKEKTKTLHLQS-----PT 85
R. chinensis      MATPSNSWAHTAQIQHHLSSSSSFSSLVNMG-FPTRSIALNRNRSSTIHSALQ---SPSVLHFPKQPYHPRVITKEATITPSPTPKTVTTTQQPP----- 91
H. brasiliensis   MASS------------AISMGSSISLDAPRA-STKLNISSSLHTPSILHFPKQ---SPRTTSFPPSPSISTTIPNKVTLPPAPSVDKSLAPEIN------ 78
M. esculenta      MASSSSLSPA-----AAISMSSSVSLDRP------SIISSSLQTPSIIHFPKH---SSTATSYPPSPSISTTIPKKIATT-LPSVDKSLAPVQN------ 79
J. curcas         MASTAP---------PAITMGSSISIGRP---SRKPNVTCSLQTPSILHFPKQ---STRTATYPPSSAISTSTTSNPINK-SSSTDISVPP--------- 75
N. officinale     MVSSPAK----------------------------------------------------------------------------------------------- 7

C. majus          PTPTTKTIQHPPNWNPFQKAASMALDAFETALLNKERQHPLPKTADPSVQISGNFAPVP-EQPVRQNLPVTGRIPCSISGFYVRNGANPLHEP------- 183
P. somniferum     ATPKTTKPQNQ-NWNIIQKVASMALDACETALLSKEKQQPLPKTADPSVQISGNFAPVP-EQPVCQNLPVTGSIPSSINGVYVRNGANPLFEPLSGHHLF 183
R. chinensis      ------------HWNLLQRAASTALDMLEGALVSRESQNPLPKTADPRVQIAGNFAPVP-EQPVRHSLPVSGTIPECISGVYVRNGANPLHEPVAGHHFF 178
H. brasiliensis   ------------QLNLLQRAAAMALDAVESALVSHENQYPLPKTADPTVQISGNFAPVP-EQPVVHNLPVAGKIPDSIRGVYLRNGANPLHEPVAGHHFF 165
M. esculenta      ------------QWNLLQKAAAMALDAVESALVSHERRFPLPKTADPAVQIAGNFAPVP-ERPVVHNLPVTGTIPDTIRGVYVRNGANPLHEPVAGHHFF 166
J. curcas         ------------EWNLLQKAASMVLDKVENVLVSHERKYPLPKTADPTVQISGNFAPVP-EQPVVRDLPVTGKIPDSIRGVYLRNGANPLHEPVTGHHFF 162
N. officinale     --------------QRPEMTLATALFTTVEDVINTFIDPPSRPSVDPKHVLSDNFAPVLDELPPTNCEIIHGSLPPSLDGAYIRNGPNPQFLPRGPYHLF 93

C. majus          ---------------------------------------------------------------------------------------------------- 183
P. somniferum     DGDGMIHAVNINQGSVSYSCRFTETHRLVQEKSLGRPVFPKAIGELHGHSG-IARLMLFYVRGAFGLVDPNQGIGVANAGLVYFNQRLLAMSEDDLPYQV 282
R. chinensis      DGDGMVHAVSINDGAASYACRFTETQRLVQEREIGRPVFPKAIGELHGHSG-IARLALFYARGALGLVDPSHGIGVANAGLVYFNDRLLAMSEDDMPYQV 277
H. brasiliensis   DGDGMVHAVRFENGSVSYACRFTETNRLVQERKLGRPDI------------------------------------------------------------- 204
M. esculenta      DGDGMVHAVRFEKGSVSYACRFTETNRLVQERELGRSVFPKAIGELHGHSG-IARLLLFCARGLFGIVDSSHGTGVANAGLVYFDGRLLAMSEDDLPYHV 265
J. curcas         DGDGMVHAVRFEEGSVSYACRFTETNRLVQERKLGRPIFPKAIGELHGHSG-IARLLLFYARGIFDIVDPSHGLGVANAGLVYFNGRLLAMSEDDLPYHV 261
N. officinale     DGDGMLHAIRIHGGKATLCSRYVKTYKYNVEKQSGAPVMVNVFSGFNGVTASVARGALTAARVLAGQYNPVNGIGLANTSLAFFSNRLFALGESDLPYAV 193

C. majus          ---------------------------------------------------------------------------------------------------- 183
P. somniferum     RITDSGDLETVGRFDFEGQLKSAMIAHPKLDPVSGELFALSYDVVQKPYLKYFRFSKNGEKSPDVEIP-LAQPTMMHDFAITENFVVIPDQQVVFKLP-- 379
R. chinensis      RITPSGDLETVGRYDFETQLGSTMIAHPKVDPVSGHLHTLSYDVVKRPYLKYFQFSADGEKSPDVEIP-LAAPTMMHDFAITENFVVIPDQQVVFKLQ-- 374
H. brasiliensis   ---------------------------------------------------------------------------------------------------- 204
M. esculenta      RVLPSGDLKTVGRYNFNGQLKSSMIAHPKVDPCSGELFALSYDVVQKPYLKYFRFSPDGKKSPDVDIS-LDQPTMMHDFAITERFVVIPDQQIVFKLP-- 362
J. curcas         RVLPCGDLKTVGRYNFNGQLKSTMIAHPKVDPFSGELFALSYDVVQKPYLKYFRFSPIGTKSKDVEIS-LDEPTMIHDFAITEKFVVIPDQQLVFKLP-- 358
N. officinale     RLTSDGDIETIGRHDFDGKLSMSMTAHPKIDPETGETFAFRYGPVP-PFLTYFRFDSSGKKQRDVPIFSMTSPSFIHDFAITKRHAIFSEIQLGMRMNPV 292

C. majus          ---------------------------------------------------------------------------------------------------- 183
P. somniferum     -EMIRGGSPVVYDKNKVARFGVLNKYAKDSSDMKWVEVPDCFCFHLWNSWEEPETDEIVVIGSCMTPADSIFNECDEGLQSVLSEIRLNLKTGKSTRRPI 478
R. chinensis      -EMITGGSPVIYDKNKKSRFGILAKNATNADDIIWVECPDTFCFHLWNAWEEPESDEVVVIGSCMTPPDSIFNENDECLKSVLSEIRLNLKTGESIRRPI 473
H. brasiliensis   ---------------------------------------------------------------------------------------------------- 204
M. esculenta      -EMIHGGSPVIYDKKKMSRFGVLDKKANDASKIIWVEAPDCFCFHLWNAWEEPETDEIVVIGSCMTPPDSIFNECDENLKSVLSEIRLNLKTGKSTRRPI 461
J. curcas         -EMIRGGSPVIYDENKISRFGILDKKANDSSKIRWIEAPDCFCFHLWNAWEEPESDEIVVIGSCMTPPDSIFNECDESLKSVLSEIRLNLKTGKSIRRPI 457
N. officinale     DLMIEGGSPVGTDTGKIPRLGVIQRYAGDESEMKWFEVPGFNIIHAINAWDEDDGNSIVLIAPNIMSIEHTLER-MDLVHALVEKVKIDLVTGIVTRHPI 391

C. majus          ---------------------------------------------------------------------------------------------------- 183
P. somniferum     ISEENQLN-LEAGMVNRNQLGRKTRFAYLAIAEPWPKVSGIAKVDLVTG-----EVQKFIYGDNKFGGEPYFVPN---SSSDREDDGHILAFMHDETTSK 569
R. chinensis      ISDESEHVNLEAGMVNRNRLGRKTRYAYLAIAEPWPKVSGFAKVDLFTG-----QVKKHIYGDRKFGGEPFFLPS---GSCGEEDGGYILSFVHDEKTWK 565
H. brasiliensis   ---------------------------------------------------------------------------------------------------- 204
M. esculenta      ISE-SEQVNLEAGMVNRNLLGRKTQFAYLALAEPWPKVSGFAKVDLSTG-----EVRKYMYG-NKYGGEPLFLPSDPSSNSGKEDSGYILCFVHDEKQGK 554
J. curcas         ISD-KHQVNLEAGMVNRNLLGRKTQFAYLALAEPWPKVSGFAKVDLSTG-----ETKKYIYGDNKYGGEPLFLPD---SNSGKEDDGFILCFVHDEKEWK 548
N. officinale     SAR-----NLDFGVINPAFLGRRSRYVYAGVGDPMPKISGVVKLDVSKGDREDCTVARRMYGPGCYGGEPFFVAKDPGNPEAEEDDGYVVTYVHDEASGE 486

C. majus          ------------------------------------------ 183
P. somniferum     SELLVINAMN--LELEASVKLPSRVPYGFHGTFINSMSLENQA 610
R. chinensis      SELQIVNAMN--LKLEATVTLPSRVPYGFHGTFIDSKDLKNQA 606
H. brasiliensis   ------------------------------------------ 204
M. esculenta      SELQIVNAMN--LKLEATVKLPSRVPYGFHGTFISAKDLEKQA 595
J. curcas         SELQIVNAKN--LQLEATVKLPSRVPYGFHGTFITAKDLEKQA 589
N. officinale     SKFLVMDAKSPELEIVAAVRLPRRVPYGFHGLFVKERDLNKL- 528
```

**Figure S3N.** Amino acid alignment of CmNCED with selected corresponding genes was performed with the BioEdit program of ClustalW. *P. somniferum* (*Papaver somniferum*, XP026405668), *R. chinensis* (*Rosa chinensis*, XP024161283), *H. brasiliensis* (*Hevea brasiliensis*, XP021644826), *M. esculenta* (*Manihot esculenta*, XP021593487), *J. curcas* (*Jatropha curcas*, XP012084339), and *N. officinale* (*Nasturtium officinale*, MT547987) were involved in amino acid alignment of CmNCED. A dash (–) represents an aligned gap.

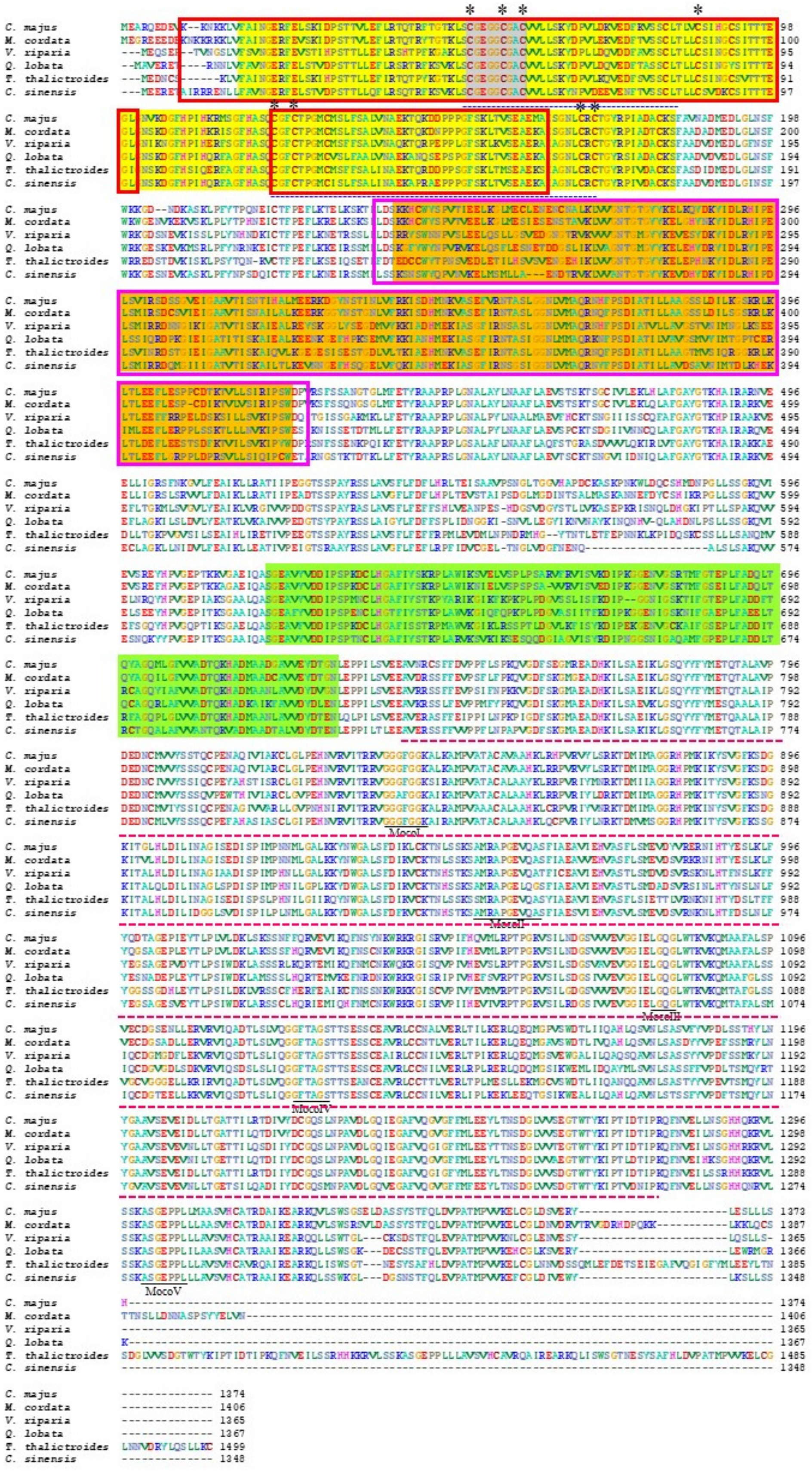

**Figure S3O.** Amino acid alignment of CmAO with selected corresponding genes was performed with the BioEdit program of ClustalW. The yellow, ash, orange, and green highlighted box represents the 2Fe-2S redox center-containing domain, 2Fe-2S ferredoxin-type iron-sulfur binding region, FAD-binding domain, and

hammer-head domain, respectively. Asterisks indicate the eight conserved cysteine residues of [2Fe-2S] centers, and those with a red and pink rectangle box represent 2Fe-2S ferredoxin-type iron-sulfur binding domain and FAD-binding domain. The dotted blue and red underlined indicates that motif corresponding two non-identical [2Fe-2S] redox centers and molybdenum cofactor and substrate-binding domain. The putative motifs involved in Moco and substrate binding (MocoI-MocoV) are underlined in black continuous line. *M. cordata* (*Macleaya cordata*, OVA00877), *V. riparia* (*Vitis riparia*, XP_034688017), *Q. lobate* (*Quercus lobate*, XP_030960280), *T. thalictroides* (*Thalictrum thalictroides*, KAF5184240), and *C. sinensis* (*Camellia sinensis*, AVZ45846) were involved in amino acid alignment of CmAO. A dash (-) represents an aligned gap.

**Table S1** List of primers designed and used in the qRT-PCR analysis to determine mRNA expression levels of *C. majus* CBP, XBP, and ABP genes

| Primer names | Primer sequence (5′→3′) | PCR product length (bp) |
|---|---|---|
| CmPSY-F | TGTTGAAGCTCGGATTATCGATG | 102 |
| CmPSY-R | AATATGGTGGAAACAGTGGATTCG | |
| CmPDS-F | AAGACCTGAACTTGACAATACCG | 130 |
| CmPDS-R | AGATAGACCAGCCAAGCCTG | |
| Cm-Z-ISO-F | GGTAATTGTAGGAGCATCGCTTG | 109 |
| Cm-Z-ISO-R | ATCAAAGAAAGCTGAATCCTCACC | |
| CmZDS-F | AGTGGGTTCCTTTGTCGATAAAC | 106 |
| CmZDS-R | GCCCACCTTTCTCATCAAGC | |
| CmCrtISO-F | TGACTTCGTCATGGAACTCATC | 117 |
| CmCrtISO-R | AGTGACTTCAATTCCAGCGAG | |
| CmLCYB-F | TGATCTGGCCTAACAATTACGG | 148 |
| CmLCYB-R | TTAACCCTTCCATAAGGTCTGTC | |
| CmLCYE-F | ATCTTGGGCTAGAAAGGTGTATTG | 106 |
| CmLCYE-R | CGACTAACTCTTCCATAAGCACG | |
| CmCHXB-F | GTACGAGTTGCAGAGAAATTAGCC | 115 |
| CmCHXB-R | TGTAATAAACAGCAGCAACAGCC | |
| CmCHXE-F | GTCAAGATGATTCAGGGATACCC | 131 |
| CmCHXE-R | AGCTGCAAGACGATAAATGGG | |
| CmZEP-F | TCACAAGGGTTATTAGCCGAATG | 150 |
| CmZEP-R | CTTCATATCGCTGTCCGTTCTC | |
| CmVDE-F | CTGTTGGGTGTTATGCAGCAG | 141 |
| CmVDE-R | CCTGCACTCTTTCAATAAACAAGC | |
| CmCCS-F | CATTCTTCCCAATCACTTCCAC | 104 |
| CmCCS-R | TTTGATTCAGGCTTCAGATCTAGG | |
| CmCCD-F | TCTAATGGCACTGCAACTTTCTG | 129 |
| CmCCD-R | AGCAACAGAAGCAGTGAGGC | |
| CmNCED-F | CACTCCAATCACTCTTCCCAAAG | 103 |
| CmNCED-R | GTGTTGGATTGTCTTTGTTGTTGG | |
| CmAO-F | TGAAATTGGAGCTGCTGTGAC | 128 |
| CmAO-R | ACGCAACCTTGTTCATATGGTC | |
| Cm$\alpha$-tubulin-F | TCCATCCTGAACAACTCATAAGTG | 112 |
| Cm$\alpha$-tubulin-R | TTTCTGATCCGATCAAGACACAG | |

**Table S2** Analysis of CBP gene sequences using the SignalP program

| Gene names | Original shearing site (C score) | Synthesized shearing site (Y score) | Signal peptide (S score) |
|---|---|---|---|
| CmPSY | 0.278 | 0.190 | 0.218 |
| CmPDS | 0.111 | 0.125 | 0.154 |
| CmZ-ISO | 0.119 | 0.124 | 0.183 |
| CmZDS | 0.250 | 0.265 | 0.515 |
| CmCrtISO | 0.635 | 0.260 | 0.221 |
| CmLCYB | 0.120 | 0.125 | 0.151 |
| CmLCYE | 0.194 | 0.141 | 0.186 |
| CmCHXB | 0.118 | 0.133 | 0.167 |
| CmCHXE | 0.128 | 0.176 | 0.304 |
| CmZEP | 0.116 | 0.118 | 0.135 |
| CmVDE | 0.158 | 0.124 | 0.144 |
| CmCCS | 0.116 | 0.158 | 0.400 |
| CmCCD | 0.107 | 0.126 | 0.156 |
| CmNCED | 0.107 | 0.106 | 0.126 |
| CmAO | 0.108 | 0.112 | 0.122 |
